# Trends in the incidence of diabetes mellitus: results from the Global Burden of Disease Study 2017 and implications for diabetes mellitus prevention

**DOI:** 10.1186/s12889-020-09502-x

**Published:** 2020-09-17

**Authors:** Jinli Liu, Zhen-Hu Ren, Hua Qiang, Jine Wu, Mingwang Shen, Lei Zhang, Jun Lyu

**Affiliations:** 1 China-Australia Joint Research Center for Infectious Diseases, School of Public Health, Xi’an Jiaotong University Health Science Center, Xi’an, Shaanxi 710061 PR China; 2 Department of Oral and Maxillofacial Surgery (Zhang Zhiyuan Academician Workstation), Hainan Western Central Hospital, Danzhou, 571700 China; 3 Department of Cardiovascular Medicine, the First Affiliated Hospital of Xi’an Jiaotong University, 277 Yanta West Road, Xi’an, 710061 Shaanxi China; 4 Melbourne Sexual Health Centre, Alfred Health, Melbourne, Australia; 5 Central Clinical School, Faculty of Medicine, Monash University, Melbourne, Australia; 6 Department of Epidemiology and Biostatistics, College of Public Health, Zhengzhou University, Zhengzhou, 450001 Henan China; 7 Department of Clinical Research, The First Affiliated Hospital of Jinan University, Guangzhou, 510630 China

**Keywords:** Global diabetes mellitus, Incidence, Trends, Prevention

## Abstract

**Backgroud:**

Diabetes mellitus is a common chronic disease and a severe public health issue. The incidence trends for type 1 diabetes (TIDM) and type 2 diabetes (T2DM) have rarely been studied on a global scale. We aimed to determine the temporal and geographical trends of diabetes globally.

**Methods:**

Data on diabetes mellitus, including incidence, prevalence from 1990 to 2017 were obtained from the 2017 Global Burden of Disease study. We calculated the estimated annual percentage changes (EAPCs) in age-standardized incidence rate (ASIR) of diabetes mellitus according to sex, region, and disease type.

**Results:**

The worldwide incident cases of diabetes mellitus has increased by 102.9% from 11,303,084 cases in 1990 to 22,935,630 cases in 2017 worldwide, while the ASIR increased from 234 /100,000 persons (95% UI, 219–249) to 285/100,000 persons (95% UI, 262–310) in this period [EAPC = 0.87, 95% confidence interval (CI):0.79–0.96]. The global ASIRs of T1DM and T2DM both demonstrated significant increase during 1990–2017, with EAPCs of 0.34 (95% CI,0.30–0.39) and 0.89 (95% CI,0.80–0.97), respectively. The ASIR trends also varied considerably by regions and countries. The increase in ASIR was greatest in high sociodemographic index regions (EAPC = 1.05, 95% CI:0.92–1.17) and lowest in low-SDI regions (EAPC = 0.79, 95% CI:0.71–0.88).

**Conclusions:**

Both the number of incident cases and ASIR of diabetes mellitus increased significantly during 1990–2017 worldwide, but the temporal trends varied markedly across regions and countries.

## Key messages

1. Both numbers of the incident cases and ASIR of diabetes mellitus increased from 1990 to 2017 at the global level.

2. This increasing pattern of diabetes mellitus was heterogeneous across regions and countries.

3. The ASIR of T1DM differed with latitude, and the farther away from the equator, the higher the ASIR in 2017.

## Backgroud

The incidence of diabetes have increased during recent decades [1, 2]. Studies have shown that the incidence of type 1 diabetes mellitus (T1DM) increased worldwide over the past 3 decades [3–5]. For example, the annual incidence of diabetes among youths increased from 9.0 cases per 100,000 person-year in 2002–2003 to 12.5 cases per 100,000 persons in 2011–2012 in the USA [6]. The incidence of T1DM differed significantly among European regions, being highest in central and eastern European countries in the 1990s [7]. The prevalence of being overweight or obese is also increasing worldwide [8]. The WHO Global Report on Diabetes indicated that being overweight or obese is the strongest risk factor for type 2 diabetes mellitus (T2DM) and that T2DM and prediabetes are increasingly being observed in children, adolescents, and younger adults [9]. Thus, the increase of overweight rate and obesity rate also affect the incidence of diabetes in different degrees. Diabetes can lead to complications in many parts of the body and can increase disability rates and the occurrence of other complications, resulting in a heavy economic burden. The highest proportion of health-care spending in the USA was on diabetes, costing an estimated $101.4 billion in 2013 [10]. The incidence of diabetes varies from region to region and is affected by many factors. The human development level of a country was measured using its human development index (HDI): a summary indicator of health, education, and income. The Human Development Index of a region may impact the diabetes incidence locally.

The Global Burden of Disease Study (GBD) has assessed the burden of diabetes mellitus in 194 countries (Taiwan: province of China) and territories around the world and hence provides a unique opportunity to understand the landscape of diabetes mellitus (See Additional file 1 Table S4 for details regarding 194 countries lists) [11]. In the present study, we retrieved detailed information on the incidence of diabetes mellitus from the GBD performed in 2017. We further assessed the disease burden of diabetes mellitus by determining temporal trends in the incidence of different types of diabetes mellitus from 1990 to 2017 at the global, regional, and national levels. The list of different types of diabetes mellitus has expanded, and many new and more detailed data sources incorporated. We reported the new findings for the first time at the country level for 1990–2017. The findings of the study can assist in the design of targeted strategies for diabetes mellitus prevention tailored to different countries.

### Methods

### Study data and definitions

According to sex, region, country, and disease type (T1DM and T2DM), the annual incident cases and age-standardized incidence of diabetes from 1990 to 2017 were derived using the Global Health Data Exchange (GHDx) query tool (http://gdx.healthdata.org/gbd-results-tool) [12]. GHDx is the world’s most comprehensive catalog of surveys, censuses, vital statistics, and other health-related data. The GBD 2017 study, which included injuries and risk factors, covered 195 countries and territories between 1990 and 2017. In total, 359 diseases and injuries and healthy life expectancy, 282 causes of death and 84 risk factors were systematically analyzed. Data used include vital registration systems, sample registration systems, household surveys (complete birth histories, summary birth histories, sibling histories), censuses (summary birth histories, household deaths), and Demographic Surveillance Sites. The explanation, validation and assessment of data quality have been reported in previous studies [13–15]. The SDI, which is based on national-level income per capita, average years of education among persons older than 15, and total fertility rate, was used to categorize the countries into five SDI quintiles (high, high-medium, medium, low-medium, and low levels). We also collected the human development index (HDI) data at the national level from the Human Development Reports of United nations development programme (http://hdr.undp.org/en/indicators/137506).

For the definition and classification of diabetes, we refer to GBD research criteria [13]. The case definitions and diagnostic criteria for overall diabetes mellitus, type 1 diabetes mellitus, and type 2 diabetes mellitus are presented in the Table 1 below.

**Table 1:** Overall diabetes mellitus, type 1 diabetes mellitus, and type 2 diabetes mellitus

Criterion	Definition
1. Overall diabetes mellitus	Diabetes mellitus (DM) is defined as fasting plasma glucose (FPG) > 126 mg/dL (7 mmol/L) or being on treatment for diabetes.
2. Overall diabetes mellitus type 1	Cases of DM that are on insulin or diagnosed with a biomarker (eg, c-peptide levels) that is not fasting plasma glucose
3. Overall diabetes mellitus type 2	Cases of diabetes mellitus (DM) type 2 are not reported as type 1 diabetes mellitus.

### Statistical analysis

Analyses were done separately for sex, region and country using a statistical model described and validated previously [16]. In addition, all the ages were included in the study. The model had a hierarchical structure in which estimates for each country, region and year were informed by its own data, data from other years in the same country and data in other countries in the same region. The model also accounted for non-linear time trends and age associations.

The incidence and prevalence are expressed as age-standardized based on the GBD reference population [13, 17, 18] unless otherwise specified. We used the age-standardized incidence rate (ASIR) and the estimated annual percentage change (EAPC) to quantify diabetes mellitus incidence trends [19]. The age-standardized prevalence rate (ASPR) were reported prevalence. ASR(age-standardized rate, include ASIR and ASPR) data can be obtained from the GHDx, with detailed calculation methods available in the literature [20]. Standardization was necessary for multiple groups of people with different age structures or for the same population in which the age distribution changes over time. The ASR (per 100,000 population) was calculated by summing up the products of the age-specific rates (*a*_*i*_, where *i* denotes the *i*^th^ age group) and the number of persons (or weight) (*w*_*i*_) in the same age group *i* of the chosen reference standard population, then dividing by the sum of the standard population weights:

$$ ASR=\frac{\sum_{i=1}^A{a}_i{w}_i}{\sum \limits_{i=1}^A{w}_i}\times \mathrm{100,000} $$

ASIR analysis can be used to better understand the disease models in the population and evaluate the effectiveness of current prevention strategies, and then develop more-targeted strategies where necessary. More importantly, the ASIR trends can serve as a good surrogate for shifting patterns of disease within a population, as well as provide clues about the changing risk factors. Consequently, the effectiveness of current prevention strategies can be assessed, and more-targeted ones can be established (if they are needed) based on the ASIR analyses [21].

The EAPC was a summary and widely used measure of the ASIR trend over a specified interval and determined by fitting a regression line to the natural logarithm of the ASIR: *y* = *α* + *βx* + *ε*, where *y* = ln(ASIR) and *x* = calendar year. The EAPC was calculated as 100 × (exp(*β*) – 1), and its 95% confidence interval (CI) can also be obtained from a linear regression model [22]. The calendar year was used as a continuous forecast variable. In the present study, the ASIR was deemed to be in an increasing trend if the EAPC and the lower boundary of its 95% CI were both > 0%. In contrast, the ASIR was in a decreasing trend if the EAPC estimation and the upper boundary of its 95% CI were both < 0%; otherwise, the ASIR was deemed to be uncertain over time. Additionally, in order to identify the factors influencing EAPCs, we evaluated the association between EAPC and the HDI in 2017 at the national level.

All statistical analyses were performed using the R program (version 3.5.1). A probability value of *p* < 0.05 was considered statistically significant.

## Results

### Global burden of diabetes mellitus

The worldwide incident cases of diabetes mellitus increased by 102.9%, from 11,303 × 10^3^ (95% UI, 10,582 × 10^3^–12,102 × 10^3^) in 1990 to 22,936 × 10^3^ (95% UI, 21,083 × 10^3^–25,041 × 10^3^) in 2017. The global ASIR increased from 234/100,000 persons (95% UI, 219–249) in 1990 to 285/100,000 persons (95% UI, 262–310) in 2017 (EAPC = 0.87, 95% CI: 0.79–0.96) (Table 2).

**Table 2:** The incident cases and age-standardized incidence rate (ASIR) of diabetes mellitus in 1990 and 2017, and its temporal trends from 1990 to 2017

Characteristics	1990		2017		1990–2017
	Incident cases	ASIR per 100,000	Incident cases	ASIR per 100,000	EAPC(%)^a^
	No. × 10^**3**^ (95% UI)	No.(95% UI)	No. × 10^**3**^ (95% UI)	No.(95% UI)	No.(95% CI)
Overall	11,303 (10582–12,102)	234 (219–249)	22,936 (21083–25,041)	285 (262–310)	0.87 (0.79–0.96)
Sex					
Male	5791 (5407–6214)	240 (225–256)	11,770 (10839–12,850)	295 (273–321)	0.89 (0.81–0.99)
Female	5512 (5162–5886)	227 (213–243)	11,166 (10244–12,217)	274 (252–299)	0.85 (0.77–0.94)
Tape					
Diabetes mellitus type 1	291 (263–323)	5 (5–6)	400 (362–442)	5 (5–6)	0.34 (0.30–0.39)
Diabetes mellitus type 2	11,013 (10283–11,811)	229 (214–244)	22,535 (20694–24,627)	279 (257–304)	0.89 (0.80–0.97)
Socio-demographic index					
Low	1102 (1019–1195)	227 (209–245)	2796 (2560–3054)	284 (259–311)	0.79 (0.71–0.88)
Low-middle	1901 (1759–2056)	234 (217–253)	4618 (4228–5032)	304 (278–332)	0.99 (0.92–1.07)
Middle	3049 (2834–3299)	225 (209–242)	6615 (6062–7248)	286 (262–311)	0.94 (0.85–1.02)
Middle-high	2544 (2365–2743)	234 (218–251)	4331 (3960–4766)	260 (239–284)	0.63 (0.48–0.78)
High	2661 (2511–2814)	234 (221–247)	4500 (4146–4910)	286 (265–310)	1.05 (0.92–1.17)
Region					
Asia Pacific–high income	443 (411–479)	221 (206–237)	611 (549–679)	230 (209–254)	0.29 (0.10–0.48)
Central Asia	181 (169–195)	310 (288–332)	350 (320–387)	376 (345–413)	0.81 (0.73–0.89)
East Asia	2262 (2067–2479)	180 (165–198)	3573 (3244–3984	202 (185–222)	0.82 (0.49–1.14)
South Asia	1824 (1677–1989)	212 (195–230)	4724 (4308–5182)	286 (260–313)	1.13 (0.97–1.29)
Southeast Asia	1090 (1014–1177)	285 (265–306)	2636 (2411–2889)	382 (350–417)	1.03 (0.94–1.13)
Australasia	50 (46–54)	220 (204–236)	79 (71–87)	209 (190–229)	−0.05(−0.17–0.07)
Caribbean	96 (91–102)	306 (290–323)	168 (155–183)	340 (314–369)	0.30 (0.27–0.33)
Central Europe	362 (339–389)	256 (240–273)	472 (428–516)	305 (280–333)	0.68 (0.63–0.72)
Eastern Europe	614 (565–662)	233 (215–251)	700 (630–776)	248 (226–273)	0.25 (0.21–0.29)
Western Europe	1174 (1093–1243)	236 (221–250)	1862 (1698–2055)	298 (273–326)	0.82 (0.78–0.86)
Andean Latin America	59 (55–62)	202 (190–215)	147 (135–160)	250 (230–273)	0.81 (0.77–0.84)
Central Latin America	434 (408–463)	341 (321–362)	978 (902–1064)	380 (351–413)	0.19 (0.08–0.30)
Southern Latin America	142 (132–151)	295 (274–314)	244 (221–267)	330 (301–361)	0.47 (0.43–0.52)
Tropical Latin America	272 (254–291)	217 (203–233)	494 (452–544)	206 (188–226)	−0.30(−0.40– −0.19)
North Africa and Middle East	733 (680–794)	291 (269–315)	2164 (1980–2370)	384 (351–420)	1.09 (1.03–1.15)
North America high-income	722 (676–772)	235 (219–251)	1518 (1402–1639)	317 (295–340)	1.98 (1.64–2.31)
Oceania	28 (26–30)	536 (501–579)	74 (68–81)	655 (604–712)	0.68 (0.56–0.80)
Central Sub-Saharan Africa	139 (128–151)	381 (352–413)	384 (353–423)	452 (413–494)	0.64 (0.60–0.68)
Eastern Sub-Saharan Africa	314 (291–339)	271 (251–291)	726 (664–797)	290 (264–317)	0.25 (0.23–0.27)
Southern Sub-Saharan Africa	127 (118–138)	329 (305–356)	311 (285–340)	453 (416–495)	1.33 (1.18–1.47)
Western Sub-Saharan Africa	237 (219–258)	184 (169–200)	721 (661–795)	251 (227–274)	1.14 (1.10–1.19)

*ASIR* age-standardized incidence rate, *CI* confidence interval, *EAPC* estimated annual percentage change, *UI* uncertainty interval

^a^The ASIR was deemed to be in an increasing trend if the EAPC and the lower boundary of its 95% CI were both > 0%;the ASIR was in a decreasing trend if the EAPC estimation and the upper boundary of its 95% CI were both < 0%; otherwise, the ASIR was deemed to be uncertain over time

The number of diabetes mellitus incident cases increased in both sexes from 1990 to 2017. The incident cases in males increased by 103.3%, from 5791 × 10^3^ (95% UI, 5407 × 10^3^–6214 × 10^3^) in 1990 to 11,770 × 10^3^ (95% UI, 10,839 × 10^3^–12,850 × 10^3^) in 2017, and the ASIR increased significantly with an EAPC of 0.89 (95% CI, from 0.81 to 0.99), rising from 240/100,000 persons (95% UI, 225–256) in 1990 to 295/100,000 persons (95% UI, 272–321) in 2017, while that in females increased by 102.6%, from 5512 × 10^3^ (95% UI, 5162 × 10^3^–5886 × 10^3^) in 1990 to 11,166 × 10^3^ (95% UI, 10,244 × 10^3^–12,217 × 10^3^) in 2017 and the ASIR increased from 227/100,000 persons (95% UI, 213–243) in 1990 to 274/100,000 persons (95% UI, 252–299) in 2017 and the ASIR increased by annually an average of 0.85 (0.77–0.94). Male had higher incient cases and ASIR than female in 1990 and 2017 (Table 2).

**Fig. 1:**
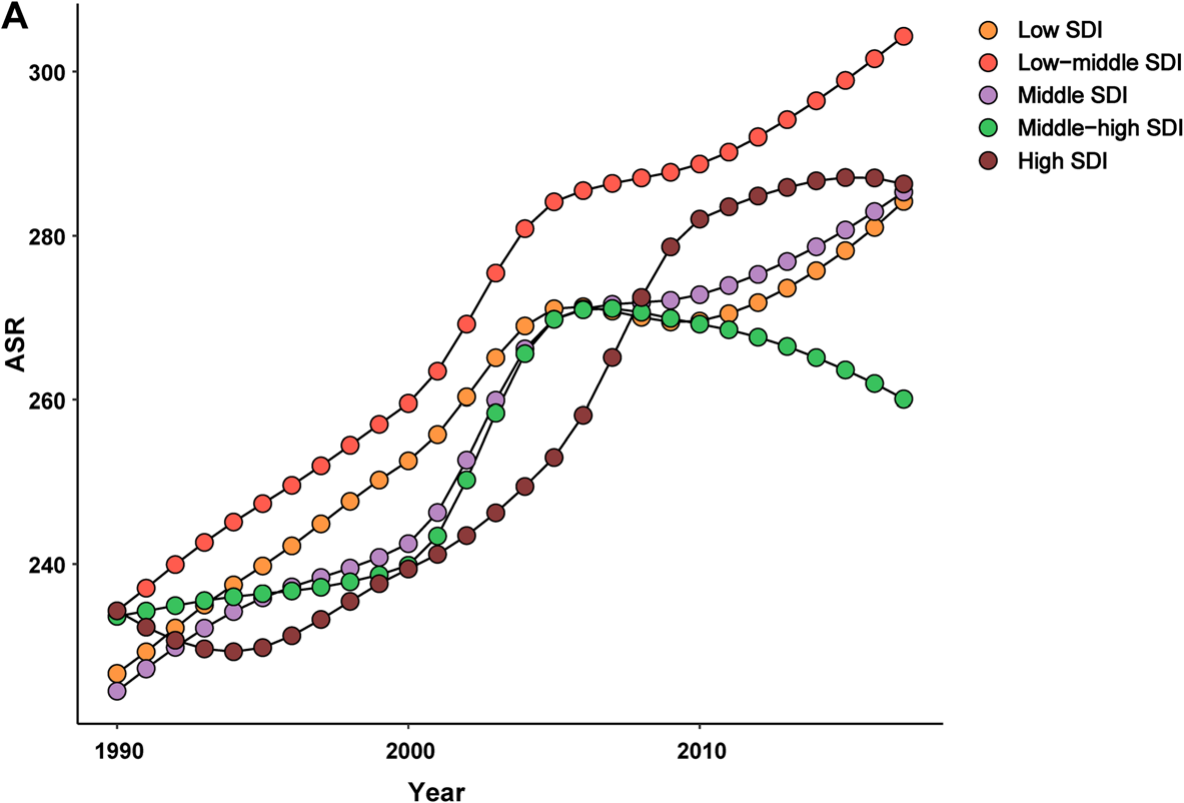
The ASIR of diabetes mellitus caused by SDI regions, from 1990 to 2017. The data from five SDI regions are presented in the top-right panel. (ASIR, age-standardized incidence rate; SDI, socio-demographic index)

**Fig. 2:**
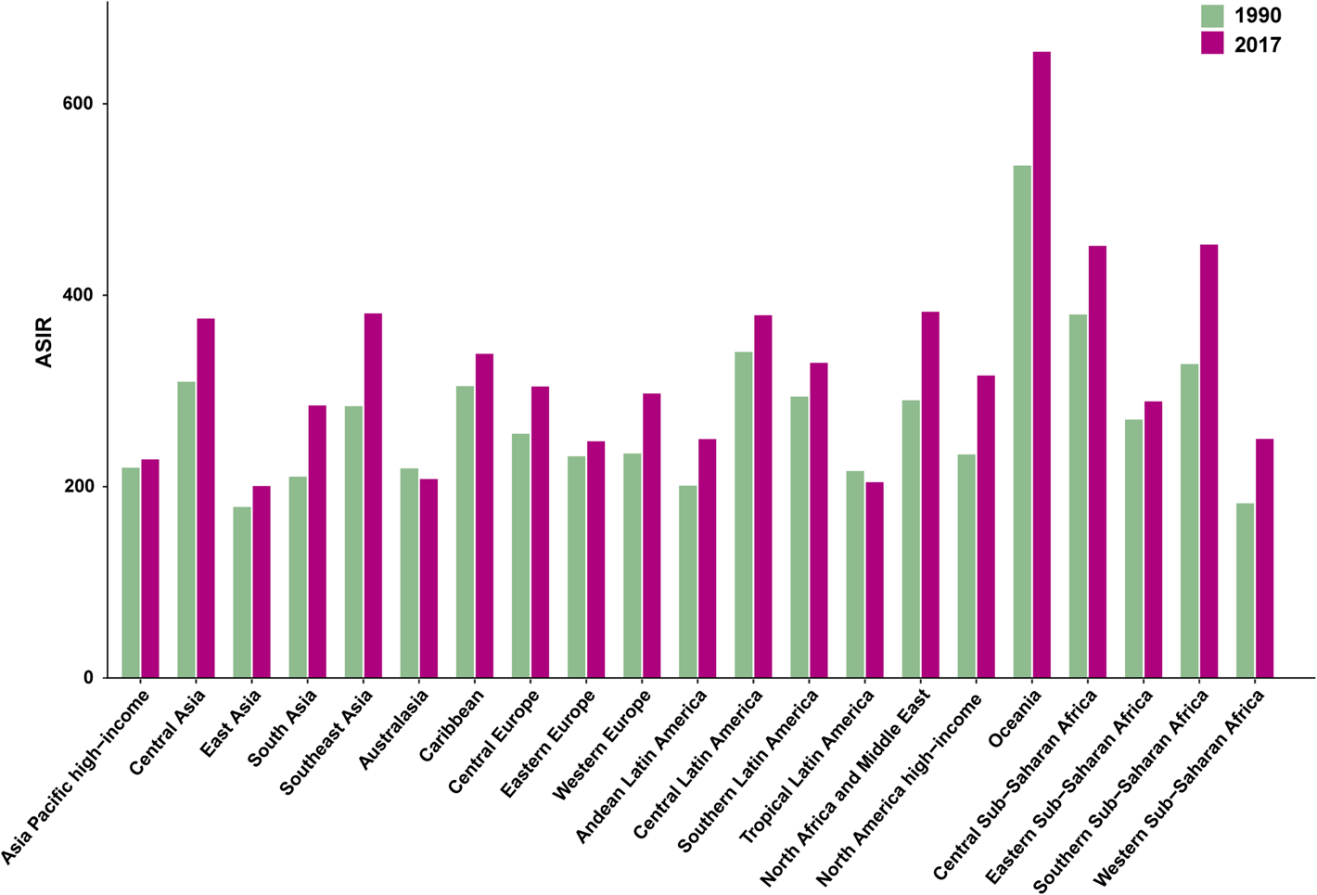
The ASIR of diabetes mellitus at a regional level. The left column in each group is case data in 1990 and the right column in 2017. Those data from certain regions can be viewed in the top-right of the panel (ASIR, age-standardized incidence rate)

At the regional level, the ASIR of diabetes mellitus increased across the five SDI regions from 1990 to 2017 (Fig. 1). The increase in ASIR was largest in high-SDI regions (EAPC = 1.05, 95% CI:0.92–1.17) and smallest in low-SDI regions (EAPC = 0.79, 95% CI:0.71–0.88) (Table 2). At the geographical level, the incident cases of diabetes mellitus increased from 1990 to 2017 in the 21 geographical regions (Table 2), with the increase being largest in western Sub-Saharan Africa (203.6%), and lowest Eastern Europe (14.0%). The incidence of diabetes mellitus increased from 1990 to 2017 in the 19 geographical regions (Fig. 2), The largest increase in ASIR was found in North America high-income (EAPC = 1.98, 95% CI:1.64–2.31), while the largest decrease was found in tropical Latin America (EAPC = − 0.30, 95% CI: from − 0.40 to − 0.19) (Table 2).

**Fig. 3:**
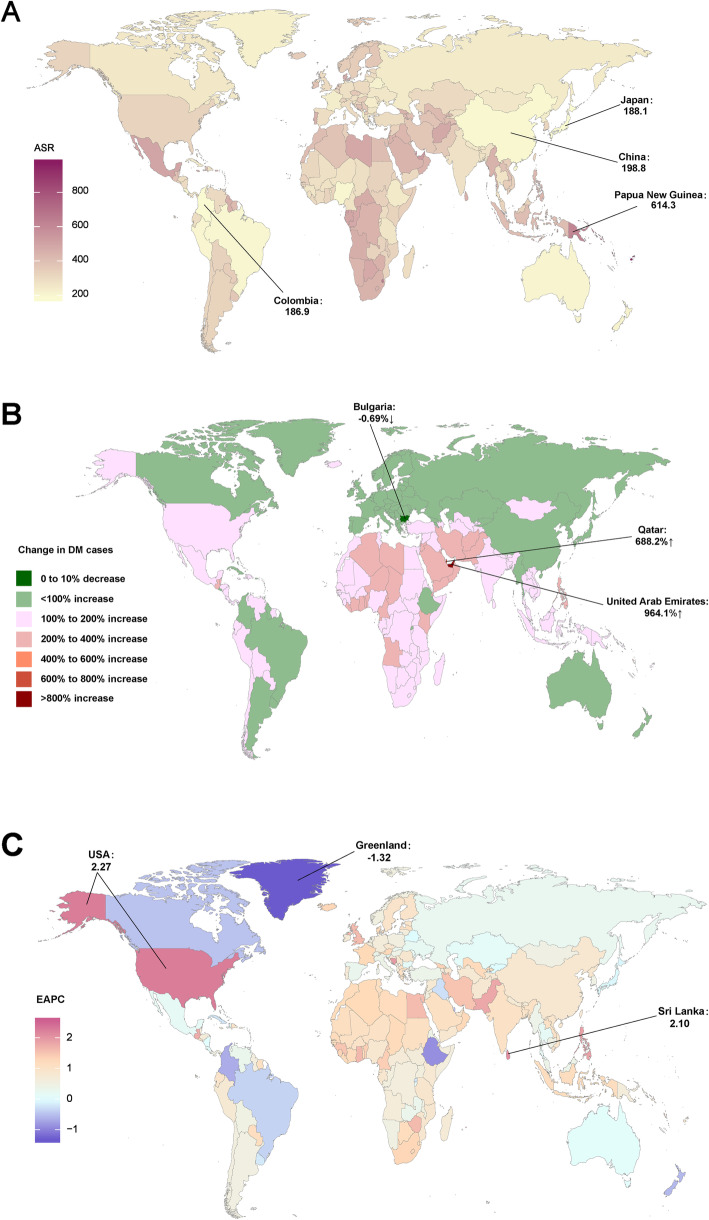
The global disease burden of diabetes mellitus for both sexes in 194 countries and territories. (**a**) The ASIR of diabetes mellitus in 2017; (**b**) The relative change in incident cases of diabetes mellitus between 1990 and 2017; (**c**) The EAPC of diabetes mellitus ASIR from 1990 to 2017. Countries with an extreme number of cases/evolution were annotated. ASIR, age-standardized incidence rate; EAPC, estimated annual percentage change (The maps were drawn by authors according to the corresponding data)

At the national level, the incident cases of diabetes mellitus increased the most in the United Arab Emirates (964.1%) and decreased the most in Bulgaria (− 0.7%) (Fig. 3b, Table S4). In addition, as for the absolute number, the largest number of new cases in 2017, were in India (3,639,083 × 10^3^cases), followed by China (3,338,131 × 10^3^cases) and the USA (1,388,743 × 10^3^ cases) (Table S4). The ASIR of diabetes mellitus varied considerably across the world in 2017, being highest in Kiribati (970 /100,000 persons), followed by Fiji and American Samoa (these countries are not marked on the map in the figure), and lowest in Colombia (187/100,000 persons), followed Japan and China (Fig. 3a, Table S4). The increase in ASIR was largest in Mauritius (EAPC = 2.56, 95% CI:2.32–2.81), followed by Sri Lanka and the USA and the decrease in ASIR was largest in Greenland (EAPC = -1.32, 95% CI: from-1.38 to − 1.26) followed by Ethiopia and Singapore from 1990 to 2017 (Fig. 3c).

**Fig. 4:**
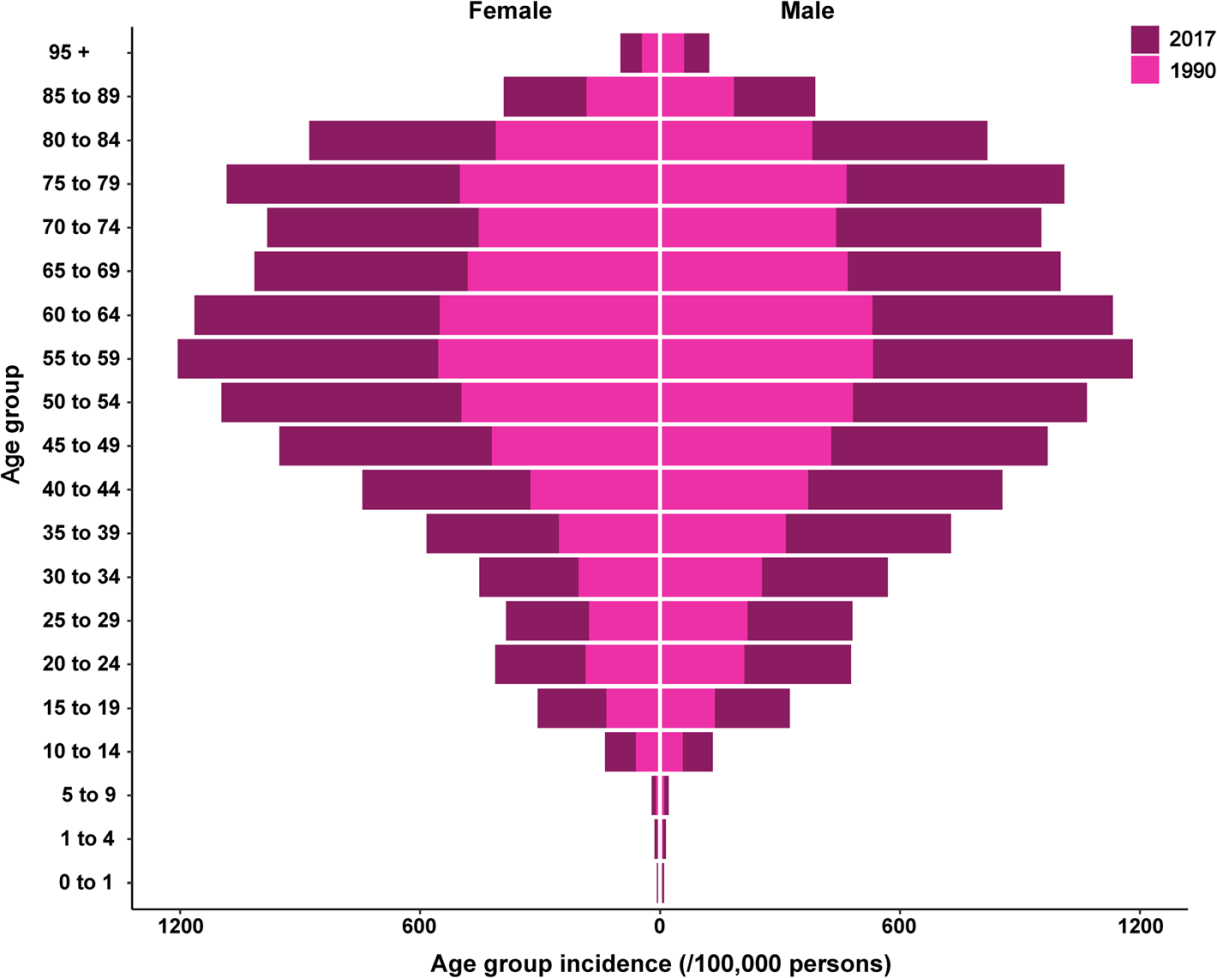
The age group incidence (per 100,000 persons) of diabetes mellitus by sex in 1990 and 2017

Examining the relationship between all age groups and incidence showed that the incidence of diabetes mellitus increased from the 0–1 age group, peaked in the 55–59 age group, after which it decreased slightly in 70–74 age group and increased in 75–79 age group and then decreased in both sexesin 1990 and 2017 (Fig. 4).

Besides, our study reported the prevalent cases and prevalence of diabetes mellitus of geographical regions. The number of diabetes mellitus patients increased in different degrees in 21 geographical regions and the prevalence of diabetes mellitus increased in 19 geographical regions (except Australasia and Tropical Latin America) from 1990 to 2017 (Table S3).

### Type 1 diabetes

T1DM accounted for nearly 1.8% (*n* = 400 × 10^3^) of the total number of diabetes mellitus incident cases in 2017, while the proportion exceeded 5.0% in Greenland. At the global level, the number of annual incident cases was rising with 291 × 10^3^ (95% UI, 263 × 10^3^–323 × 10^3^) in 1990 and 400 × 10^3^ (95% UI, 362 × 10^3^–442 × 10^3^) in 2017 (Table S1, Fig. S3A). The global ASIR of T1DM displayed an increasing trend with an EAPC of 0.34 (95% CI: 0.30–0.39) from 1990 to 2017 (Table S1).

The absolute incident case numbers in males was observed with 160 × 10^3^ (95% UI, 145 × 10^3^–177 × 10^3^) in 1990 and 211 × 10^3^ (95% UI, 200 × 10^3^–244 × 10^3^) in 2017 (The number of cases has increased in 1990–2017 except 1994, Fig. S3A), while in females from 131 × 10^3^ (95% UI, 118 × 10^3^–145 × 10^3^) to 179 (95% UI, 162 × 10^3^–198 × 10^3^) (Table S1). The ASIR of T1DM increased from 1990 to 2017 and the ASIR increased by annually an average of 0.34 (0.30–0.39) in males and females (Table S1).

**Fig. 5:**
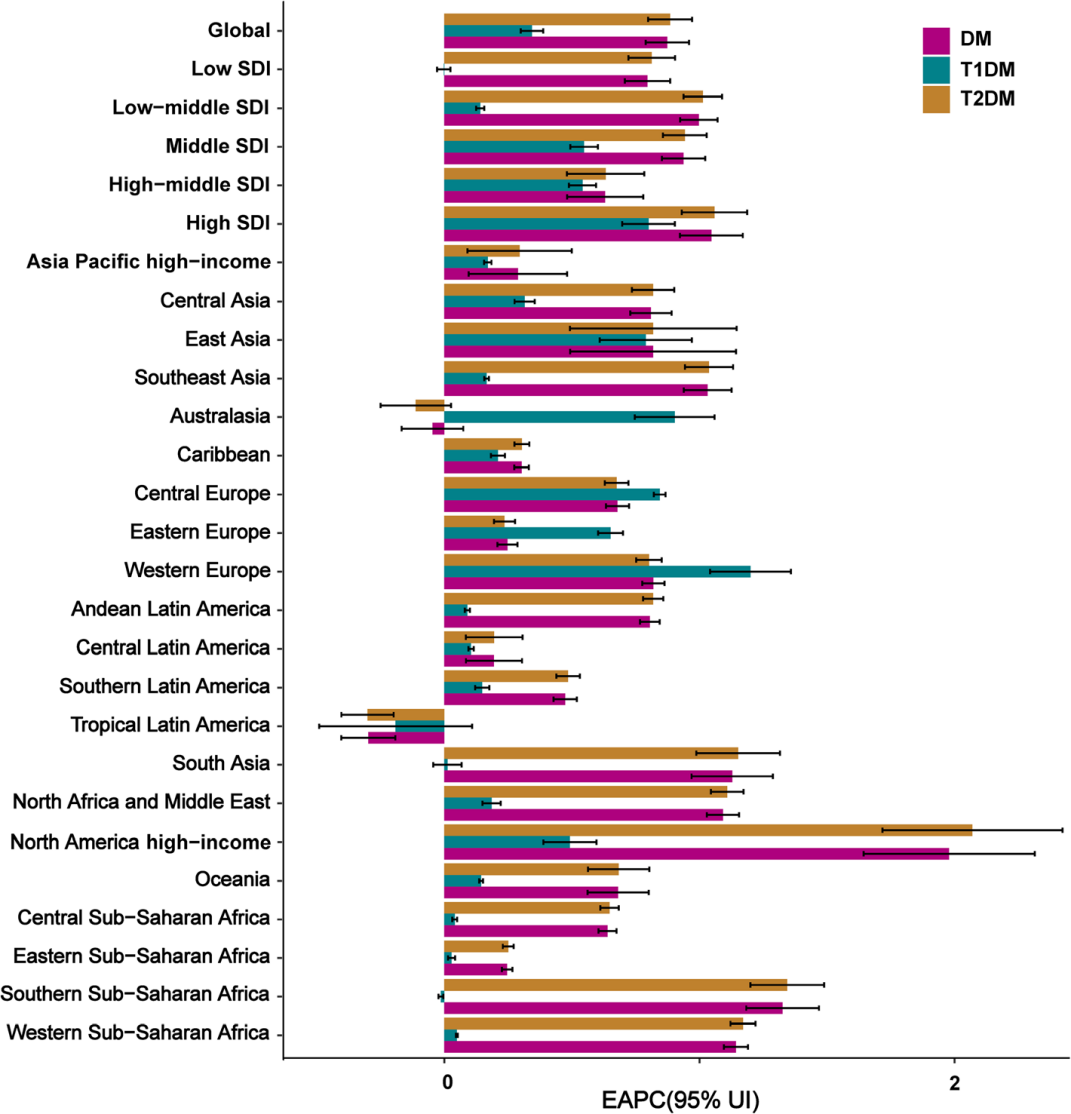
The EAPCs of diabetes mellitus (type 1 diabetes and type 2 diabetes) ASIR from 1990 to 2017 at global, regional, and national level

At the regional level, the incident cases of T1DM increased across the five SDI regions from 1990 to 2017 (Fig. S4A). The ASIR of T1DM increased across four SDI regions from 1990 to 2017 among the largest increase in high SDI (Fig. S5A), while the ASIR was stable in low-SDI regions (EAPC = 0.00, 95% CI: − 0.03–0.02) (Table S1). At the geographical level, the number of T1DM incidence cases increased in 18 geographical regions (Fig. S6A), with the increase being highest in western Sub-Saharan Africa (129.6%), followed by Central Sub-Saharan Africa (123.2%). The number of T1DM cases decreased in three regions: Asia Pacific high-income (− 21.6%), Central Europe (− 2.1%), and East Asia (− 0.2%). The largest increase in ASIR was observed in Western Europe (EAPC = 1.20, 95% CI:1.04–1.36), followed by Australasia and central Europe (Table S1, Fig. 5, Fig. S7A).

At the national level, the largest increase in the incident cases of T1DM was observed in Qatar (493.5%) followed by the United Arab Emirates (382.0%) and Afghanistan (257.6%) (Fig. S1B). Meanwhile, the largest decrease was found in Georgia (− 31.9%), followed by Bosnia and Herzegovina (− 26.6%) (Fig. S1B, Table S4). The ASIR of T1DM was highest in Norway (20/100,000 persons), followed by Canada and Uruguay and lowest in Vietnam (2/100,000 persons) in 2017 (Fig. S1A, Table S4). The ASIR in the country with the highest rate (Norway) was 10 times higher than the lowest rate (Vietnam). The largest increase in ASIR of T1DM was observed in France (EAPC = 2.11, 95% CI:1.93–2.29), and the largest decrease in ASIR was found in Finland (EAPC = − 0.72, 95% CI: from − 0.44 to − 1.00) (Fig. S1C, Table S4).

Relationship between all age groups and incidence revealed that the incidence of T1DM increased from the 0–1 age group to the 5–9 age group, peaked in the 5–9 age group, and decreased to the lowest values in the 60–64 age group, after which it slightly increased in both sexes in 1990 and 2017 (Fig. S8A).

The number of T1DM patients increased in different degrees in 21 geographical regions and the prevalence of T1DM increased in 19 geographical regions (except South Asia and Tropical Latin America) from 1990 to 2017 (Table S3).

### Type 2 diabetes

T2DM accounted for 98.3% (22,535 × 10^3^) of the total number of diabetes mellitus incident cases in 2017. The absolute number of T2DM incident cases globally increased by 104.6%, from 11,013 × 10^3^(10,283 × 10^3^–11,811 × 10^3^) in 1990 to 22,535 × 10^3^(20,694 × 10^3^–24,627 × 10^3^) in 2017 (Table S2,, Fig. S3B). The global ASIR of T2DM displayed an increasing trend with 228 /100,000 persons (95% UI, 214–244) in 1990 and 279/100,000 person (95% UI, 257–304) in 2017, with an EAPC of 0.89 (95%CI:0.80–0.97) (Table S2).

The absolute incident case numbers in males were showed with 5631 × 10^3^ (95% UI, 5247 × 10^3^–6055 × 10^3^) in 1990 and 11,549 (95% UI, 10,615 × 10^3^–12,626 × 10^3^) in 2017(The number of cases has increased in 1990–2017 except 1994, Fig. S3B), while in females from 5382 × 10^3^ (95% UI, 5028 × 10^3^–5757 × 10^3^) to 10,987 × 10^3^ (95% UI, 10,067 × 10^3^–12,037 × 10^3^) (Table S2). The ASIR of T2DM increased in 1990–2017 and the ASIR increased by annually an average of 0.91(0.82–1.00) in males and 0.86(0.78–0.95) in females (Table S2).

At the regional level, the incident cases and ASIR of T2DM increased across the five SDI regions from 1990 to 2017 (Fig. S4B, Fig. S5B). The increase in ASIR of T2DM was largest in high-SDI regions (EAPC = 1.06, 95% CI:0.93–1.19), while the increase was smallest in middle-high SDI regions in 1990–2017 (EAPC = 0.63, 95% CI: 0.48–0.78) (Table S2, Fig. S5B).

At the geographical level, the number of T2DM cases increased in all 21 regions (Fig. S6B), with the highest increase observed in western Sub-Saharan Africa (207.4%), followed by North Africa and the Middle East (199.3%), and the smallest increase was found in Eastern Europe (14.3%). The increase in ASIR was largest in North America high-income (EAPC = 2.07, 95% CI:1.72–2.42), followed by southern and western Sub-Saharan Africa and the largest decrease in ASIR was found in tropical Latin America (EAPC = − 0.30, 95% CI: − 0.40– − 0.20) (Fig. 5, Table S2, Fig. S7B).

In the 194 countries in 2017, 36% of the incident cases of T2DM occurred in India, China, and the USA, while 10% of them occurred in Indonesia, Mexico, and Pakistan. The largest increase the incident cases of T2DM was found in the United Arab Emirates (975.8%) and the largest decrease was found in Bulgaria (− 0.4%) (Table S4, Fig. S2B). The ASIR of T2DM was highest in Kiribati (968/100,000 persons), followed by Fiji ad American Samoa. and lowest in Japan (177/100,000 persons) in 2017 (Fig. S2A, Table S4). The ASIR in the country with the highest rate (Kiribati) was nearly 6 times higher than that in the country with the lowest rate. The largest increase in ASIR was in Mauritius (EAPC = 2.57, 95%CI:2.33–2.82), followed by the USA (EAPC = 2.38, 95% CI:1.97–2.78), and the largest decrease was in Greenland (EAPC = − 1.41, 95% CI: from − 1.47 to − 1.35) (Table S4, Fig. S2C).

The study showed that the incidence of T2DM increased from the 10–14 age group to the 55–59 age group, peaked in the 55–59 age group, after which it decreased slightly in both sexes in 1990 and 2017 (Fig. S8B).

The number of T2DM patients increased in different degrees in 21 geographical regions and the prevalence of T2DM increased in 19 geographical regions (except Australasia and Tropical Latin America) from 1990 to 2017 (Table S3).

**Fig. 6:**
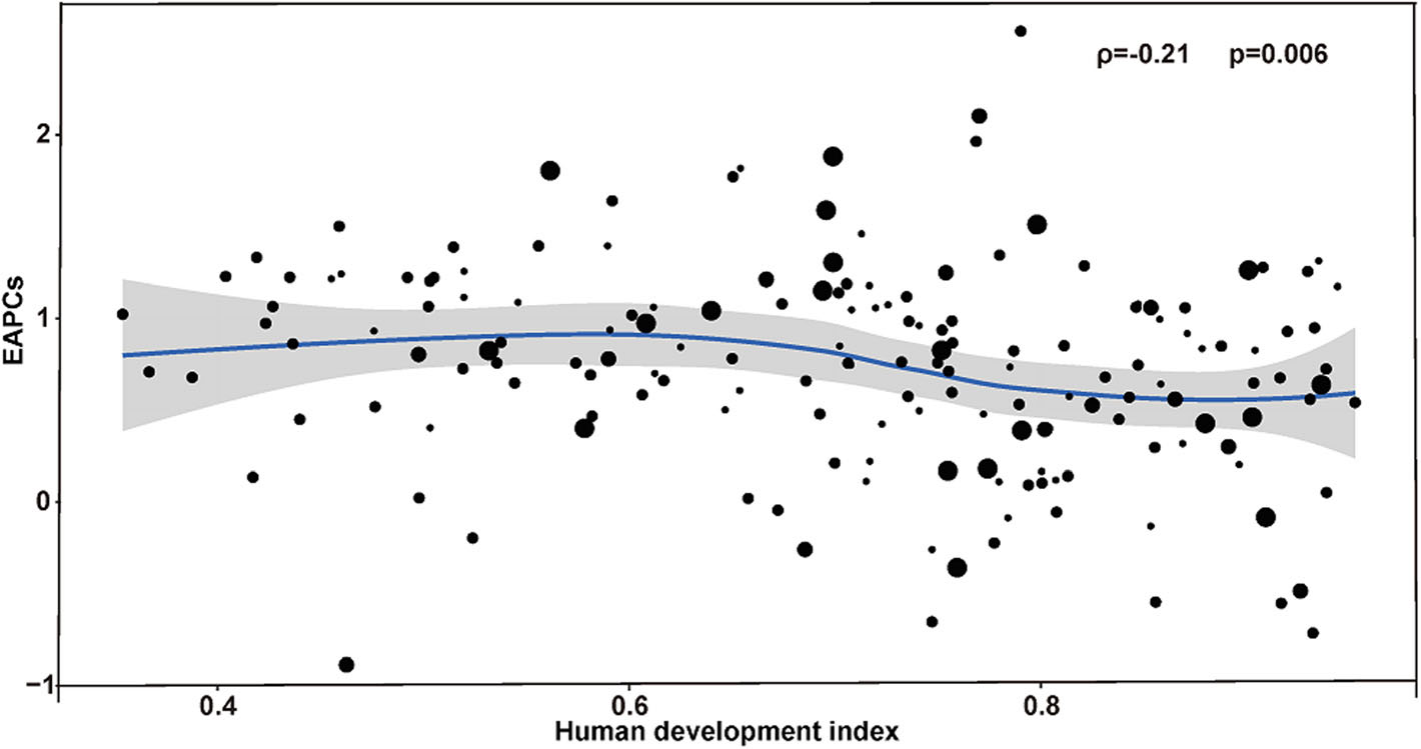
The correlation between EAPCs and human development index in 2017 at the national level. The circles represent countries that were available on HDI data. The size of the circle is increased with the incident cases of diabetes mellitus. The ρ indices and *p* values presented were derived from Pearson correlation analysis. EAPC, estimated annual percentage change; HDI, human development index

As shown in Fig. 6, a significant association was detected between EAPC and the HDI in 2017. The HDI in 2017 can serve as a surrogate for the level and availability of health care in each country, and a significant negative correlation was detected between EAPC and HDI (ρ = − 0.21, *p* = 0.006) among which EAPC was positively correlated with HDI (ρ = 0.59, *p* < 0.0001) in type 1 diabetes (Fig. S9A), and was negatively correlated with HDI (ρ = − 0.23, *p* = 0.002) in type 2 diabetes (Fig. S9B). As HDI increased, countries experienced a more-steady decrease in the ASIR of diabetes mellitus from 1990 to 2017. Besides, the study found that the ASIR of T1DM differed with latitude and the farther away from the equator, the higher the ASIR in 2017 (ρ = 0.61, *p* < 0.0001) (Fig. S10).

## Discussion

 Our study presents the most recent trends and patterns of the worldwide incidence of diabetes mellitus associated with sex, region, country, and types based on data obtained in GBD 2017. In general, both the number of incident cases and the incidence of diabetes mellitus increased from 1990 to 2017, and these trends were dominated by an increase in T2DM, with a smaller contribution from T1DM. The temporal trends in the diabetes incidence varied considerably between different regions and countries, and the heterogeneous pattern in risk-factor exposures resulted in a markedly diverse diabetes mellitus incidence across the world, which indicates the complexity of preventing diabetes mellitus [23]. This increasing pattern of diabetes mellitus was heterogeneous across regions and countries. This may be related to considerable changes in the population pyramid (age distribution) in some of these countries.

Our finding of an increasing temporal trend of incident diabetes cases over the past two decades is consistent with existing studies. The recent landmark study that performed a pooled analysis across 751 studies involving 4.4 million adults from 200 countries indicated that between 1980 and 2014 the number of adults with diabetes worldwide increased fourfold, from 108 million to 422 million [24]. The reasons for this upward trend are multiple. Known common factors, such as obesity, physical inactivity, poor dietary habits, hypertension, and dyslipidemia, have been widely reported [25–27].

In addition, other more recently discovered factors, such as intrauterine development [28], fetal undernutrition, and low birth weight may also contribute to the increasing trend. For example, intrauterine growth restriction may lead to high glucose levels in infants [29]; diabeteic risk in adulthood is influenced not only by genetic predisposition, but also by environmental factors during early life, such as fetal undernutrition [30]; low birth weight is also found to be a contributor to early T2DM onset in adulthood [31]. Given the diversified risk factors, public health prevention and control of diabetes should be customed to the individuals’ needs of the targeted population.

Our finding demonstrates that T2DM individuals account for the vast majority of people living with diabetes worldwide. In developing countries, the increasing T2DM level is largely associated with improvements in social development and living standards, resulting in excessive energy intake and reduced exercise in recent years [32]. Using the most populous developing country, China, as an example, the prevalence of T2DM in the Chinese population was 7.7% in 2000–2004, 9.3% in 2005–2009 and 10.1% in 2010–2014 from a meta-analysis of research [33]. Estimated 26.1 million (or 5.5%) adults aged 35 to 74 years in 2000–2001 and 118.5 million(or 10.9%) Chinese adults had diabetes in 2013 [34, 35]. Consistently, China’ Per Capita GDP has increased from 7078 yuan(RMB) to 46,629 yuan(RMB) during 2000–2014 from National Bureau of Statistics of China. The significant increase in the incidence of diabetes in China is closely related to its economic development. Besides, the age of T2DM onset also become younger due to changing in dietary habits and sedentary lifestyle related to economic development.

Our review of the worldwide epidemiology of T1DM revealed that its incidence varied markedly across countries and regions worldwide. The ASIR of T1DM differed with latitude and the farther away from the equator, the higher the ASIR in 2017. A particularly interesting finding in our study was that while the ASIR of T1DM decreased over the last two decades overall in Finland, its incidence increased during 1990–1999 before showing a sharp downward trend during 2000–2017. Although the Global Report on Diabetes on the WHO website indicates that preventing T1DM is made difficult by its cause being unknown and it not currently being preventable. Investigative efforts have centered on prevention, aiming to either delay or prevent disease onset [36]. Research from the USA has also shown that the burden of T1DM on the lives of adolescents can be reduced [37].

The present study found a weak negative association between EAPC and the HDI in 2017, among which EAPC was positively correlated with HDI in type 1 diabetes, and was negatively correlated with HDI in type 2 diabetes. The relationship between EAPC and HDI is different for different types of diabetes, which requires an in-depth analysis of the relationship from a macroeconomic perspective. Association of EAPC and HDI in this study requires further investigation.

This study is subject to several limitations. First, the lack of relevant epidemiological data made it impossible to include some of the important risk factors related to diabetes mellitus. Second, we were not able to model different patterns of certain risk factors, such as different amounts or types of alcohol consumption, the amount of smoking, exercise duration, different BMIs, the birthweight, the socioeconomic status, or public health and medical interventions. While quantifying the risks or causes of these other categories was beyond the scope of this study, we have provided better estimates of the factors influencing diabetes from a global perspective, and this information will be useful when designing new diabetes prevention strategies. Third, the GBD study provides a standardized approach for estimating incidence and prevalence, by geographical regions and countries and aims to use all accessible information on disease occurrence, natural history, and severity that passes a set of inclusion criteria. In this way, there are deviations in estimating different rates for regional and national data, mainly in small countries and less developed countries. In addition, when a (large) number of 95% confidence intervals are calculated, some of them will be expected to fail to contain the underlying quantity.

It is unclear how changing diagnostic criteria would have affected trends in our study. An American study [38] shows that a doubling of the incidence of diabetes during 1990–2008, and a plateauing between 2008 and 2012, which could be the 1997 change to the diagnostic criteria of diabetes the 1997 change to the diagnostic criteria of diabetes [39], which lowered FPG from 140 mg/dL or more to 126 mg/dL or more and encouraged a shift from the oral glucose tolerance test to fasting plasma glucose. The global burden of diabetes is enormous and growing, but in some countries it is on the decline or stable. Incidence of diabetes decreased significantly from 2007 to 2014 in Hong Kong Chinese [40]. Incidence of type 2 diabetes has stabilized in Scotland between 2004 and 2013 [41].

## Conclusions

In conclusion, our study indicates that diabetes mellitus remains a major public health concern globally. The worldwide increase in diabetes mellitus has largely been driven by global aging, economic growth, rapid urbanization, and nutritional transitions in different income level countries. In view of the high high disabling and comorbidities of diabetes, it is very important to develop reasonable prevention strategies. It requires the efforts and persistence of governments, organizations, communities and individuals. However, the complexity of diabetes prevention strategies and policies cannot be underestimated.

## Supplementary information

**Additional file 1.** Supplementary materials used to present other tables and figures of the Manuscript.

## Data Availability

To download the data used in these analyses, please visit the Global Health Data Exchange at http://gdx.Healthdata.org/gbd-results-tool.

## Abbreviations

TIDM: Type 1 diabetes
T2DM: Type 2 diabetes
EAPCs: Estimated annual percentage changes
ASIR: Age-standardized incidence rate
CI: Confidence interval
UI: Uncertainty Interval
SDI: Sociodemographic index
HDI: Human development index
