# Supplementary material for: Trends in the incidence of diabetes mellitus: results from the Global Burden of Disease Study 2017 and implications for diabetes mellitus prevention
Source: BMC Public Health. 2020 Sep 17;20:1415. doi: 10.1186/s12889-020-09502-x (PMC7500018; doi:10.1186/s12889-020-09502-x)
Supplement: Supplementary file 1 — Additional file 1. Supplementary materials used to present other tables and figures of the Manuscript. [file 12889_2020_9502_MOESM1_ESM.docx]

**Additional file 1: Supplementary materials used to present other tables and figures of the Manuscript.**

**Trends in** **the incidence of** **diabetes mellitus: results from the Global Burden of Disease Study 2017 and implications for diabetes mellitus prevention**

Jinli Liu, Zhen-Hu Ren, Hua Qiang, Jine Wu, Mingwang Shen, Lei Zhang, Jun Lyu

Table of contents

Fig. S1…………………………………………………………………………………………………………...2

Fig. S2…………………………………………………………………………………………………………...3

Fig. S3…………………………………………………………………………………………………………...4

Fig. S4…………………………………………………………………………………………………………...5

Fig. S5…………………………………………………………………………………………………………...6

Fig. S6…………………………………………………………………………………………………………...7

Fig. S7…………………………………………………………………………………………………………...8

Fig. S8…………………………………………………………………………………………………………...9

Fig.S9…………………………………………………………………………………………………………...10

Fig. S10…………………………………………………………………………………………………………10

Table S1. ……………………………………………………………………………………………………….11

Table S2………………………………………………………………………………………………………...12

Table S3………………………………………………………………………………………………………...13

Table S4………………………………………………………………………………………………………...15


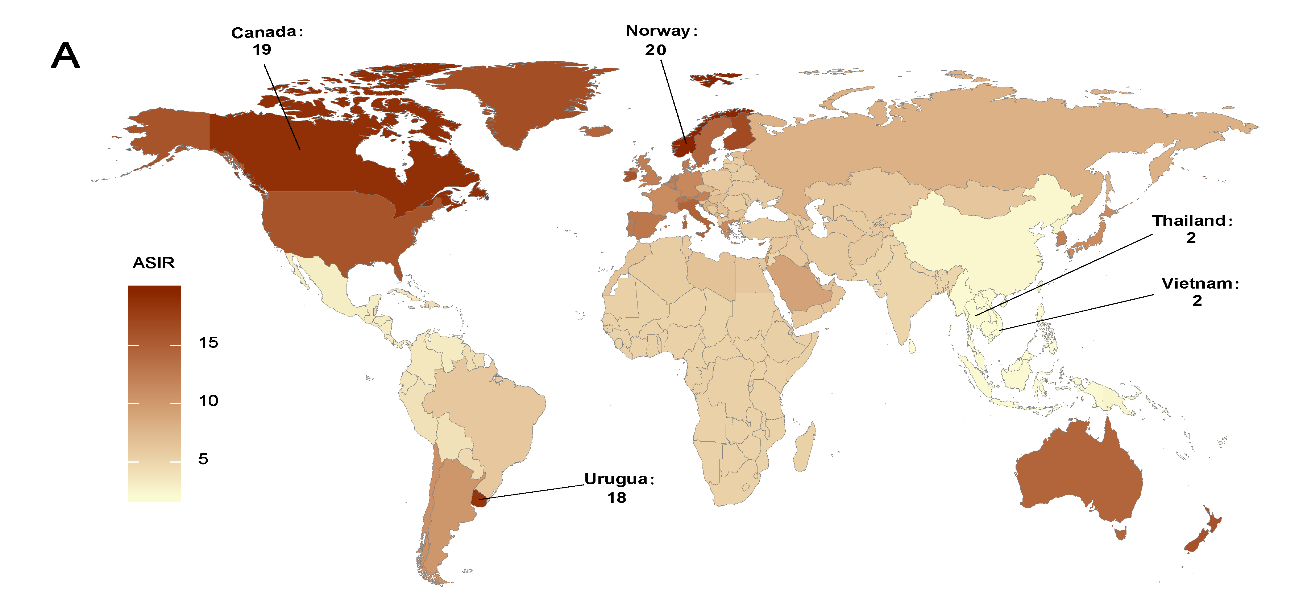


**
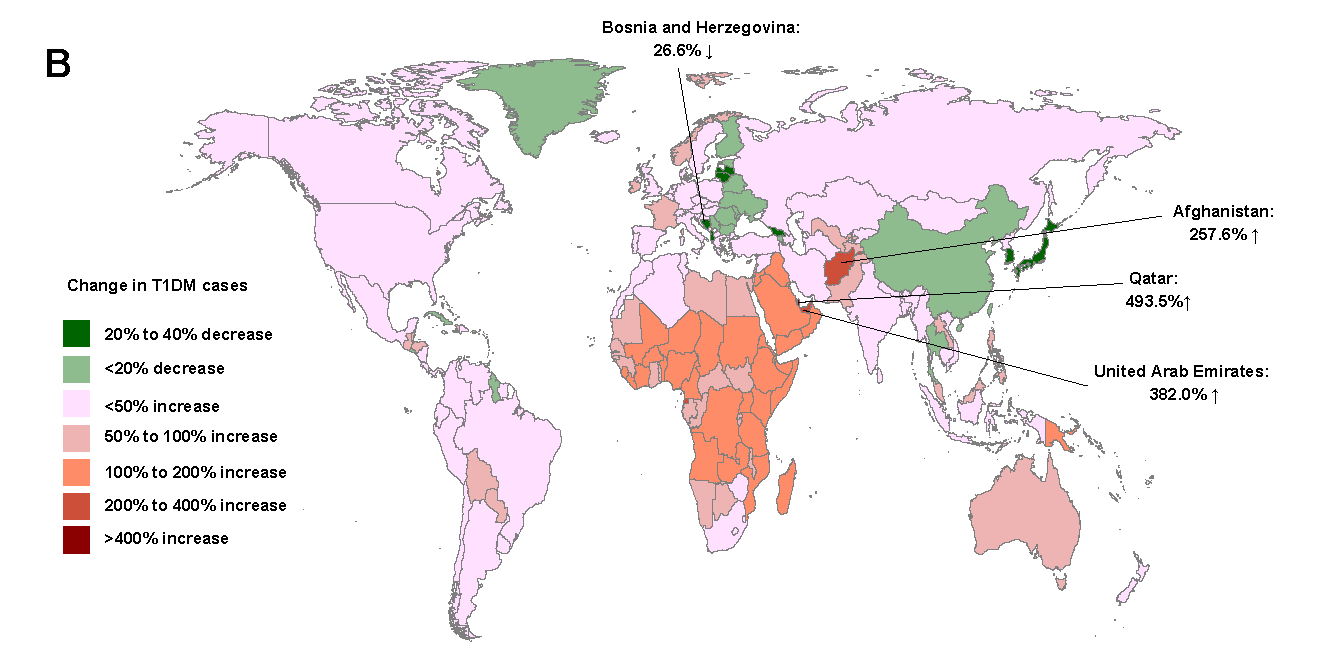
**

**
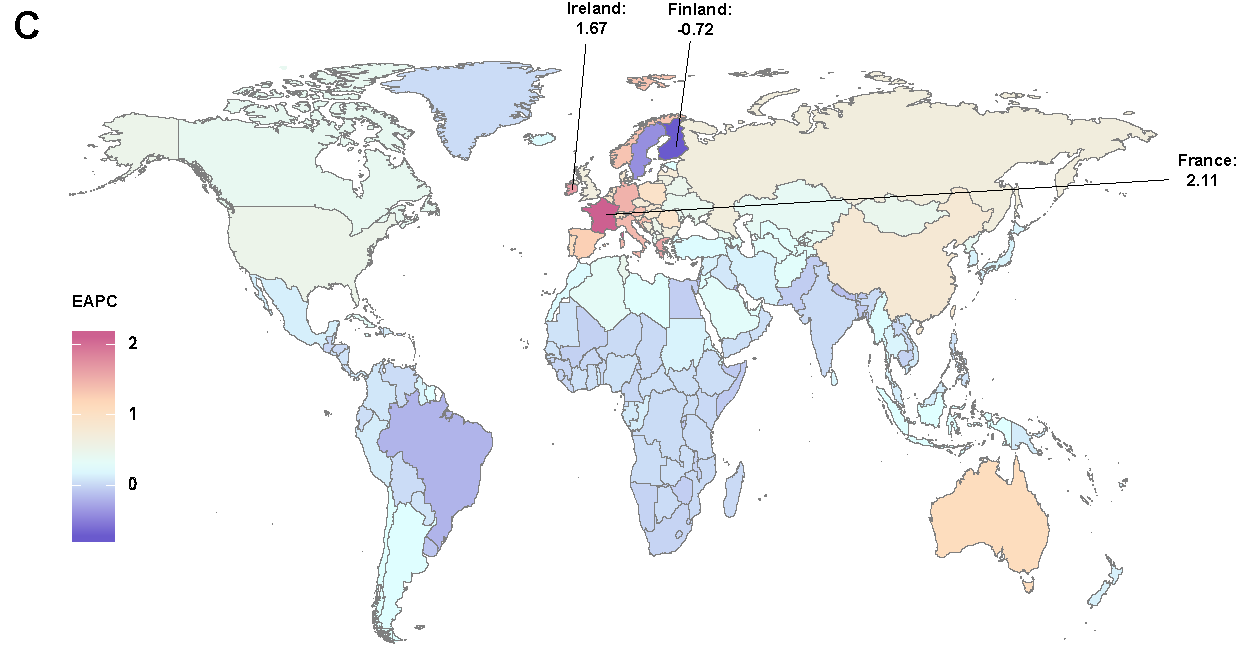
**

**Fig. S1.** The global disease burden of type 1 diabetes (T1DM) for both sexes in 194 countries and territories. (A) The ASIR of type 1 diabetes (T1DM) in 2017; (B) The relative change in incident cases of type 1 diabetes (T1DM) between 1990 and 2017; (C) The EAPC of type 1 diabetes (T1DM) ASIR from 1990 to 2017. Countries with an extreme number of cases/evolution were annotated. ASIR, age-standardized incidence rate; EAPC, estimated annual percentage change. (The maps were drawn by authors according to the corresponding data)


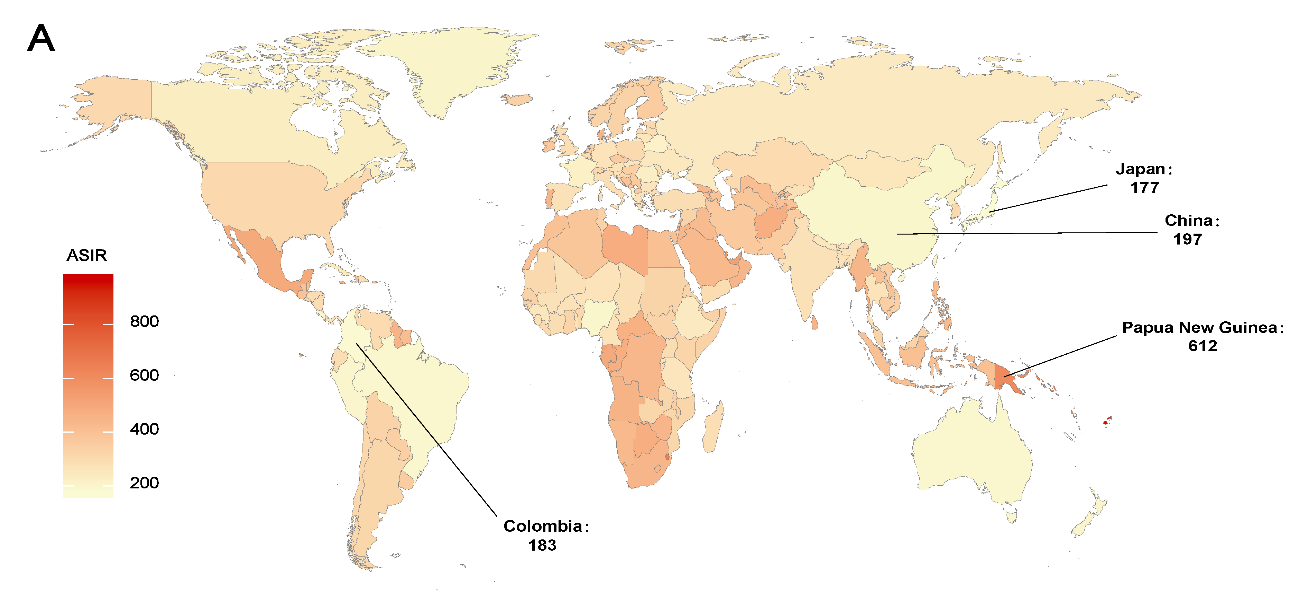


**
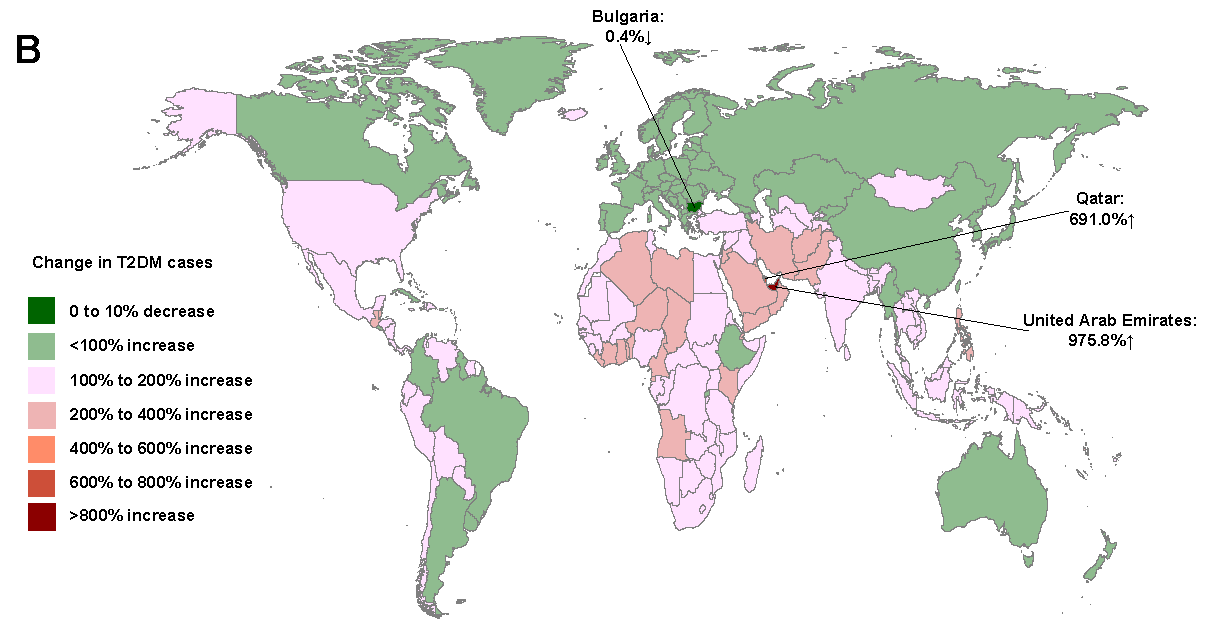

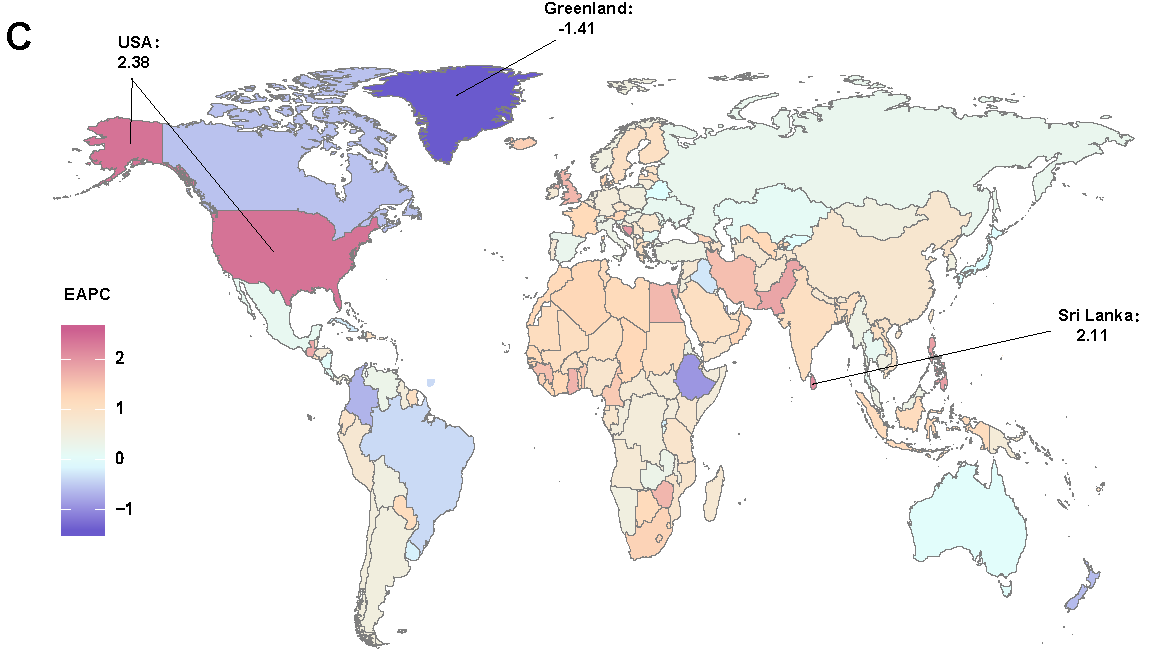
**

**Fig. S2.** The global disease burden of type 2 diabetes (T2DM) for both sexes in 194 countries and territories. (A) The ASIR of type 2 diabetes (T2DM) in 2017; (B) The relative change in incident cases of type 2 diabetes (T2DM) between 1990 and 2017; (C) The EAPC of type 2 diabetes (T2DM) ASIR from 1990 to 2017. Countries with an extreme number of cases/evolution were annotated. ASIR, age-standardized incidence rate; EAPC, estimated annual percentage change. (The maps were drawn by authors according to the corresponding data)

**
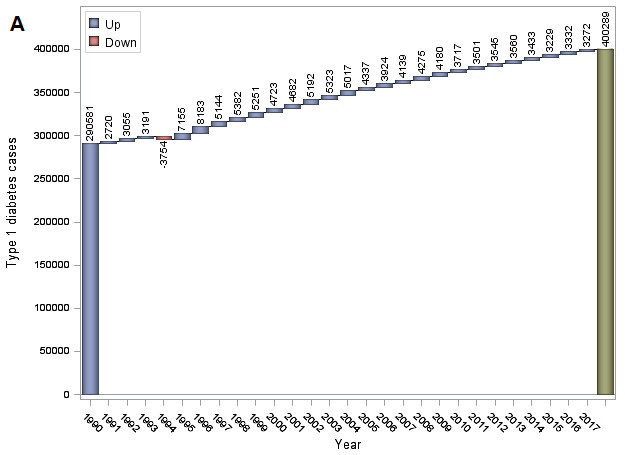
**

**
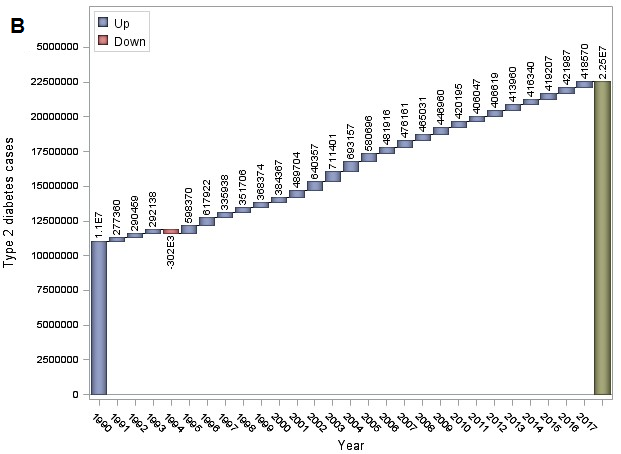
**

**Fig. S3.** The global changed incident cases of type 1 diabetes (A) and type 2 diabetes (B) for years during 1990-2017.


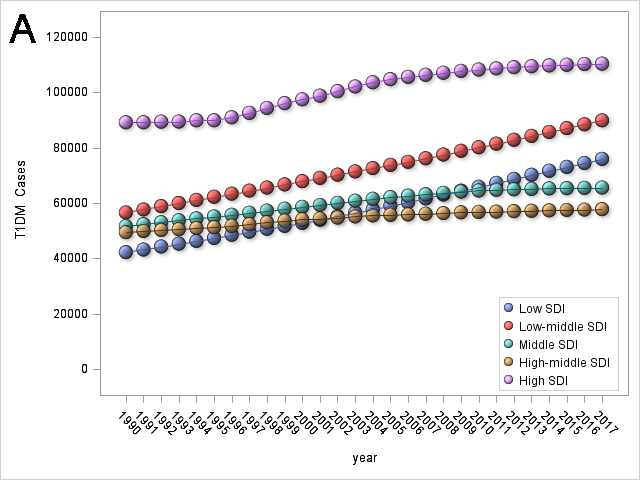


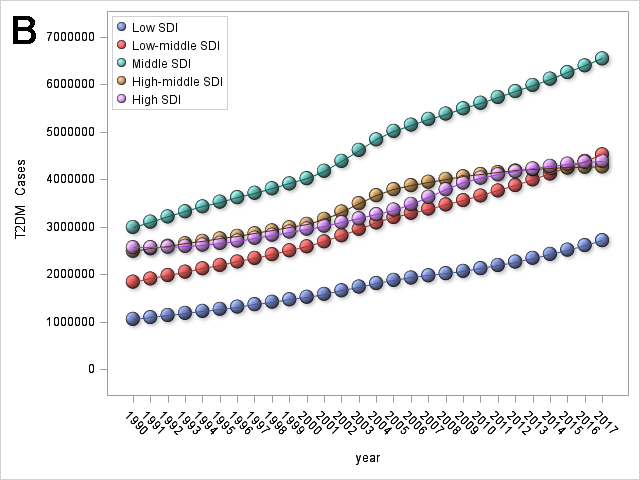


**Fig. S4.** The incident cases of diabetes mellitus (A: T1DM, B: T2DM) caused by SDI regions, from 1990 to 2017. The data from five SDI regions are presented in the top-right panel. (T1DM: type 1 diabetes; T2DM: type 2 diabetes; SDI: socio-demographic index)


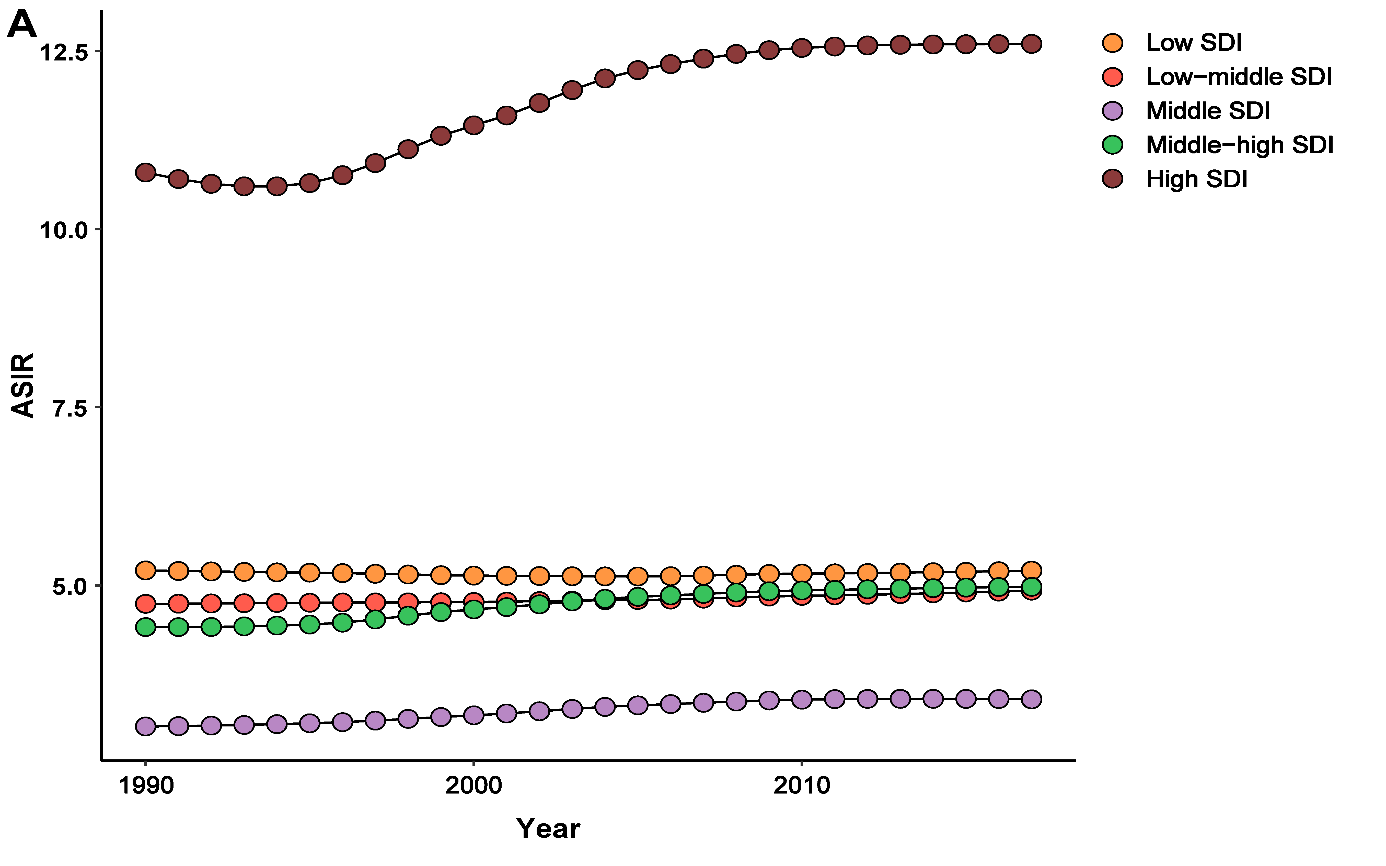


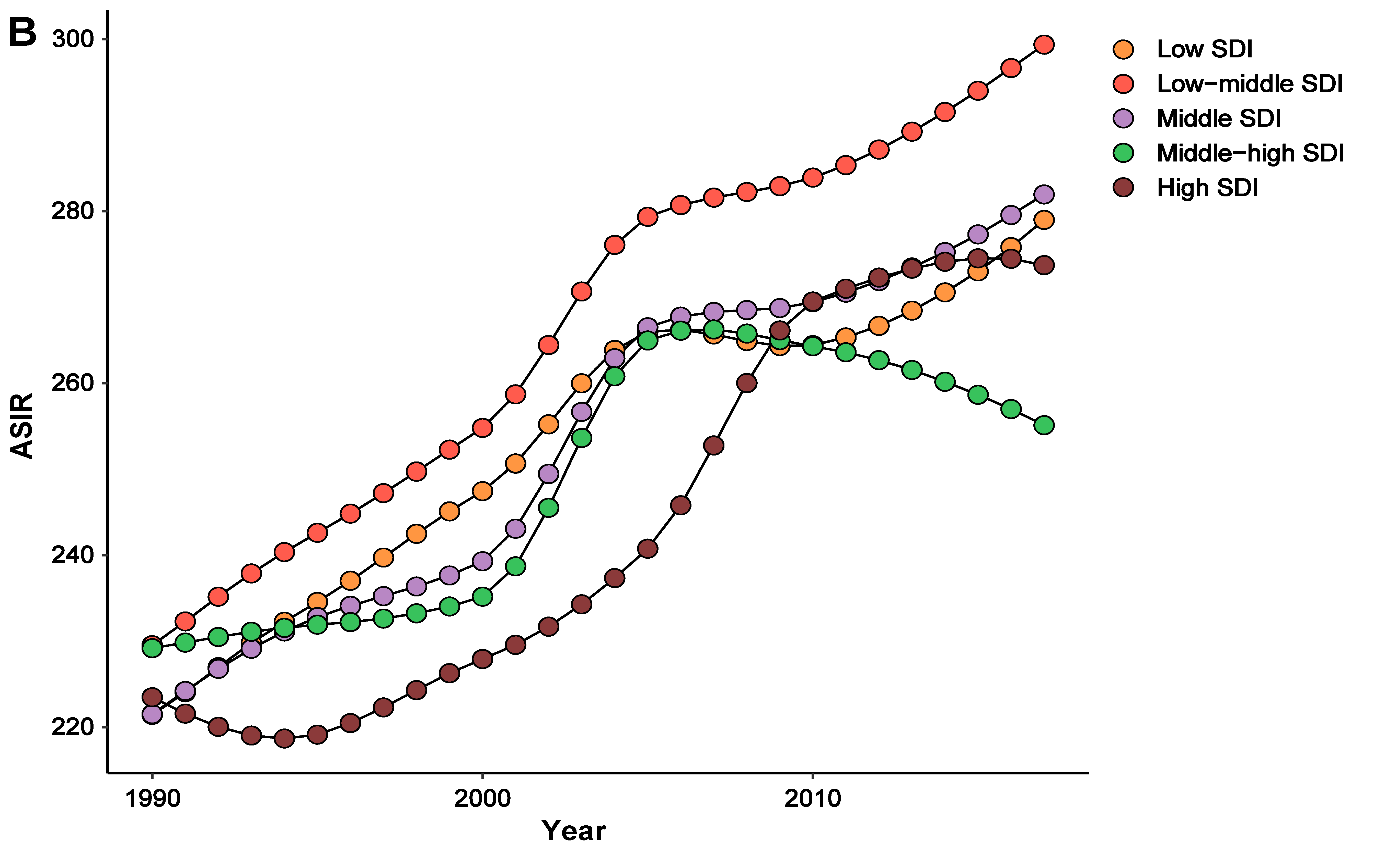


**Fig. S5.** The ASIR of diabetes mellitus (A: T1DM, B: T2DM) caused by SDI regions, from 1990 to 2017. The data from five SDI regions are presented in the top-right panel. (ASIR: age-standardized incidence rate; T1DM: type 1 diabetes; T2DM: type 2 diabetes; SDI, socio-demographic index)


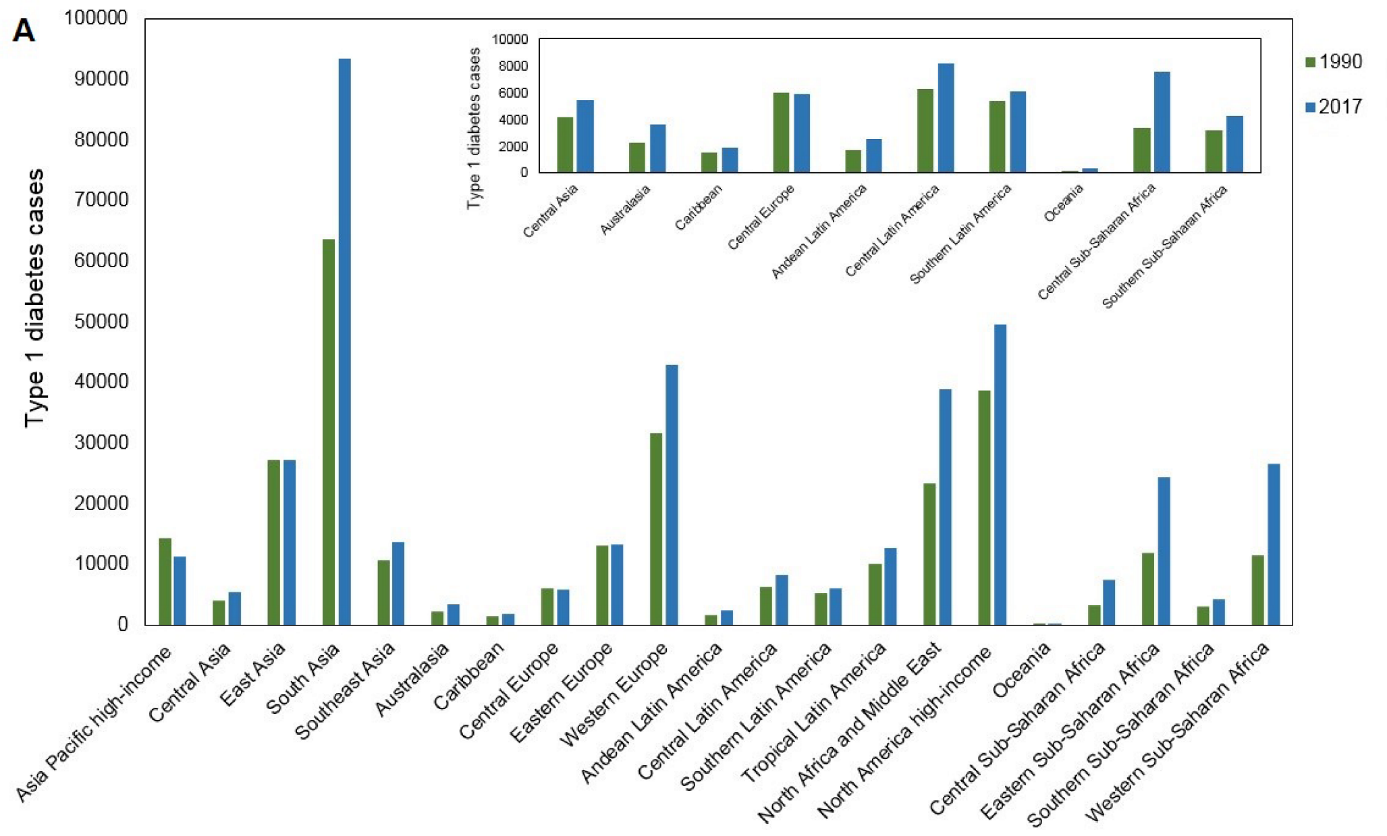


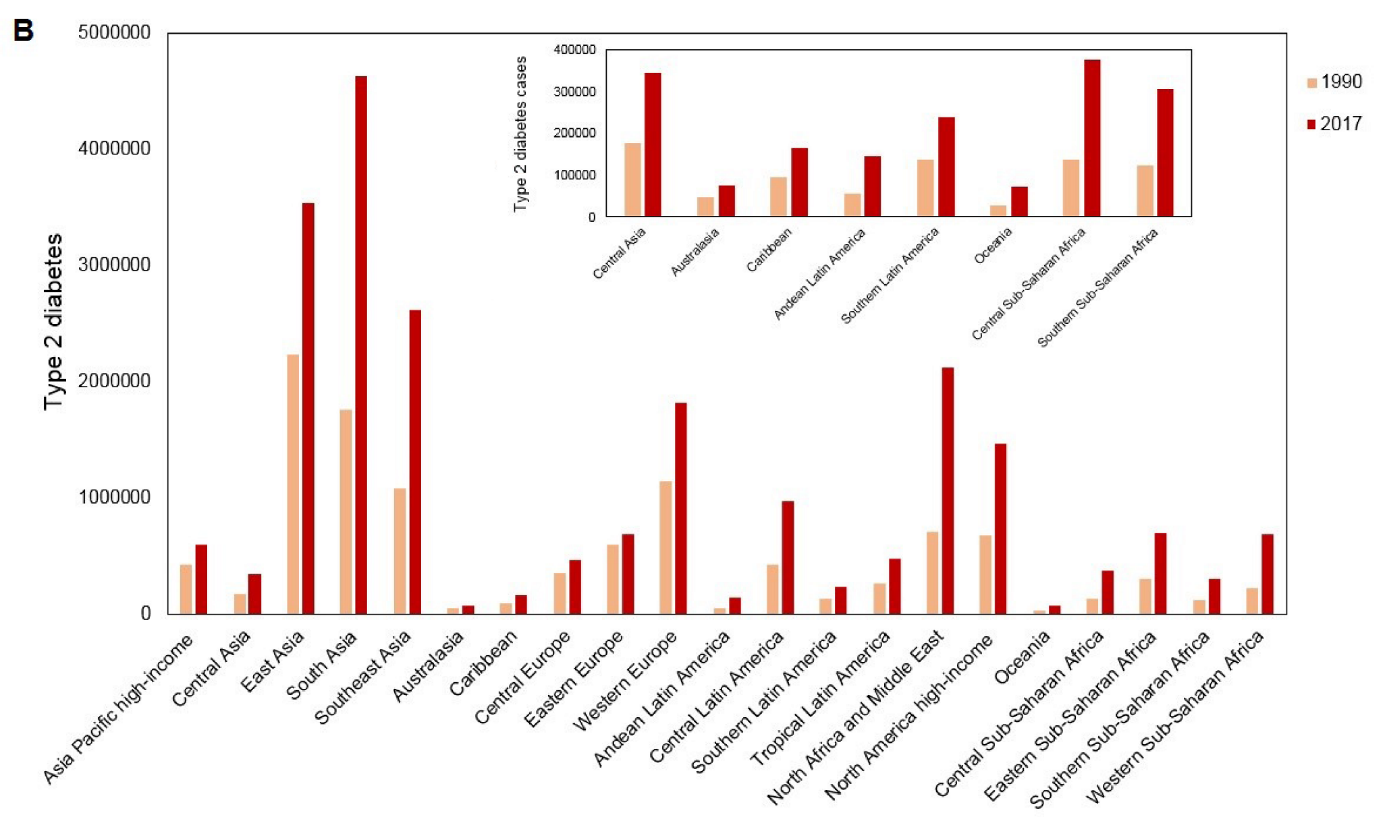


**Fig. S6.** The incident cases of type 1 diabetes (A) and type 2 diabetes (B) at a regional level. The left column in each group is case data in 1990 and the right column in 2017. Those data from certain regions can be viewed in the top-right of the panel.


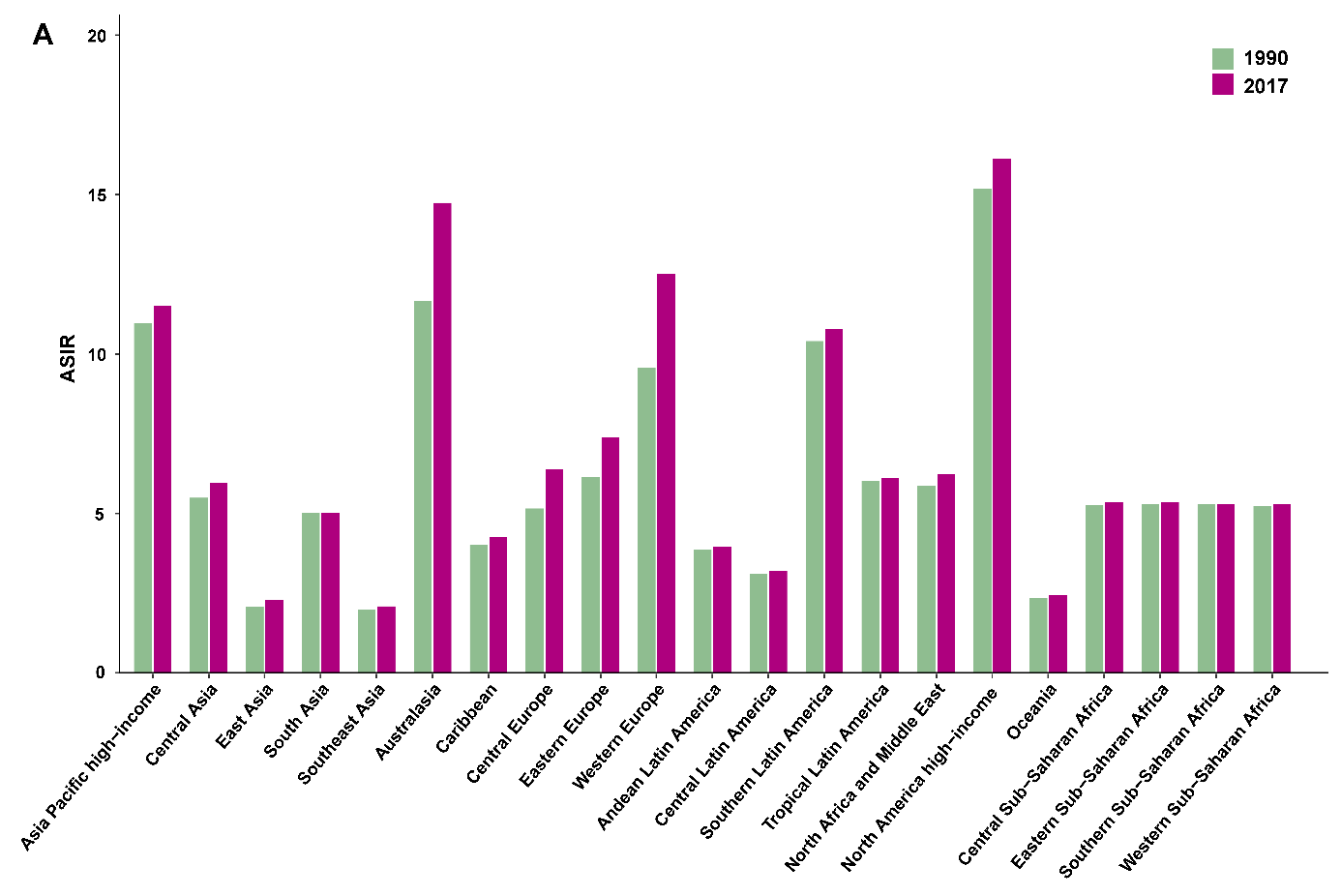


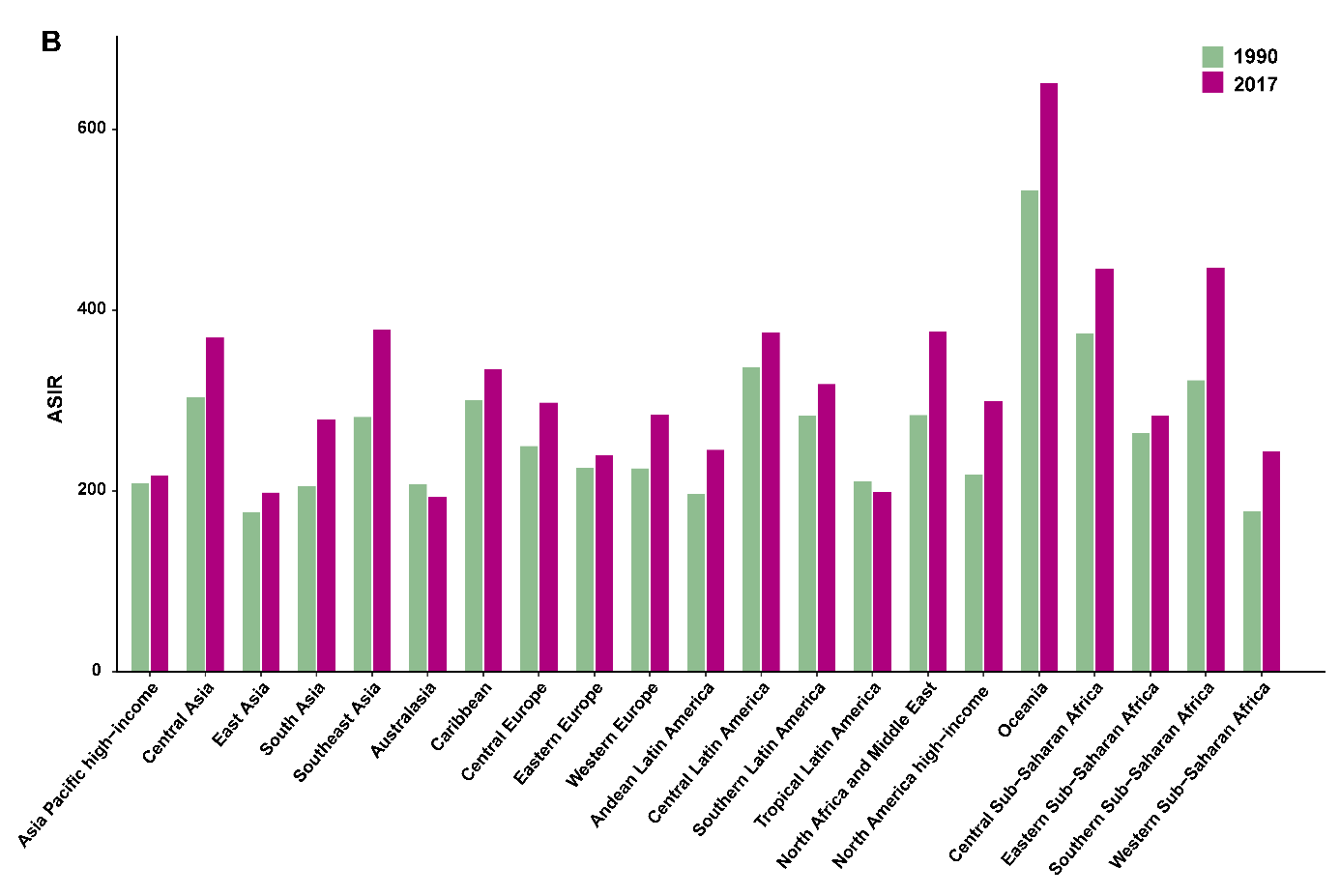
 **Fig. S7.** The ASR of diabetes mellitus (A: T1DM, B: T2DM) at a regional level. The left column in each group is case data in 1990 and the right column in 2017. Those data from certain regions can be viewed in the top-right of the panel. (ASIR: age-standardized incidence rate; T1DM: type 1 diabetes; T2DM: type 2 diabetes)


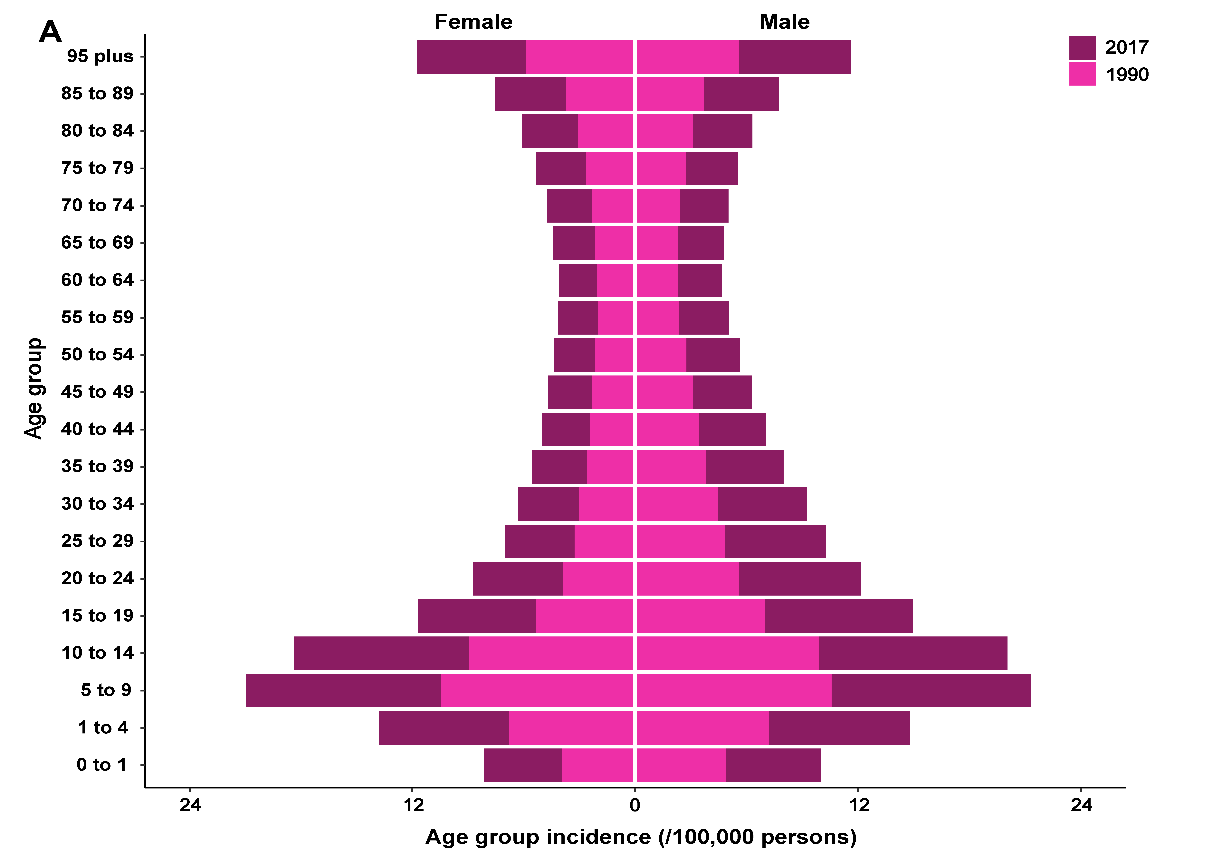


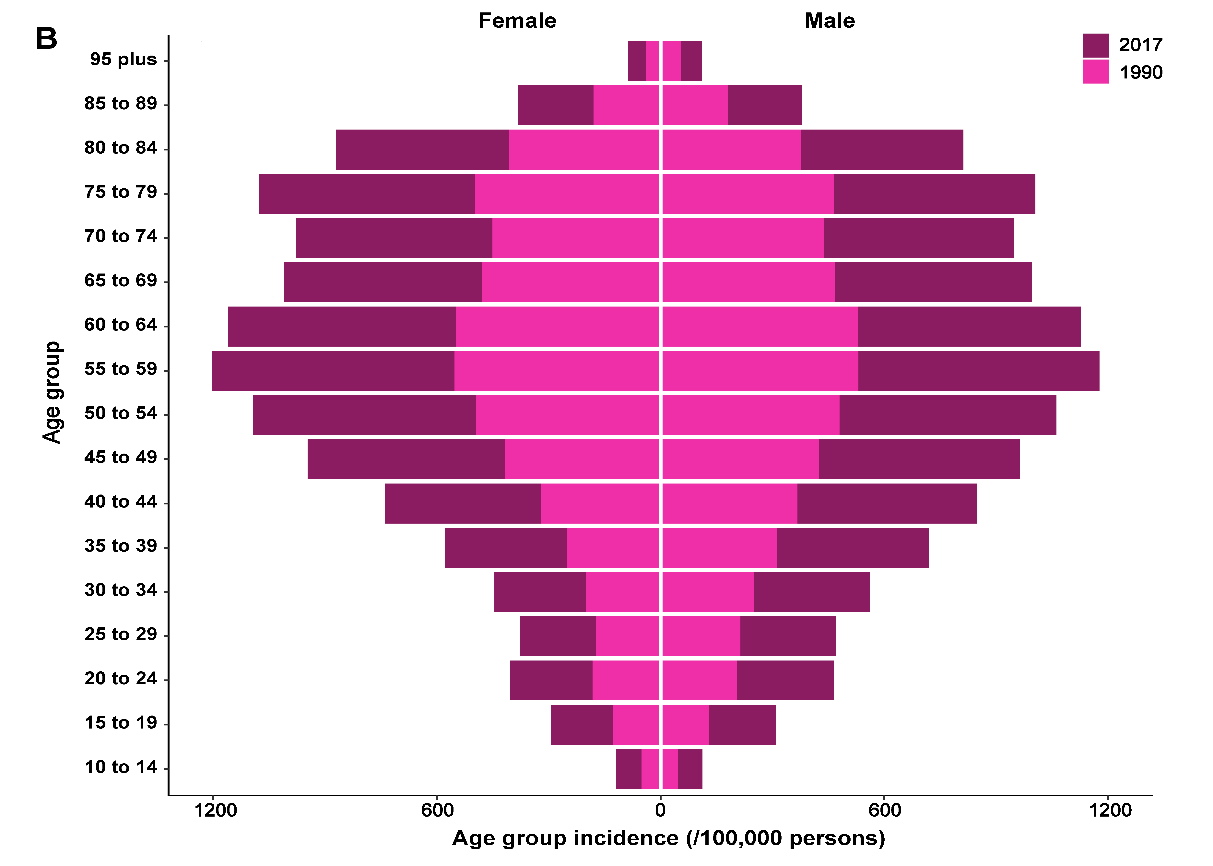


**Fig. S8.** The age group incidence (per 100,000 persons) of diabetes mellitus (A: T1DM, B: T2DM) by sex in 1990 and 2017. (T1DM: type 1 diabetes; T2DM: type 2 diabetes)


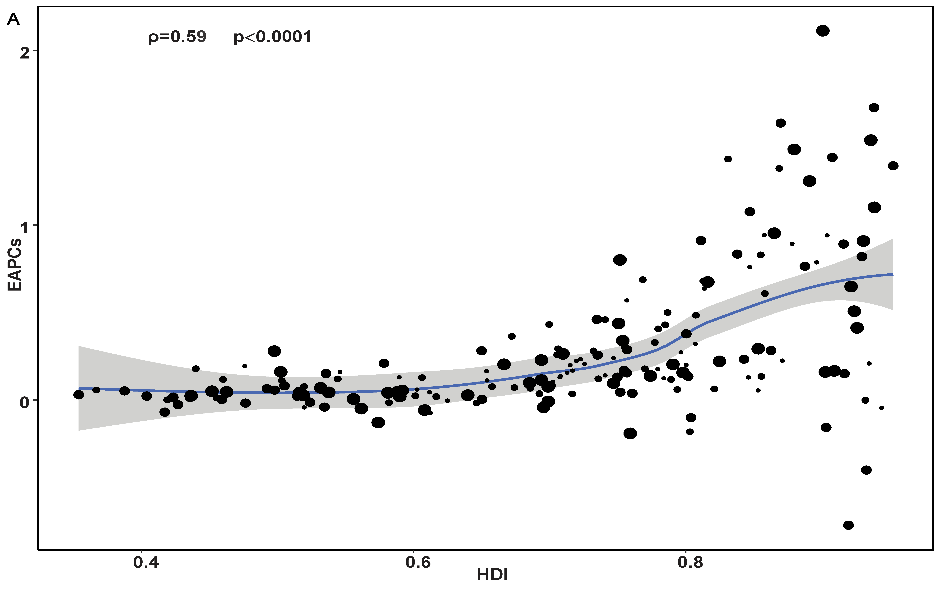


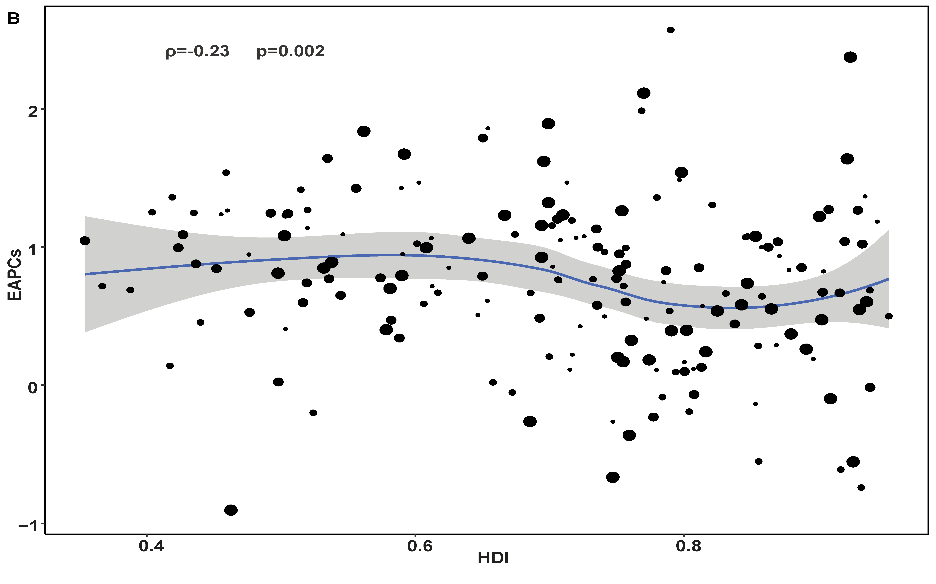


**Fig. S9.** The correlation between EAPCs and human development index in 2017 at national level. The circles represent countries that were available on HDI data. The size of the circle is increased with the incident cases of diabetes mellitus. The ρ indices and p values presented were derived from Pearson correlation analysis. (EAPC, estimated annual percentage change; HDI, human development index.

A : Type 1 diabetes , B: type 2 diabetes ) (Pearson correlation method)

**
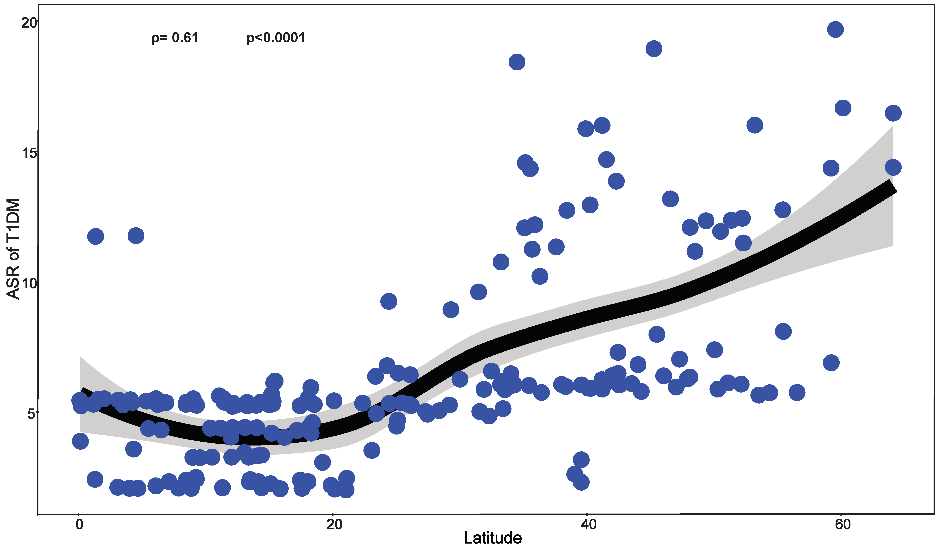
**

**Fig. S10.** The correlation between ASIR of T1DM and latitude in 2017. The ρ indices and p values presented were derived from Pearson correlation analysis. (ASIR: age-standardized incidence rate) (Pearson correlation method)Table S1. The incident cases and age-standardized incidence rate (ASIR)of type 1 diabetes in 1990 and 2017, and its temporal trends from 1990 to 2017

| Characteristics | 1990 | | 2017 | | 1990-2017 |
| --- | --- | --- | --- | --- | --- |
|  | Incident cases  No.×10^3^ (95% UI) | ASIR per 100,000  No.(95% UI) | Incident cases  No.×10^3^ (95% UI) | ASIR per 100,000  No.(95% UI) | EAPC(%)^#^  No.(95% CI) |
| Overall | 291(263–323) | 5(5-6) | 400(362–442) | 5(5–6) | 0.34(0.30–0.39) |
| Sex |  |  |  |  |  |
| Male | 160(145–177) | 5(5–6) | 221(200–244) | 6(5–7) | 0.34(0.30–0.39) |
| Female | 131(118–145) | 5(4–5) | 179(162–198) | 5(5–6) | 0.34(0.30–0.39) |
| Socio-demographic index |  |  |  |  |  |
| Low | 42(38–48) | 5(5–6) | 76(68–86) | 5(5–6) | 0.00(-0.03–0.02) |
| Low-middle | 57(50–64) | 5(4–5) | 90(81–101) | 5(4–5) | 0.14(0.12–0.16) |
| Middle | 52(46–58) | 3(3–3) | 66(59–73) | 3(3–4) | 0.55(0.49–0.60) |
| Middle-high | 49(45–55) | 4(4–5) | 58(52–64) | 5(4–6) | 0.54(0.49–0.59) |
| High | 90(83–97) | 10(10–12) | 110(101–120) | 13(12–14) | 0.80(0.70–0.90) |
| Region |  |  |  |  |  |
| Asia Pacific–high income | 14(13–16) | 11(10–12) | 11(10–12) | 12(10–13) | 0.17(0.16–0.18) |
| Central Asia | 4(4–5) | 6(5–6) | 5(5–6) | 6(5–7) | 0.31(0.28–0.35) |
| East Asia | 27(24–31) | 2(2–2) | 27 (24–30) | 2(2–3) | 0.79(0.61–0.97) |
| South Asia | 64(57–72) | 5(5–6) | 94(84–105) | 5(5–6) | 0.01(-0.04–0.07) |
| Southeast Asia | 11(9–12) | 2(2–2) | 14(12–15) | 2(2–2) | 0.17(0.16–0.17) |
| Australasia | 2(2–2) | 12(11–12) | 4(3–4) | 15(13–16) | 0.90(0.75–1.06) |
| Caribbean | 2(1–2) | 4(4–4) | 2(2–2) | 4(4–5) | 0.21(0.18–0.24) |
| Central Europe | 6(6–7) | 5(5–6) | 6(5–6) | 6(6–7) | 0.84(0.82–0.87) |
| Eastern Europe | 13(12–15) | 6(5–7) | 13(12–15) | 7(7–8) | 0.65(0.60–0.70) |
| Western Europe | 32(29–34) | 10(9–10) | 43(39–47) | 13(11–14) | 1.20(1.04–1.36) |
| Andean Latin America | 2(2–2) | 4(4–4) | 3(2–3) | 4(4–4) | 0.09(0.08–0.10) |
| Central Latin America | 6(6–7) | 3(3–4) | 8(7–9) | 3(3–4) | 0.10(0.09–0.12) |
| Southern Latin America | 5(5–6) | 10(9–12) | 6(5–7) | 11(10–12) | 0.15(0.12–0.18) |
| Tropical Latin America | 10(9–11) | 6(5–7) | 13(12–14) | 6(6–7) | -0.19(-0.49–0.11) |
| North Africa and Middle East | 23(21–26) | 6(5–7) | 39(35–44) | 6(6–7) | 0.19(0.15–0.22) |
| North America–high income | 39(35–42) | 15(14–17) | 50(45–54) | 16(15–17) | 0.49(0.39–0.60) |
| Oceania | 0.2(0.1–0.2) | 2(2–3) | 0.3(0.3–0.4) | 2(2–3) | 0.14(0.14–0.15) |
| Central Sub-Saharan Africa | 3(3–4) | 5(5–6) | 8(7–8) | 5(5–6) | 0.04(0.03–0.05) |
| Eastern Sub-Saharan Africa | 12(11–14) | 5(5–6) | 25(22–28) | 5(5–6) | 0.03(0.01–0.04) |
| Southern Sub-Saharan Africa | 3(3–4) | 5(5–6) | 4(4–5) | 5(5–6) | -0.01(-0.02–0.00) |
| Western Sub-Saharan Africa | 12(10–13) | 5(5–6) | 27(24–30 | 5(5–6) | 0.05(0.05–0.05) |

ASIR, age-standardized incidence rate; CI, confidence interval; EAPC, estimated annual percentage change; UI, uncertainty interval.

**^#^** The ASIR was deemed to be in an increasing trend if the EAPC and the lower boundary of its 95% CI were both >0%;the ASR was in a decreasing trend if the EAPC estimation and the

upper boundary of its 95% CI were both <0%; otherwise, the ASIR was deemed to be uncertain over time.

| Characteristics | 1990 | | 2017 | | 1990-2017 |
| --- | --- | --- | --- | --- | --- |
|  | Incident cases  No.×10^3^ (95% UI) | ASR per 100,000  No.(95% UI) | Incident cases  No.×10^3^ (95% UI) | ASR per 100,000  No.(95% UI) | EAPC (%)^#^  No.(95% CI) |
| Overall | 11013(10283–11811) | 228(214–244) | 22535(20694–24627) | 279(257–304) | 0.89(0.80–0.97) |
| Sex |  |  |  |  |  |
| Male | 5631(5247–6055) | 234(219–251) | 11549(10615–12626) | 289(266–315) | 0.91(0.82–1.00) |
| Female | 5382(5028–5757) | 222(208–238) | 10987(10067–12037) | 269(246–294) | 0.86(0.78–0.95) |
| Socio-demographic index |  |  |  |  |  |
| Low | 1060(979–1154) | 221(204–240) | 2720(2486–2981) | 279(254–305) | 0.81(0.72–0.90) |
| Low-middle | 1845(1703–2002) | 230(212–249) | 4528(4138–4943) | 299(273–327) | 1.01(0.94–1.09) |
| Middle | 2997(2781–32458) | 222(206–238) | 6549(5999–7184) | 282(259–308) | 0.94(0.86–1.03) |
| Middle-high | 2494(2314–26983) | 229(213–247) | 4273(3904–4712) | 255(234–279) | 0.63(0.48–0.78) |
| High | 2571(2418–27198) | 223(210–236) | 4389(4035–4802) | 274(253–297) | 1.06(0.93–1.19) |
| Region |  |  |  |  |  |
| Asia Pacific–high income | 429(397–465) | 210(195–226) | 600(537–668) | 218(197–243) | 0.29(0.09–0.50) |
| Central Asia | 177(164–191) | 305(283–327) | 345(314–381) | 370(339–407) | 0.82(0.74–0.90) |
| East Asia | 2235(2039–2452) | 178(163–195) | 3546(3217–3957) | 199(183–220) | 0.82(0.49–1.15) |
| South Asia | 1761(1615–19255) | 206(190–225) | 4631(4220–5090) | 281(255–307) | 1.15(0.99–1.32) |
| Southeast Asia | 10795(10045–1166) | 283(264–303) | 2622(2397–2875) | 380(348–415) | 1.04(0.94–1.13) |
| Australasia | 47(43–51) | 208(192–225) | 75(68–83) | 194(175–214) | -0.11(-0.25–0.03) |
| Caribbean | 95(90–100) | 302(286–319) | 166(153–181) | 335(309–365) | 0.30(0.27–0.33) |
| Central Europe | 356(332–382) | 251(235–268) | 466(423–511) | 299(273–326) | 0.68(0.63–0.72) |
| Eastern Europe | 600(552–649) | 227(209–245) | 686(617–763) | 241(219–265) | 0.24(0.19–0.28) |
| Western Europe | 1143(1062–1212) | 226(211–240) | 1819(1655–2011) | 285(261–313) | 0.80(0.75–0.85) |
| Andean Latin America | 57(53–61) | 198(186–211) | 145(133–158) | 246(226–269) | 0.82(0.78–0.86) |
| Central Latin America | 428(401–457) | 338(318–359) | 970(894–1056) | 377(348–410) | 0.20(0.08–0.31) |
| Southern Latin America | 137(127–1467) | 284(263–304) | 238(216–261) | 319(289–350) | 0.49(0.44–0.53) |
| Tropical Latin America | 262(244–281) | 211(197–227) | 482(439–530) | 200(182–219) | -0.30(-0.40– -0.20) |
| North Africa and Middle East | 710(656–771) | 285(263–309) | 2125(1942–2331) | 377(345–414) | 1.11(1.04–1.17) |
| North America high-income | 683(636–734) | 220(203–237) | 1468(1350–1589) | 300(279–324) | 2.07(1.72–2.42) |
| Oceania | 28(26–30) | 534(499–577) | 73(67–81) | 652 (602–709) | 0.68(0.56–0.80) |
| Central Sub-Saharan Africa | 136(125–148) | 375(346–407) | 377(345–415) | 447(408–489) | 0.65(0.61–0.68) |
| Eastern Sub-Saharan Africa | 302(280–327) | 265 (246–285) | 701(640–773) | 284(259–311) | 0.25(0.23–0.27) |
| Southern Sub-Saharan Africa | 124(115–135) | 324(299–351) | 307(280–336) | 448 (410–489) | 1.34(1.20–1.49) |
| Western Sub-Saharan Africa | 226(207–247) | 178(164–194) | 694(634–768) | 245(222–269) | 1.17(1.12–1.22) |

Table S2. The incident cases and age-standardized incidence rate (ASIR) of type 2 diabetes in 1990 and 2017, and its temporal trends from 1990 to 2017

ASIR, age-standardized incidence rate; CI, confidence interval; EAPC, estimated annual percentage change; UI, uncertainty interval.

**^#^** The ASIR was deemed to be in an increasing trend if the EAPC and the lower boundary of its 95% CI were both >0%; the ASIR was in a decreasing trend if the EAPC estimation and the

upper boundary of its 95% CI were both <0%; otherwise, the ASIR was deemed to be uncertain over time.

Table S3. The prevalent cases and age-standardized prevalence rate (ASPR) of diabetes mellitus (T1DM and T2DM) by geographical region in 1990 and 2017

| **Characteristics** | **1990** | | **2017** | | **1990-2017** |
| --- | --- | --- | --- | --- | --- |
|  | **Prevalent cases**  **No.×10^3^ (95% UI)** | **ASPR per 100,000**  **No.(95% UI)** | **Prevalent cases**  **No.×10^3^ (95% UI)** | **ASPR per 100,000**  **No.(95% UI)** | **Change in absolute number/ASPR (%)** |
| **DM** |  |  |  |  |  |
| Asia Pacific–high income | 8520 (7879–9249) | 4146(3833–4494) | 14489(13116–16178) | 4365(3943–4868) | 70.06/5.3 |
| Central Asia | 3356(3096–3631) | 6423(5940–6922) | 6550(5965–7251) | 7835(7161–8649) | 95.19/22.0 |
| East Asia | 44471(39970–49523) | 4102(3718–4544) | 95084(86195–105764) | 4786(4331–5321) | 113.81/16.7 |
| South Asia | 32179(29377–35278) | 4338(3981–4754) | 87168(78670–96487) | 5736(5196–6329) | 170.89/32.2 |
| Southeast Asia | 19113(17507–20865) | 5852(5395–6363) | 51964(47315–57452) | 7910(7212–8711) | 171.88/35.2 |
| Australasia | 926(8550–10030) | 3960(3662–4289) | 1632(1482–1794) | 3845(3500–4244) | 76.28/-2.9 |
| Caribbean | 1867(1745–19910) | 6414(6009–6833) | 3591(3286–3932) | 7140(6540–7814) | 92.36/11.3 |
| Central Europe | 7447(6951–8034) | 5059(4722–5458) | 11316(10366–12464) | 6161(5625–6811) | 51.96/21.8 |
| Eastern Europe | 12937(11878–14060) | 4699(4306–5102) | 15568(14114–17336) | 4924(4465–5478) | 20.34/4.8 |
| Western Europe | 23201(21621–24806) | 4352(4050–4671) | 41620(38166–45643) | 5834(5335–6440) | 79.39/34.1 |
| Andean Latin America | 1027(946–1107) | 4071(3771–4357) | 2849(2586–3135) | 5032(4571–5530) | 177.45/23.6 |
| Central Latin America | 7568(7005–8161) | 7083(6607–7596) | 19799(18179–21699) | 8001(7358–8763) | 161.62/13.0 |
| Southern Latin America | 2624(2441–2817) | 5505(5127–5910) | 4971(4529–5480) | 6355(5798–6984) | 89.45/15.4 |
| Tropical Latin America | 4656(4262–5029) | 4226(3896–4551) | 9772(8869–10852) | 4072(3697–4520) | 109.89/-3.6 |
| North Africa and Middle East | 12793(11772–13927) | 6015(5553–6539) | 39635(36120–43840) | 7901(7215–8696) | 209.82/31.4 |
| High-income North America | 14796 (13755–15899) | 4398(4080–4731) | 33446(31308–35832) | 6121(5733–6552) | 126.05/39.2 |
| Oceania | 484(443–528) | 11972(11077–12982) | 1347(1227–14867) | 15088(13788–16504) | 178.51/26.0 |
| Central Sub-Saharan Africa | 2284(2092–2512) | 7616(7020–8324) | 6365(5789–7073) | 9267(8472–10226) | 178.72/21.7 |
| Eastern Sub-Saharan Africa | 4786(4370–5238) | 4873(4489–5303) | 11375(10268–12588) | 5426(4934–5965) | 137.65/11.4 |
| Southern Sub-Saharan Africa | 2170(1983–2374) | 6375(5849–6947) | 5488(4986–6081) | 8704(7928–9603) | 152.94/36.5 |
| Western Sub-Saharan Africa | 4004(3655–4381) | 3533(3243–3852) | 11966(10852–13320) | 4918(4482–5439) | 198.86/39.2 |
| **T1DM** |  |  |  |  |  |
| Asia Pacific–high income | 700(629–778) | 370(332–412) | 783(703–874) | 391(350–435) | 11.93/5.6 |
| Central Asia | 94(84–107) | 146(130–166) | 147(132–167) | 158(141–179) | 56.78/8.2 |
| East Asia | 784(687–902) | 61(54–70) | 1105(975–1261) | 65 (57–74) | 40.91/7.0 |
| South Asia | 1417(1259–1621) | 139(124–158) | 2496(2222–2841) | 139(124–158) | 76.13/-0.2 |
| Southeast Asia | 274(241–312) | 61(54–69) | 436(388–495) | 63(56–72) | 59.49/4.8 |
| Australasia | 76(72–81) | 353(335–375) | 140(127–153) | 445(402–488) | 83.33/26.1 |
| Caribbean | 43(38–48) | 125(112–141) | 61(55–69) | 128(115–144) | 41.92/2.4 |
| Central Europe | 190(173–210) | 145(131–160) | 227(204–254) | 176(157–198) | 19.35/21.9 |
| Eastern Europe | 369(330–413) | 151(136–170) | 430(386–481) | 182(163–205) | 16.52/20.1 |
| Western Europe | 1276(1172–1389) | 299(275–326) | 1887(1704–2098) | 383(344–429) | 47.87/28.0 |
| Andean Latin America | 40(36–46) | 115(103–130) | 72(64–82) | 119(106–134) | 79.89/3.0 |
| Central Latin America | 162(144–183) | 104(92–117) | 276(245–311) | 106(94–120) | 69.73/2.4 |
| Southern Latin America | 156(141–174) | 319(288–356) | 239(212–268) | 346(307–387) | 53.28/8.3 |
| Tropical Latin America | 240(214–272) | 165(148–186) | 386(346–4366) | 164(147–185) | 61.06/-0.8 |
| North Africa and Middle East | 522(465–593) | 171(152–193) | 1095(976–12386 | 181(162–204) | 109.73/5.9 |
| High-income North America | 1359(1234–1508) | 451(409–501) | 1941(17576–21396) | 492(445–545) | 42.8/9.0 |
| Oceania | 4(4–5) | 70(63–79) | 9(8–10) | 71(63–80) | 99.31/0.8 |
| Central Sub-Saharan Africa | 67(59–76) | 142(127–161) | 156(140–178) | 144(129–163) | 134.64/1.7 |
| Eastern Sub-Saharan Africa | 220(195–251) | 139(124–157) | 481(428–549) | 140(126–159) | 119.08/1.0 |
| Southern Sub-Saharan Africa | 68(61–78) | 140(125–158) | 109(98–124) | 140(126–159) | 60.43/0.3 |
| Western Sub-Saharan Africa | 233(208–266) | 143(128–161) | 543(484–618) | 144(129–162) | 132.47/0.7 |
| **T2DM** |  |  |  |  |  |
| Asia Pacific–high income | 7820(7179–8561) | 3776(3465–4129) | 13706(12293–15355) | 3974 (3545–4476) | 75.26/5.3 |
| Central Asia | 3262(3000–3538) | 6277(5790–6772) | 6403(58253–71055) | 7677(6996–8495) | 96.3/22.3 |
| East Asia | 43687(39128–48762) | 4041(3652–4485) | 939795(849805–1047235) | 4721(4261–5265) | 115.12/16.8 |
| South Asia | 30761(27972–33863) | 4199(3841–4612) | 846715(76246–93968) | 5597(5053–6188) | 175.26/33.3 |
| Southeast Asia | 18839(17246–20571) | 5792(5339–6301) | 51528(46869–57025) | 7846(7150–8648) | 173.51/35.5 |
| Australasia | 850(780–927) | 3607(3314–3936) | 1492(1343–1659) | 3400(3046–3804) | 75.65/-5.7 |
| Caribbean | 1824(1702–1946) | 6289(5881–6706) | 3530(3225–3869) | 7012(6410–7692) | 93.55/11.5 |
| Central Europe | 7257(6762–7849) | 4914(4574–5311.38) | 11089(10124–12236) | 5984(5437–6636) | 52.82/21.8 |
| Eastern Europe | 12568(11495–13682) | 4547(4155–4943) | 15138(13704–16879) | 4742(4283–5288) | 20.45/4.3 |
| Western Europe | 21925(20344–23528) | 4052(3742–4378) | 39733(36259–43821) | 5451(4948–6060) | 81.22/34.5 |
| Andean Latin America | 987(907–1066) | 3956(3654–4242) | 2777(25147–3063) | 4914(4453–5408) | 181.43/24.2 |
| Central Latin America | 7405(6848–8002) | 6979(6508–7493 | 19523(17890–21435) | 7895(7244–8656) | 163.64/13.1 |
| Southern Latin America | 2468(2284–2660) | 5186(4803–5592) | 4732(4286–5233) | 6009(5458–6644) | 91.73/15.9 |
| Tropical Latin America | 4416(4029–4794) | 4060(3733–4388) | 9386(8471–10457) | 3908(3528–4355) | 112.54/-3.7 |
| North Africa and Middle East | 12271(11252–13406) | 5844(5386–63645) | 38540(34991–42716) | 7720(7040–8521) | 214.08/32.1 |
| High-income North America | 13437(12372–14561) | 39472(3620–4285) | 31506(29421–33948) | 5630(5242–6067) | 134.47/42.6 |
| Oceania | 479(438–523) | 11902(11000–12916) | 1338(1218–1478) | 15018(13720–16436) | 179.24/26.2 |
| Central Sub-Saharan Africa | 2217(2024–2444) | 7474(6878–8186) | 6209(5625–6924) | 9123(8333–10079) | 180.04/22.1 |
| Eastern Sub-Saharan Africa | 4567(4145–5024) | 4734(4345–5159) | 10894(9790–12104) | 5285(4791–5830) | 138.54/11.7 |
| Southern Sub-Saharan Africa | 2102(1915–2307) | 6235(5713–6807) | 5379(4876–5969) | 8564(7783–9462) | 155.93/37.4 |
| Western Sub-Saharan Africa | 3771(3422–4155) | 3390(3096–3716) | 11424(10303–12780) | 4774(4337–5299) | 202.97/40.8 |

Table S4. The change of diabetes mellitus cases between 1990 and 2017 and its age-standardized incidence rate (ASIR) in 1990 and 2017 at national level

| **Region** | **Type** | **Incident case in 1990**  **No.×10^3^ (95% UI)** | **Incident case in 2017**  **No.×10^3^ (95% UI)** | **Change**  **in absolute**  **number (%)** | **ASIR per 100,000**  **No. (95% UI)**  **in 1990** | **ASIR per 100,000**  **No. (95% UI)**  **in 2017** | **EAPCs (%)^#^**  **NO. (95CI)**  **1990-2017** |
| --- | --- | --- | --- | --- | --- | --- | --- |
| Afghanistan | Diabetes mellitus | 31133 | 105404 | 238.6 | 400 | 483 | 0.80(0.75–0.85) |
| Afghanistan | Diabetes mellitus type 1 | 683 | 2441 | 257.6 | 6 | 6 | 0.28(0.21–0.35) |
| Afghanistan | Diabetes mellitus type 2 | 30450 | 102963 | 238.1 | 394 | 477 | 0.81(0.76–0.86) |
| Albania | Diabetes mellitus | 4658 | 7125 | 53.0 | 170 | 208 | 0.73(0.67–0.8) |
| Albania | Diabetes mellitus type 1 | 202 | 149 | -26.3 | 6 | 6 | 0.43(0.37–0.48) |
| Albania | Diabetes mellitus type 2 | 4456 | 6976 | 56.6 | 164 | 202 | 0.74(0.68–0.81) |
| Algeria | Diabetes mellitus | 50841 | 154765 | 204.4 | 284 | 387 | 1.25(1.11–1.39) |
| Algeria | Diabetes mellitus type 1 | 1621 | 2278 | 40.5 | 5 | 6 | 0.34(0.3–0.38) |
| Algeria | Diabetes mellitus type 2 | 49220 | 152487 | 209.8 | 279 | 381 | 1.26(1.12–1.41) |
| American Samoa | Diabetes mellitus | 263 | 472 | 79.3 | 662 | 852 | 1.02(0.81–1.22) |
| American Samoa | Diabetes mellitus type 1 | 1 | 1 | 11.0 | 2 | 2 | 0.19(0.14–0.24) |
| American Samoa | Diabetes mellitus type 2 | 262 | 471 | 79.6 | 660 | 850 | 1.02(0.82–1.22) |
| Andorra | Diabetes mellitus | 129 | 306 | 138.0 | 218 | 286 | 1.00(0.98–1.01) |
| Andorra | Diabetes mellitus type 1 | 5 | 9 | 69.7 | 11 | 14 | 0.94(0.89–0.99) |
| Andorra | Diabetes mellitus type 2 | 123 | 297 | 140.9 | 207 | 272 | 1.00(0.98–1.01) |
| Angola | Diabetes mellitus | 26000 | 87293 | 235.7 | 386 | 464 | 0.69(0.64–0.74) |
| Angola | Diabetes mellitus type 1 | 626 | 1768 | 182.4 | 5 | 5 | 0.04(0.03–0.05) |
| Angola | Diabetes mellitus type 2 | 25374 | 85525 | 237.1 | 381 | 459 | 0.70(0.65–0.75) |
| Antigua | Diabetes mellitus | 206 | 404 | 95.9 | 375 | 391 | 0.11(0.06–0.16) |
| Antigua | Diabetes mellitus type 1 | 3 | 3 | 35.2 | 4 | 4 | 0.17(0.15–0.20) |
| Antigua | Diabetes mellitus type 2 | 204 | 401 | 96.7 | 371 | 387 | 0.11(0.06–0.16) |
| Argentina | Diabetes mellitus | 93905 | 157748 | 68.0 | 286 | 328 | 0.53(0.48–0.58) |
| Argentina | Diabetes mellitus type 1 | 3367 | 3931 | 16.7 | 10 | 10 | 0.22(0.17–0.26) |
| Argentina | Diabetes mellitus type 2 | 90538 | 153817 | 69.9 | 276 | 318 | 0.54(0.49–0.59) |
| Armenia | Diabetes mellitus | 11844 | 15614 | 31.8 | 362 | 420 | 0.71(0.51–0.91) |
| Armenia | Diabetes mellitus type 1 | 190 | 159 | -16.2 | 5 | 6 | 0.30(0.24–0.36) |
| Armenia | Diabetes mellitus type 2 | 11654 | 15455 | 32.6 | 356 | 414 | 0.72(0.52–0.92) |
| Australia | Diabetes mellitus | 41731 | 66105 | 58.4 | 221 | 207 | 0.05(-0.09–0.19) |
| Australia | Diabetes mellitus type 1 | 1703 | 2940 | 72.7 | 11 | 15 | 1.10(0.92–1.28) |
| Australia | Diabetes mellitus type 2 | 40029 | 63165 | 57.8 | 210 | 193 | -0.02(-0.17–0.14) |
| Austria | Diabetes mellitus | 20957 | 36983 | 76.5 | 208 | 284 | 1.28(1.11–1.45) |
| Austria | Diabetes mellitus type 1 | 556 | 809 | 45.4 | 9 | 12 | 1.39(1.31–1.46) |
| Austria | Diabetes mellitus type 2 | 20400 | 36174 | 77.3 | 199 | 272 | 1.27(1.10–1.45) |
| Azerbaijan | Diabetes mellitus | 20738 | 46451 | 124.0 | 326 | 400 | 0.87(0.71–1.02) |
| Azerbaijan | Diabetes mellitus type 1 | 426 | 575 | 35.0 | 5 | 6 | 0.29(0.26–0.32) |
| Azerbaijan | Diabetes mellitus type 2 | 20312 | 45876 | 125.9 | 320 | 394 | 0.88(0.72–1.04) |
| Bahamas | Diabetes mellitus | 766 | 1504 | 96.3 | 338 | 360 | 0.12(0.07–0.17) |
| Bahamas | Diabetes mellitus type 1 | 12 | 16 | 37.4 | 4 | 4 | 0.32(0.28–0.36) |
| Bahamas | Diabetes mellitus type 2 | 754 | 1488 | 97.2 | 334 | 355 | 0.12(0.07–0.17) |
| Bahrain | Diabetes mellitus | 1625 | 10083 | 520.6 | 434 | 573 | 1.06(0.85–1.27) |
| Bahrain | Diabetes mellitus type 1 | 32 | 85 | 161.0 | 6 | 6 | 0.13(0.06–0.19) |
| Bahrain | Diabetes mellitus type 2 | 1592 | 9999 | 527.9 | 428 | 567 | 1.07(0.86–1.28) |
| Bangladesh | Diabetes mellitus | 152713 | 397528 | 160.3 | 204 | 274 | 0.97(0.70–1.25) |
| Bangladesh | Diabetes mellitus type 1 | 6499 | 8030 | 23.6 | 5 | 5 | -0.06(-0.07– -0.04) |
| Bangladesh | Diabetes mellitus type 2 | 146214 | 389498 | 166.4 | 199 | 269 | 1.00(0.72–1.27) |
| Barbados | Diabetes mellitus | 983 | 1543 | 57.0 | 380 | 409 | 0.17(0.10–0.23) |
| Barbados | Diabetes mellitus type 1 | 8 | 8 | -0.2 | 3 | 3 | 0.20(0.17–0.23) |
| Barbados | Diabetes mellitus type 2 | 975 | 1535 | 57.5 | 377 | 405 | 0.17(0.10–0.23) |
| Barbuda | Diabetes mellitus | 206 | 404 | 95.9 | 375 | 391 | 0.11(0.06–0.16) |
| Barbuda | Diabetes mellitus type 1 | 3 | 3 | 35.2 | 4 | 4 | 0.17(0.15–0.20) |
| Barbuda | Diabetes mellitus type 2 | 204 | 401 | 96.7 | 371 | 387 | 0.11(0.06–0.16) |
| Belarus | Diabetes mellitus | 26844 | 27813 | 3.6 | 223 | 223 | -0.05(-0.10– -0.01) |
| Belarus | Diabetes mellitus type 1 | 485 | 439 | -9.4 | 5 | 6 | 0.48(0.42–0.55) |
| Belarus | Diabetes mellitus type 2 | 26359 | 27373 | 3.9 | 218 | 217 | -0.07(-0.11– -0.02) |
| Belgium | Diabetes mellitus | 34659 | 49588 | 43.1 | 267 | 314 | 0.68(0.63–0.73) |
| Belgium | Diabetes mellitus type 1 | 791 | 1071 | 35.4 | 9 | 12 | 0.89(0.77–1.01) |
| Belgium | Diabetes mellitus type 2 | 33867 | 48517 | 43.3 | 257 | 302 | 0.67(0.62–0.71) |
| Belize | Diabetes mellitus | 446 | 1470 | 229.4 | 332 | 405 | 0.75(0.55–0.94) |
| Belize | Diabetes mellitus type 1 | 9 | 18 | 95.1 | 4 | 4 | 0.13(0.11–0.14) |
| Belize | Diabetes mellitus type 2 | 437 | 1452 | 232.3 | 328 | 401 | 0.75(0.55–0.95) |
| Benin | Diabetes mellitus | 6693 | 24363 | 264.0 | 223 | 317 | 1.39(1.31–1.46) |
| Benin | Diabetes mellitus type 1 | 299 | 715 | 138.9 | 5 | 5 | 0.02(0.01–0.03) |
| Benin | Diabetes mellitus type 2 | 6394 | 23648 | 269.9 | 218 | 312 | 1.41(1.34–1.49) |
| Bermuda | Diabetes mellitus | 178 | 242 | 36.4 | 268 | 272 | -0.02(-0.06–0.01) |
| Bermuda | Diabetes mellitus type 1 | 2 | 3 | 16.1 | 4 | 5 | 0.56(0.53–0.58) |
| Bermuda | Diabetes mellitus type 2 | 175 | 240 | 36.6 | 264 | 267 | -0.03(-0.07–0.00) |
| Bhutan | Diabetes mellitus | 915 | 2370 | 159.1 | 228 | 271 | 0.70(0.64–0.76) |
| Bhutan | Diabetes mellitus type 1 | 33 | 49 | 48.4 | 5 | 5 | -0.07(-0.09– -0.06) |
| Bhutan | Diabetes mellitus type 2 | 881 | 2321 | 163.3 | 223 | 266 | 0.72(0.65–0.78) |
| Bolivia | Diabetes mellitus | 14091 | 34229 | 142.9 | 300 | 332 | 0.48(0.41–0.55) |
| Bolivia | Diabetes mellitus type 1 | 300 | 491 | 63.7 | 4 | 4 | 0.03(0.02–0.05) |
| Bolivia | Diabetes mellitus type 2 | 13791 | 33738 | 144.6 | 296 | 328 | 0.48(0.41–0.56) |
| Bosnia and Herzegovina | Diabetes mellitus | 12568 | 19070 | 51.7 | 260 | 399 | 1.96(1.78–2.15) |
| Bosnia and Herzegovina | Diabetes mellitus type 1 | 221 | 162 | -26.6 | 5 | 6 | 0.69(0.63–0.74) |
| Bosnia and Herzegovina | Diabetes mellitus type 2 | 12347 | 18908 | 53.1 | 255 | 393 | 1.99(1.80–2.17) |
| Botswana | Diabetes mellitus | 3138 | 9162 | 192.0 | 362 | 479 | 1.18(1.11–1.25) |
| Botswana | Diabetes mellitus type 1 | 80 | 128 | 59.7 | 5 | 5 | 0.03(0.01–0.05) |
| Botswana | Diabetes mellitus type 2 | 3058 | 9034 | 195.5 | 357 | 474 | 1.19(1.12–1.26) |
| Brazil | Diabetes mellitus | 263803 | 472249 | 79.0 | 216 | 202 | -0.36(-0.47– -0.25) |
| Brazil | Diabetes mellitus type 1 | 9949 | 12419 | 24.8 | 6 | 6 | -0.19(-0.50–0.11) |
| Brazil | Diabetes mellitus type 2 | 253853 | 459830 | 81.1 | 210 | 196 | -0.36(-0.47–-0.26) |
| Brunei | Diabetes mellitus | 1054 | 2412 | 128.9 | 556 | 522 | -0.13(-0.20– -0.06) |
| Brunei | Diabetes mellitus type 1 | 35 | 42 | 19.7 | 12 | 12 | 0.05(0.05–0.06) |
| Brunei | Diabetes mellitus type 2 | 1019 | 2370 | 132.6 | 545 | 510 | -0.14(-0.21– -0.06) |
| Bulgaria | Diabetes mellitus | 26514 | 26330 | -0.7 | 238 | 263 | 0.14(0.00–0.28) |
| Bulgaria | Diabetes mellitus type 1 | 429 | 353 | -17.8 | 5 | 6 | 0.68(0.62–0.74) |
| Bulgaria | Diabetes mellitus type 2 | 26084 | 25977 | -0.4 | 233 | 257 | 0.13(-0.01–0.27) |
| Burkina Faso | Diabetes mellitus | 13158 | 37574 | 185.6 | 213 | 275 | 0.97(0.91–1.04) |
| Burkina Faso | Diabetes mellitus type 1 | 593 | 1306 | 120.4 | 5 | 5 | 0.01(0.01–0.02) |
| Burkina Faso | Diabetes mellitus type 2 | 12566 | 36268 | 188.6 | 208 | 269 | 1.00(0.93–1.06) |
| Burundi | Diabetes mellitus | 10262 | 21368 | 108.2 | 304 | 313 | 0.14(0.10–0.17) |
| Burundi | Diabetes mellitus type 1 | 345 | 688 | 99.5 | 5 | 5 | -0.07(-0.11– -0.03) |
| Burundi | Diabetes mellitus type 2 | 9916 | 20680 | 108.5 | 299 | 308 | 0.14(0.10–0.18) |
| Cambodia | Diabetes mellitus | 21433 | 51783 | 141.6 | 296 | 348 | 0.47(0.40–0.53) |
| Cambodia | Diabetes mellitus type 1 | 263 | 357 | 35.7 | 2 | 2 | -0.02(-0.05–0.01) |
| Cambodia | Diabetes mellitus type 2 | 21170 | 51426 | 142.9 | 294 | 346 | 0.47(0.41–0.53) |
| Cameroon | Diabetes mellitus | 13603 | 50579 | 271.8 | 202 | 273 | 1.39(1.24–1.55) |
| Cameroon | Diabetes mellitus type 1 | 628 | 1696 | 170.1 | 5 | 5 | 0.00(-0.01–0.02) |
| Cameroon | Diabetes mellitus type 2 | 12976 | 48883 | 276.7 | 197 | 268 | 1.42(1.27–1.58) |
| Canada | Diabetes mellitus | 86780 | 128884 | 48.5 | 282 | 252 | -0.49(-0.56– -0.41) |
| Canada | Diabetes mellitus type 1 | 4404 | 5799 | 31.7 | 18 | 19 | 0.41(0.12–0.70) |
| Canada | Diabetes mellitus type 2 | 82376 | 123085 | 49.4 | 264 | 233 | -0.56(-0.64– -0.47) |
| Cape Verde | Diabetes mellitus | 465 | 1375 | 195.9 | 174 | 276 | 1.82(1.76–1.88) |
| Cape Verde | Diabetes mellitus type 1 | 21 | 30 | 42.9 | 5 | 5 | 0.11(0.06–0.16) |
| Cape Verde | Diabetes mellitus type 2 | 444 | 1345 | 203.1 | 169 | 271 | 1.86(1.80–1.92) |
| Central African Republic | Diabetes mellitus | 7426 | 16643 | 124.1 | 394 | 470 | 0.71(0.66–0.76) |
| Central African Republic | Diabetes mellitus type 1 | 165 | 281 | 70.1 | 5 | 5 | 0.06(0.04–0.07) |
| Central African Republic | Diabetes mellitus type 2 | 7261 | 16362 | 125.4 | 389 | 464 | 0.72(0.67–0.77) |
| Chad | Diabetes mellitus | 8253 | 25620 | 210.4 | 209 | 285 | 1.23(1.16–1.30) |
| Chad | Diabetes mellitus type 1 | 366 | 953 | 160.2 | 5 | 5 | 0.02(0.01–0.03) |
| Chad | Diabetes mellitus type 2 | 7887 | 24667 | 212.8 | 204 | 280 | 1.25(1.18–1.32) |
| Chile | Diabetes mellitus | 34479 | 70782 | 105.3 | 292 | 324 | 0.57(0.49–0.65) |
| Chile | Diabetes mellitus type 1 | 1407 | 1550 | 10.2 | 10 | 11 | 0.23(0.22–0.25) |
| Chile | Diabetes mellitus type 2 | 33073 | 69232 | 109.3 | 282 | 313 | 0.58(0.50–0.66) |
| China | Diabetes mellitus | 2122060 | 3338131 | 57.3 | 177 | 199 | 0.83(0.50–1.16) |
| China | Diabetes mellitus type 1 | 25756 | 25550 | -0.8 | 2 | 2 | 0.80(0.61–0.99) |
| China | Diabetes mellitus type 2 | 2096304 | 3312582 | 58.0 | 175 | 197 | 0.83(0.49–1.16) |
| Colombia | Diabetes mellitus | 54225 | 98977 | 82.5 | 207 | 187 | -0.65(-0.85– -0.46) |
| Colombia | Diabetes mellitus type 1 | 1294 | 1736 | 34.2 | 3 | 4 | 0.09(0.08–0.11) |
| Colombia | Diabetes mellitus type 2 | 52931 | 97241 | 83.7 | 203 | 183 | -0.67(-0.86– -0.47) |
| Comoros | Diabetes mellitus | 917 | 2004 | 118.6 | 303 | 342 | 0.40(0.34–0.46) |
| Comoros | Diabetes mellitus type 1 | 29 | 43 | 49.2 | 5 | 6 | 0.17(0.15–0.18) |
| Comoros | Diabetes mellitus type 2 | 888 | 1961 | 120.8 | 298 | 336 | 0.41(0.35–0.47) |
| Costa Rica | Diabetes mellitus | 4943 | 10370 | 109.8 | 203 | 207 | 0.09(0.05–0.14) |
| Costa Rica | Diabetes mellitus type 1 | 113 | 137 | 21.0 | 3 | 3 | 0.06(0.05–0.07) |
| Costa Rica | Diabetes mellitus type 2 | 4830 | 10233 | 111.9 | 199 | 204 | 0.09(0.05–0.14) |
| Croatia | Diabetes mellitus | 16527 | 19134 | 15.8 | 271 | 318 | 0.68(0.60–0.76) |
| Croatia | Diabetes mellitus type 1 | 253 | 276 | 9.3 | 6 | 8 | 1.38(1.30–1.45) |
| Croatia | Diabetes mellitus type 2 | 16274 | 18858 | 15.9 | 265 | 310 | 0.66(0.58–0.74) |
| Cuba | Diabetes mellitus | 27375 | 35483 | 29.6 | 245 | 248 | -0.22(-0.38– -0.06) |
| Cuba | Diabetes mellitus type 1 | 330 | 303 | -8.2 | 3 | 4 | 0.33(0.25–0.40) |
| Cuba | Diabetes mellitus type 2 | 27045 | 35181 | 30.1 | 242 | 244 | -0.23(-0.39– -0.07) |
| Cyprus | Diabetes mellitus | 2804 | 5845 | 108.4 | 346 | 365 | 0.32(0.15–0.49) |
| Cyprus | Diabetes mellitus type 1 | 64 | 123 | 92.2 | 9 | 12 | 1.32(1.27–1.37) |
| Cyprus | Diabetes mellitus type 2 | 2740 | 5722 | 108.8 | 337 | 353 | 0.29(0.12–0.46) |
| Czech Republic | Diabetes mellitus | 34587 | 54219 | 56.8 | 280 | 367 | 0.85(0.67–1.03) |
| Czech Republic | Diabetes mellitus type 1 | 575 | 650 | 13.0 | 6 | 7 | 0.76(0.7–0.83) |
| Czech Republic | Diabetes mellitus type 2 | 34012 | 53570 | 57.5 | 274 | 359 | 0.85(0.67–1.03) |
| Democratic Republic of the Congo | Diabetes mellitus | 94615 | 249871 | 164.1 | 375 | 443 | 0.61(0.57–0.65) |
| Democratic Republic of the Congo | Diabetes mellitus type 1 | 2347 | 4998 | 112.9 | 5 | 5 | 0.03(0.02–0.04) |
| Democratic Republic of the Congo | Diabetes mellitus type 2 | 92268 | 244874 | 165.4 | 370 | 438 | 0.61(0.57–0.65) |
| Denmark | Diabetes mellitus | 22426 | 37408 | 66.8 | 349 | 469 | 1.25(1.12–1.39) |
| Denmark | Diabetes mellitus type 1 | 456 | 598 | 31.1 | 10 | 13 | 0.82(0.72–0.92) |
| Denmark | Diabetes mellitus type 2 | 21970 | 36809 | 67.5 | 338 | 456 | 1.27(1.13–1.41) |
| Djibouti | Diabetes mellitus | 756 | 2889 | 282.4 | 262 | 335 | 0.93(0.89–0.97) |
| Djibouti | Diabetes mellitus type 1 | 31 | 67 | 114.9 | 5 | 6 | 0.19(0.16–0.22) |
| Djibouti | Diabetes mellitus type 2 | 725 | 2823 | 289.5 | 256 | 330 | 0.95(0.91–0.98) |
| Dominica | Diabetes mellitus | 246 | 309 | 25.4 | 368 | 394 | 0.11(0.02–0.21) |
| Dominica | Diabetes mellitus type 1 | 3 | 3 | -16.3 | 4 | 4 | 0.20(0.14–0.26) |
| Dominica | Diabetes mellitus type 2 | 243 | 306 | 26.0 | 364 | 390 | 0.11(0.01–0.21) |
| Dominican Republic | Diabetes mellitus | 12373 | 29112 | 135.3 | 219 | 280 | 0.98(0.90–1.07) |
| Dominican Republic | Diabetes mellitus type 1 | 333 | 439 | 31.6 | 4 | 4 | 0.12(0.11–0.13) |
| Dominican Republic | Diabetes mellitus type 2 | 12040 | 28674 | 138.2 | 215 | 276 | 1.00(0.91–1.09) |
| Ecuador | Diabetes mellitus | 17442 | 47240 | 170.8 | 228 | 294 | 0.94(0.87–1.00) |
| Ecuador | Diabetes mellitus type 1 | 445 | 662 | 48.7 | 4 | 4 | 0.04(0.04–0.05) |
| Ecuador | Diabetes mellitus type 2 | 16997 | 46578 | 174.0 | 225 | 290 | 0.95(0.88–1.02) |
| Egypt | Diabetes mellitus | 122042 | 353628 | 189.8 | 277 | 408 | 1.59(1.52–1.66) |
| Egypt | Diabetes mellitus type 1 | 3823 | 6481 | 69.5 | 6 | 6 | -0.04(-0.07– -0.01) |
| Egypt | Diabetes mellitus type 2 | 118219 | 347146 | 193.7 | 271 | 402 | 1.62(1.55–1.69) |
| El Salvador | Diabetes mellitus | 10330 | 19630 | 90.0 | 252 | 328 | 1.08(1.00–1.16) |
| El Salvador | Diabetes mellitus type 1 | 209 | 199 | -4.6 | 3 | 3 | 0.07(0.06–0.08) |
| El Salvador | Diabetes mellitus type 2 | 10121 | 19431 | 92.0 | 249 | 325 | 1.09(1.01–1.17) |
| Equatorial Guinea | Diabetes mellitus | 1223 | 4853 | 296.9 | 420 | 528 | 0.94(0.89–0.99) |
| Equatorial Guinea | Diabetes mellitus type 1 | 26 | 84 | 229.0 | 5 | 5 | 0.04(0.03–0.05) |
| Equatorial Guinea | Diabetes mellitus type 2 | 1197 | 4770 | 298.4 | 415 | 523 | 0.95(0.9–1.00) |
| Eritrea | Diabetes mellitus | 5024 | 12914 | 157.0 | 293 | 326 | 0.45(0.41–0.48) |
| Eritrea | Diabetes mellitus type 1 | 185 | 377 | 104.2 | 5 | 6 | 0.18(0.15–0.20) |
| Eritrea | Diabetes mellitus type 2 | 4840 | 12537 | 159.0 | 288 | 321 | 0.45(0.42–0.49) |
| Estonia | Diabetes mellitus | 4694 | 5328 | 13.5 | 256 | 309 | 0.92(0.77–1.07) |
| Estonia | Diabetes mellitus type 1 | 93 | 78 | -15.6 | 6 | 7 | 0.22(0.12–0.33) |
| Estonia | Diabetes mellitus type 2 | 4601 | 5249 | 14.1 | 250 | 302 | 0.93(0.78–1.09) |
| Ethiopia | Diabetes mellitus | 93445 | 154786 | 65.6 | 306 | 243 | -0.89(-0.91– -0.86) |
| Ethiopia | Diabetes mellitus type 1 | 3244 | 6562 | 102.3 | 5 | 6 | 0.05(0.03–0.06) |
| Ethiopia | Diabetes mellitus type 2 | 90201 | 148224 | 64.3 | 301 | 237 | -0.91(-0.93– -0.88) |
| Fiji | Diabetes mellitus | 4406 | 8929 | 102.6 | 695 | 955 | 0.96(0.64–1.29) |
| Fiji | Diabetes mellitus type 1 | 19 | 21 | 11.1 | 2 | 2 | 0.14(0.12–0.15) |
| Fiji | Diabetes mellitus type 2 | 4387 | 8908 | 103.1 | 693 | 953 | 0.96(0.64–1.29) |
| Finland | Diabetes mellitus | 17878 | 28509 | 59.5 | 296 | 374 | 0.93(0.84–1.02) |
| Finland | Diabetes mellitus type 1 | 936 | 801 | -14.4 | 22 | 17 | -0.72(-1.00– -0.44) |
| Finland | Diabetes mellitus type 2 | 16943 | 27708 | 63.5 | 274 | 357 | 1.04(0.95–1.13) |
| France | Diabetes mellitus | 120984 | 214201 | 77.1 | 170 | 232 | 1.26(1.21–1.31) |
| France | Diabetes mellitus type 1 | 3515 | 5765 | 64.0 | 7 | 11 | 2.11(1.93–2.29) |
| France | Diabetes mellitus type 2 | 117469 | 208436 | 77.4 | 163 | 221 | 1.22(1.17–1.27) |
| Gabon | Diabetes mellitus | 2942 | 7103 | 141.4 | 401 | 493 | 0.85(0.79–0.91) |
| Gabon | Diabetes mellitus type 1 | 57 | 98 | 70.5 | 5 | 5 | 0.10(0.09–0.12) |
| Gabon | Diabetes mellitus type 2 | 2884 | 7005 | 142.8 | 396 | 488 | 0.86(0.79–0.92) |
| Gambia | Diabetes mellitus | 1274 | 4202 | 229.8 | 212 | 292 | 1.24(1.18–1.30) |
| Gambia | Diabetes mellitus type 1 | 60 | 132 | 118.7 | 5 | 5 | 0.12(0.11–0.12) |
| Gambia | Diabetes mellitus type 2 | 1214 | 4070 | 235.3 | 207 | 286 | 1.26(1.21–1.32) |
| Georgia | Diabetes mellitus | 18631 | 20155 | 8.2 | 308 | 444 | 1.34(1.22–1.47) |
| Georgia | Diabetes mellitus type 1 | 288 | 196 | -31.9 | 5 | 6 | 0.41(0.37–0.44) |
| Georgia | Diabetes mellitus type 2 | 18343 | 19959 | 8.8 | 303 | 438 | 1.36(1.23–1.48) |
| Germany | Diabetes mellitus | 252540 | 365258 | 44.6 | 232 | 279 | 0.64(0.52–0.76) |
| Germany | Diabetes mellitus type 1 | 4967 | 6823 | 37.4 | 8 | 11 | 1.48(1.29–1.68) |
| Germany | Diabetes mellitus type 2 | 247573 | 358436 | 44.8 | 224 | 268 | 0.60(0.47–0.74) |
| Ghana | Diabetes mellitus | 21787 | 75228 | 245.3 | 218 | 325 | 1.64(1.54–1.74) |
| Ghana | Diabetes mellitus type 1 | 916 | 1805 | 97.0 | 5 | 5 | 0.05(0.05–0.06) |
| Ghana | Diabetes mellitus type 2 | 20870 | 73423 | 251.8 | 213 | 319 | 1.67(1.57–1.78) |
| Greece | Diabetes mellitus | 29925 | 39824 | 33.1 | 223 | 277 | 1.06(0.91–1.20) |
| Greece | Diabetes mellitus type 1 | 697 | 839 | 20.3 | 8 | 11 | 1.58(1.39–1.78) |
| Greece | Diabetes mellitus type 2 | 29228 | 38985 | 33.4 | 215 | 265 | 1.04(0.89–1.19) |
| Greenland | Diabetes mellitus | 159 | 162 | 2.0 | 306 | 222 | -1.32(-1.38– -1.26) |
| Greenland | Diabetes mellitus type 1 | 9 | 8 | -6.5 | 16 | 16 | 0.03(-0.04–0.11) |
| Greenland | Diabetes mellitus type 2 | 150 | 154 | 2.5 | 290 | 205 | -1.41(-1.47– -1.35) |
| Grenada | Diabetes mellitus | 272 | 524 | 92.4 | 381 | 426 | 0.48(0.42–0.54) |
| Grenada | Diabetes mellitus type 1 | 4 | 5 | 12.1 | 4 | 4 | 0.16(0.14–0.17) |
| Grenada | Diabetes mellitus type 2 | 268 | 519 | 93.6 | 377 | 422 | 0.48(0.42–0.54) |
| Grenadines | Diabetes mellitus | 374 | 625 | 67.0 | 426 | 494 | 0.42(0.35–0.5) |
| Grenadines | Diabetes mellitus type 1 | 5 | 5 | -8.9 | 4 | 4 | 0.23(0.21–0.25) |
| Grenadines | Diabetes mellitus type 2 | 369 | 620 | 68.1 | 422 | 490 | 0.43(0.35–0.5) |
| Guam | Diabetes mellitus | 516 | 949 | 84.1 | 409 | 535 | 1.00(0.98–1.02) |
| Guam | Diabetes mellitus type 1 | 3 | 4 | 17.7 | 2 | 2 | 0.21(0.17–0.24) |
| Guam | Diabetes mellitus type 2 | 512 | 946 | 84.5 | 407 | 532 | 1.00(0.98–1.02) |
| Guatemala | Diabetes mellitus | 14455 | 57779 | 299.7 | 253 | 395 | 1.77(1.55–1.99) |
| Guatemala | Diabetes mellitus type 1 | 342 | 632 | 84.7 | 3 | 3 | 0.00(-0.01–0.02) |
| Guatemala | Diabetes mellitus type 2 | 14113 | 57147 | 304.9 | 249 | 392 | 1.79(1.57–2.01) |
| Guinea | Diabetes mellitus | 7673 | 20387 | 165.7 | 178 | 261 | 1.5(1.39–1.62) |
| Guinea | Diabetes mellitus type 1 | 366 | 721 | 97.1 | 5 | 5 | 0.00(0.00–0.01) |
| Guinea | Diabetes mellitus type 2 | 7307 | 19666 | 169.1 | 173 | 255 | 1.54(1.42–1.66) |
| Guinea-Bissau | Diabetes mellitus | 1536 | 3936 | 156.3 | 238 | 322 | 1.21(1.12–1.31) |
| Guinea-Bissau | Diabetes mellitus type 1 | 63 | 115 | 83.3 | 5 | 5 | 0.01(-0.01–0.03) |
| Guinea-Bissau | Diabetes mellitus type 2 | 1473 | 3821 | 159.4 | 233 | 316 | 1.24(1.14–1.34) |
| Guyana | Diabetes mellitus | 2414 | 3388 | 40.4 | 390 | 458 | 0.61(0.40–0.81) |
| Guyana | Diabetes mellitus type 1 | 37 | 33 | -11.0 | 4 | 4 | 0.16(0.14–0.19) |
| Guyana | Diabetes mellitus type 2 | 2377 | 3355 | 41.1 | 386 | 453 | 0.61(0.40–0.82) |
| Haiti | Diabetes mellitus | 19796 | 41287 | 108.6 | 406 | 408 | 0.02(-0.01–0.05) |
| Haiti | Diabetes mellitus type 1 | 324 | 587 | 81.4 | 4 | 5 | 0.06(0.04–0.07) |
| Haiti | Diabetes mellitus type 2 | 19473 | 40700 | 109.0 | 402 | 404 | 0.02(-0.01–0.05) |
| Honduras | Diabetes mellitus | 8133 | 23697 | 191.4 | 244 | 292 | 0.66(0.60–0.72) |
| Honduras | Diabetes mellitus type 1 | 205 | 361 | 76.2 | 3 | 3 | 0.02(0.01–0.03) |
| Honduras | Diabetes mellitus type 2 | 7928 | 23336 | 194.4 | 240 | 289 | 0.67(0.61–0.73) |
| Hungary | Diabetes mellitus | 42123 | 46528 | 10.5 | 327 | 348 | 0.45(0.34–0.56) |
| Hungary | Diabetes mellitus type 1 | 545 | 560 | 2.6 | 6 | 7 | 0.83(0.77–0.90) |
| Hungary | Diabetes mellitus type 2 | 41578 | 45968 | 10.6 | 321 | 341 | 0.44(0.33–0.55) |
| Iceland | Diabetes mellitus | 665 | 1471 | 121.2 | 249 | 351 | 1.31(1.30–1.33) |
| Iceland | Diabetes mellitus type 1 | 33 | 44 | 31.7 | 13 | 14 | 0.21(0.18–0.24) |
| Iceland | Diabetes mellitus type 2 | 632 | 1428 | 125.9 | 236 | 336 | 1.37(1.35–1.38) |
| India | Diabetes mellitus | 1452298 | 3639083 | 150.6 | 209 | 277 | 1.04(0.87–1.22) |
| India | Diabetes mellitus type 1 | 49422 | 71620 | 44.9 | 5 | 5 | 0.03(-0.04–0.09) |
| India | Diabetes mellitus type 2 | 1402876 | 3567463 | 154.3 | 204 | 272 | 1.06(0.88–1.24) |
| Indonesia | Diabetes mellitus | 457144 | 1090002 | 138.4 | 295 | 406 | 1.15(0.88–1.42) |
| Indonesia | Diabetes mellitus type 1 | 4315 | 5541 | 28.4 | 2 | 2 | 0.23(0.22–0.24) |
| Indonesia | Diabetes mellitus type 2 | 452829 | 1084461 | 139.5 | 293 | 404 | 1.16(0.89–1.43) |
| Iran | Diabetes mellitus | 95678 | 318502 | 232.9 | 237 | 370 | 1.51(1.28–1.74) |
| Iran | Diabetes mellitus type 1 | 3964 | 4696 | 18.5 | 6 | 6 | 0.16(0.11–0.20) |
| Iran | Diabetes mellitus type 2 | 91714 | 313806 | 242.2 | 231 | 364 | 1.54(1.31–1.77) |
| Iraq | Diabetes mellitus | 56219 | 151952 | 170.3 | 462 | 429 | -0.26(-0.38– -0.14) |
| Iraq | Diabetes mellitus type 1 | 1238 | 2947 | 138.0 | 6 | 6 | 0.10(0.06–0.14) |
| Iraq | Diabetes mellitus type 2 | 54981 | 149005 | 171.0 | 456 | 423 | -0.26(-0.38– -0.14) |
| Ireland | Diabetes mellitus | 11681 | 22749 | 94.8 | 305 | 388 | 0.72(0.65–0.80) |
| Ireland | Diabetes mellitus type 1 | 392 | 691 | 76.2 | 11 | 16 | 1.67(1.54–1.8) |
| Ireland | Diabetes mellitus type 2 | 11289 | 22058 | 95.4 | 294 | 372 | 0.69(0.6–0.77) |
| Israel | Diabetes mellitus | 12676 | 33225 | 162.1 | 269 | 346 | 0.65(0.39–0.91) |
| Israel | Diabetes mellitus type 1 | 484 | 831 | 71.6 | 9 | 10 | -0.16(-0.33–0.01) |
| Israel | Diabetes mellitus type 2 | 12192 | 32393 | 165.7 | 259 | 336 | 0.67(0.4–0.94) |
| Italy | Diabetes mellitus | 197082 | 289384 | 46.8 | 251 | 300 | 0.43(0.31–0.54) |
| Italy | Diabetes mellitus type 1 | 5688 | 7209 | 26.8 | 12 | 15 | 1.43(1.04–1.82) |
| Italy | Diabetes mellitus type 2 | 191394 | 282174 | 47.4 | 239 | 285 | 0.37(0.23–0.51) |
| Ivory Coast | Diabetes mellitus | 16221 | 50440 | 211.0 | 216 | 294 | 1.22(1.07–1.37) |
| Ivory Coast | Diabetes mellitus type 1 | 752 | 1519 | 101.9 | 5 | 5 | 0.06(0.05–0.08) |
| Ivory Coast | Diabetes mellitus type 2 | 15469 | 48920 | 216.3 | 211 | 288 | 1.25(1.10–1.40) |
| Jamaica | Diabetes mellitus | 6399 | 13262 | 107.2 | 336 | 450 | 0.76(0.65–0.88) |
| Jamaica | Diabetes mellitus type 1 | 108 | 117 | 8.8 | 4 | 4 | 0.28(0.24–0.32) |
| Jamaica | Diabetes mellitus type 2 | 6292 | 13145 | 108.9 | 332 | 446 | 0.77(0.65–0.88) |
| Japan | Diabetes mellitus | 304974 | 347610 | 14.0 | 196 | 188 | -0.08(-0.33–0.17) |
| Japan | Diabetes mellitus type 1 | 9195 | 7169 | -22.0 | 11 | 11 | 0.16(0.14–0.19) |
| Japan | Diabetes mellitus type 2 | 295778 | 340441 | 15.1 | 185 | 177 | -0.10(-0.37–0.17) |
| Jordan | Diabetes mellitus | 9395 | 40535 | 331.4 | 389 | 443 | 0.57(0.50–0.65) |
| Jordan | Diabetes mellitus type 1 | 232 | 604 | 160.6 | 5 | 5 | 0.26(0.20–0.31) |
| Jordan | Diabetes mellitus type 2 | 9164 | 39931 | 335.8 | 385 | 438 | 0.58(0.5–0.66) |
| Kazakhstan | Diabetes mellitus | 46262 | 57820 | 25.0 | 296 | 303 | 0.10(0.04–0.17) |
| Kazakhstan | Diabetes mellitus type 1 | 974 | 1063 | 9.1 | 6 | 6 | 0.38(0.33–0.42) |
| Kazakhstan | Diabetes mellitus type 2 | 45287 | 56757 | 25.3 | 290 | 297 | 0.10(0.03–0.16) |
| Kenya | Diabetes mellitus | 35897 | 109165 | 204.1 | 267 | 326 | 0.78(0.72–0.84) |
| Kenya | Diabetes mellitus type 1 | 1456 | 2924 | 100.8 | 5 | 5 | 0.02(0.00–0.04) |
| Kenya | Diabetes mellitus type 2 | 34441 | 106241 | 208.5 | 262 | 321 | 0.79(0.74–0.85) |
| Kiribati | Diabetes mellitus | 451 | 1054 | 133.7 | 731 | 970 | 1.06(0.93–1.20) |
| Kiribati | Diabetes mellitus type 1 | 2 | 3 | 57.6 | 2 | 2 | 0.04(0.00–0.08) |
| Kiribati | Diabetes mellitus type 2 | 449 | 1051 | 134.0 | 728 | 968 | 1.06(0.93–1.20) |
| Kuwait | Diabetes mellitus | 4873 | 17053 | 249.9 | 332 | 373 | 0.40(0.22–0.58) |
| Kuwait | Diabetes mellitus type 1 | 172 | 365 | 111.7 | 9 | 9 | -0.18(-0.37–0.00) |
| Kuwait | Diabetes mellitus type 2 | 4701 | 16688 | 255.0 | 323 | 365 | 0.41(0.23–0.60) |
| Kyrgyzstan | Diabetes mellitus | 10428 | 16737 | 60.5 | 289 | 281 | -0.04(-0.09–0.00) |
| Kyrgyzstan | Diabetes mellitus type 1 | 269 | 400 | 48.8 | 6 | 6 | 0.36(0.33–0.39) |
| Kyrgyzstan | Diabetes mellitus type 2 | 10159 | 16337 | 60.8 | 284 | 275 | -0.05(-0.10–0.00) |
| Laos | Diabetes mellitus | 9220 | 24444 | 165.1 | 301 | 392 | 1.02(0.95–1.09) |
| Laos | Diabetes mellitus type 1 | 102 | 157 | 54.1 | 2 | 2 | 0.02(0.02–0.03) |
| Laos | Diabetes mellitus type 2 | 9118 | 24287 | 166.4 | 299 | 390 | 1.02(0.95–1.10) |
| Latvia | Diabetes mellitus | 7325 | 7649 | 4.4 | 230 | 295 | 1.08(1.00–1.16) |
| Latvia | Diabetes mellitus type 1 | 115 | 90 | -22.2 | 5 | 6 | 0.76(0.73–0.79) |
| Latvia | Diabetes mellitus type 2 | 7209 | 7559 | 4.9 | 225 | 290 | 1.09(1.00–1.17) |
| Lebanon | Diabetes mellitus | 12299 | 35830 | 191.3 | 396 | 461 | 0.60(0.55–0.64) |
| Lebanon | Diabetes mellitus type 1 | 273 | 523 | 91.8 | 6 | 6 | 0.15(0.12–0.19) |
| Lebanon | Diabetes mellitus type 2 | 12026 | 35307 | 193.6 | 391 | 455 | 0.60(0.56–0.65) |
| Lesotho | Diabetes mellitus | 5437 | 8849 | 62.8 | 414 | 556 | 1.25(1.15–1.36) |
| Lesotho | Diabetes mellitus type 1 | 108 | 111 | 2.9 | 5 | 5 | -0.04(-0.07– -0.02) |
| Lesotho | Diabetes mellitus type 2 | 5329 | 8738 | 64.0 | 408 | 550 | 1.27(1.16–1.38) |
| Liberia | Diabetes mellitus | 3276 | 10211 | 211.7 | 229 | 313 | 1.22(1.19–1.26) |
| Liberia | Diabetes mellitus type 1 | 121 | 290 | 139.9 | 5 | 5 | 0.01(-0.03–0.05) |
| Liberia | Diabetes mellitus type 2 | 3155 | 9921 | 214.5 | 224 | 308 | 1.25(1.21–1.28) |
| Libya | Diabetes mellitus | 10343 | 33874 | 227.5 | 348 | 485 | 1.19(1.14–1.23) |
| Libya | Diabetes mellitus type 1 | 301 | 460 | 52.8 | 6 | 7 | 0.26(0.23–0.29) |
| Libya | Diabetes mellitus type 2 | 10042 | 33414 | 232.8 | 342 | 479 | 1.20(1.16–1.25) |
| Lithuania | Diabetes mellitus | 9622 | 9685 | 0.7 | 227 | 261 | 0.64(0.58–0.70) |
| Lithuania | Diabetes mellitus type 1 | 169 | 132 | -22.2 | 5 | 6 | 0.61(0.56–0.66) |
| Lithuania | Diabetes mellitus type 2 | 9453 | 9554 | 1.1 | 222 | 255 | 0.64(0.58–0.70) |
| Luxembourg | Diabetes mellitus | 1614 | 3179 | 97.0 | 332 | 421 | 0.83(0.79–0.86) |
| Luxembourg | Diabetes mellitus type 1 | 31 | 59 | 87.9 | 10 | 12 | 0.94(0.84–1.04) |
| Luxembourg | Diabetes mellitus type 2 | 1582 | 3120 | 97.2 | 323 | 408 | 0.82(0.79–0.86) |
| Macedonia | Diabetes mellitus | 6054 | 10346 | 70.9 | 290 | 361 | 0.99(0.83–1.15) |
| Macedonia | Diabetes mellitus type 1 | 110 | 115 | 4.6 | 6 | 6 | 0.57(0.52–0.62) |
| Macedonia | Diabetes mellitus type 2 | 5944 | 10231 | 72.1 | 285 | 355 | 0.99(0.83–1.16) |
| Madagascar | Diabetes mellitus | 18666 | 49933 | 167.5 | 243 | 292 | 0.73(0.69–0.76) |
| Madagascar | Diabetes mellitus type 1 | 731 | 1592 | 117.7 | 5 | 5 | 0.02(0.02–0.03) |
| Madagascar | Diabetes mellitus type 2 | 17935 | 48342 | 169.5 | 237 | 287 | 0.74(0.71–0.77) |
| Malawi | Diabetes mellitus | 18215 | 39330 | 115.9 | 299 | 350 | 0.52(0.46–0.58) |
| Malawi | Diabetes mellitus type 1 | 589 | 1049 | 78.1 | 5 | 5 | -0.02(-0.04–0.00) |
| Malawi | Diabetes mellitus type 2 | 17626 | 38281 | 117.2 | 294 | 345 | 0.53(0.46–0.59) |
| Malaysia | Diabetes mellitus | 43076 | 102545 | 138.1 | 304 | 327 | 0.39(0.19–0.60) |
| Malaysia | Diabetes mellitus type 1 | 407 | 641 | 57.7 | 2 | 2 | 0.13(0.10–0.16) |
| Malaysia | Diabetes mellitus type 2 | 42670 | 101904 | 138.8 | 302 | 325 | 0.40(0.19–0.60) |
| Maldives | Diabetes mellitus | 453 | 1507 | 233.0 | 306 | 326 | 0.22(0.14–0.31) |
| Maldives | Diabetes mellitus type 1 | 5 | 9 | 69.6 | 2 | 2 | 0.17(0.14–0.20) |
| Maldives | Diabetes mellitus type 2 | 447 | 1498 | 235.0 | 304 | 324 | 0.22(0.14–0.31) |
| Mali | Diabetes mellitus | 12302 | 36175 | 194.1 | 212 | 279 | 1.07(1.04–1.09) |
| Mali | Diabetes mellitus type 1 | 526 | 1255 | 138.5 | 5 | 5 | -0.03(-0.03– -0.02) |
| Mali | Diabetes mellitus type 2 | 11776 | 34920 | 196.5 | 207 | 274 | 1.09(1.06–1.11) |
| Malta | Diabetes mellitus | 1711 | 3122 | 82.4 | 419 | 510 | 0.84(0.80–0.88) |
| Malta | Diabetes mellitus type 1 | 42 | 51 | 22.4 | 12 | 14 | 0.89(0.78–1.01) |
| Malta | Diabetes mellitus type 2 | 1670 | 3071 | 83.9 | 407 | 496 | 0.83(0.79–0.87) |
| Marshall Islands | Diabetes mellitus | 191 | 442 | 131.3 | 610 | 815 | 1.05(0.87–1.22) |
| Marshall Islands | Diabetes mellitus type 1 | 1 | 1 | 11.4 | 2 | 2 | 0.13(0.12–0.15) |
| Marshall Islands | Diabetes mellitus type 2 | 190 | 440 | 132.2 | 608 | 812 | 1.05(0.88–1.22) |
| Mauritania | Diabetes mellitus | 3280 | 8843 | 169.6 | 232 | 313 | 1.12(1.08–1.16) |
| Mauritania | Diabetes mellitus type 1 | 126 | 242 | 91.2 | 5 | 5 | 0.08(0.07–0.08) |
| Mauritania | Diabetes mellitus type 2 | 3154 | 8601 | 172.7 | 226 | 307 | 1.14(1.10–1.18) |
| Mauritius | Diabetes mellitus | 4012 | 10416 | 159.6 | 388 | 668 | 2.56(2.32–2.81) |
| Mauritius | Diabetes mellitus type 1 | 22 | 22 | -0.2 | 2 | 2 | 0.22(0.19–0.25) |
| Mauritius | Diabetes mellitus type 2 | 3990 | 10394 | 160.5 | 386 | 666 | 2.57(2.33–2.82) |
| Mexico | Diabetes mellitus | 289569 | 644529 | 122.6 | 442 | 496 | 0.18(0.05–0.32) |
| Mexico | Diabetes mellitus type 1 | 3150 | 3834 | 21.7 | 3 | 3 | 0.14(0.12–0.15) |
| Mexico | Diabetes mellitus type 2 | 286419 | 640695 | 123.7 | 439 | 493 | 0.18(0.05–0.32) |
| Micronesia | Diabetes mellitus | 447 | 829 | 85.3 | 585 | 826 | 1.35(1.19–1.51) |
| Micronesia | Diabetes mellitus type 1 | 3 | 3 | -8.3 | 2 | 2 | 0.01(-0.01–0.02) |
| Micronesia | Diabetes mellitus type 2 | 444 | 826 | 85.9 | 582 | 823 | 1.36(1.20–1.51) |
| Moldova | Diabetes mellitus | 12817 | 13699 | 6.9 | 275 | 286 | 0.21(0.18–0.24) |
| Moldova | Diabetes mellitus type 1 | 236 | 189 | -19.8 | 5 | 6 | 0.43(0.36–0.50) |
| Moldova | Diabetes mellitus type 2 | 12582 | 13510 | 7.4 | 269 | 280 | 0.21(0.17–0.24) |
| Mongolia | Diabetes mellitus | 3565 | 8506 | 138.6 | 234 | 258 | 0.50(0.43–0.56) |
| Mongolia | Diabetes mellitus type 1 | 137 | 204 | 48.5 | 6 | 6 | 0.46(0.39–0.52) |
| Mongolia | Diabetes mellitus type 2 | 3427 | 8302 | 142.2 | 228 | 252 | 0.50(0.43–0.57) |
| Montenegro | Diabetes mellitus | 2111 | 2935 | 39.0 | 319 | 363 | 0.57(0.5–0.65) |
| Montenegro | Diabetes mellitus type 1 | 39 | 40 | 3.3 | 6 | 7 | 0.64(0.57–0.70) |
| Montenegro | Diabetes mellitus type 2 | 2072 | 2894 | 39.7 | 313 | 356 | 0.57(0.50–0.65) |
| Morocco | Diabetes mellitus | 58463 | 148218 | 153.5 | 298 | 407 | 1.21(1.17–1.26) |
| Morocco | Diabetes mellitus type 1 | 1720 | 2286 | 32.9 | 6 | 6 | 0.20(0.15–0.26) |
| Morocco | Diabetes mellitus type 2 | 56744 | 145931 | 157.2 | 292 | 401 | 1.23(1.18–1.28) |
| Mozambique | Diabetes mellitus | 24179 | 60702 | 151.1 | 259 | 324 | 0.86(0.83–0.90) |
| Mozambique | Diabetes mellitus type 1 | 883 | 1886 | 113.6 | 5 | 5 | 0.02(0.01–0.04) |
| Mozambique | Diabetes mellitus type 2 | 23296 | 58816 | 152.5 | 254 | 318 | 0.88(0.84–0.91) |
| Myanmar | Diabetes mellitus | 135347 | 238565 | 76.3 | 402 | 445 | 0.40(0.33–0.47) |
| Myanmar | Diabetes mellitus type 1 | 955 | 1158 | 21.3 | 2 | 2 | 0.21(0.20–0.22) |
| Myanmar | Diabetes mellitus type 2 | 134392 | 237406 | 76.7 | 400 | 443 | 0.40(0.33–0.47) |
| Namibia | Diabetes mellitus | 3732 | 8110 | 117.3 | 383 | 435 | 0.50(0.41–0.59) |
| Namibia | Diabetes mellitus type 1 | 87 | 137 | 58.2 | 5 | 5 | -0.02(-0.03– -0.01) |
| Namibia | Diabetes mellitus type 2 | 3645 | 7973 | 118.7 | 378 | 430 | 0.51(0.42–0.60) |
| Nepal | Diabetes mellitus | 29242 | 67969 | 132.4 | 206 | 258 | 0.76(0.68–0.84) |
| Nepal | Diabetes mellitus type 1 | 1141 | 1574 | 37.9 | 5 | 5 | -0.13(-0.15– -0.11) |
| Nepal | Diabetes mellitus type 2 | 28101 | 66394 | 136.3 | 201 | 253 | 0.78(0.69–0.86) |
| Netherlands | Diabetes mellitus | 56679 | 87099 | 53.7 | 320 | 365 | 0.56(0.49–0.63) |
| Netherlands | Diabetes mellitus type 1 | 1274 | 1691 | 32.7 | 10 | 12 | 0.91(0.73–1.09) |
| Netherlands | Diabetes mellitus type 2 | 55405 | 85408 | 54.2 | 311 | 352 | 0.55(0.47–0.62) |
| New Zealand | Diabetes mellitus | 7926 | 12932 | 63.2 | 214 | 217 | -0.55(-0.87– -0.23) |
| New Zealand | Diabetes mellitus type 1 | 508 | 631 | 24.2 | 16 | 16 | 0.15(0.08–0.22) |
| New Zealand | Diabetes mellitus type 2 | 7418 | 12301 | 65.8 | 198 | 201 | -0.61(-0.96– -0.26) |
| Nicaragua | Diabetes mellitus | 8003 | 17227 | 115.3 | 303 | 296 | 0.02(-0.03–0.07) |
| Nicaragua | Diabetes mellitus type 1 | 168 | 226 | 35.0 | 3 | 3 | 0.07(0.07–0.08) |
| Nicaragua | Diabetes mellitus type 2 | 7835 | 17000 | 117.0 | 300 | 293 | 0.02(-0.03–0.07) |
| Niger | Diabetes mellitus | 9805 | 33909 | 245.9 | 205 | 272 | 1.02(0.98–1.07) |
| Niger | Diabetes mellitus type 1 | 495 | 1349 | 172.6 | 5 | 5 | 0.03(0.02–0.04) |
| Niger | Diabetes mellitus type 2 | 9310 | 32560 | 249.7 | 200 | 266 | 1.05(1.00–1.09) |
| Nigeria | Diabetes mellitus | 96306 | 272617 | 183.1 | 154 | 203 | 0.82(0.75–0.90) |
| Nigeria | Diabetes mellitus type 1 | 5351 | 12698 | 137.3 | 5 | 5 | 0.07(0.06–0.08) |
| Nigeria | Diabetes mellitus type 2 | 90954 | 259919 | 185.8 | 149 | 198 | 0.85(0.77–0.93) |
| North Korea | Diabetes mellitus | 39079 | 72067 | 84.4 | 189 | 235 | 0.87(0.78–0.96) |
| North Korea | Diabetes mellitus type 1 | 496 | 595 | 20.1 | 2 | 3 | 0.39(0.34–0.44) |
| North Korea | Diabetes mellitus type 2 | 38583 | 71472 | 85.2 | 187 | 232 | 0.87(0.78–0.96) |
| Northern Mariana Islands | Diabetes mellitus | 197 | 316 | 60.6 | 453 | 566 | 0.89(0.78–1.00) |
| Northern Mariana Islands | Diabetes mellitus type 1 | 1 | 1 | -12.8 | 2 | 2 | 0.25(0.13–0.37) |
| Northern Mariana Islands | Diabetes mellitus type 2 | 196 | 315 | 60.9 | 451 | 564 | 0.89(0.78–1.00) |
| Norway | Diabetes mellitus | 16812 | 24661 | 46.7 | 316 | 349 | 0.54(0.44–0.64) |
| Norway | Diabetes mellitus type 1 | 571 | 880 | 54.1 | 15 | 20 | 1.34(1.08–1.6) |
| Norway | Diabetes mellitus type 2 | 16241 | 23781 | 46.4 | 302 | 330 | 0.50(0.40–0.60) |
| Oman | Diabetes mellitus | 4346 | 19841 | 356.5 | 316 | 449 | 1.29(1.21–1.36) |
| Oman | Diabetes mellitus type 1 | 136 | 281 | 107.2 | 6 | 6 | 0.06(-0.01–0.14) |
| Oman | Diabetes mellitus type 2 | 4210 | 19559 | 364.6 | 310 | 442 | 1.31(1.23–1.38) |
| Pakistan | Diabetes mellitus | 189040 | 617208 | 226.5 | 239 | 362 | 1.80(1.67–1.94) |
| Pakistan | Diabetes mellitus type 1 | 6588 | 12297 | 86.7 | 5 | 5 | -0.05(-0.06– -0.04) |
| Pakistan | Diabetes mellitus type 2 | 182452 | 604911 | 231.5 | 234 | 356 | 1.84(1.70–1.98) |
| Palestine | Diabetes mellitus | 4725 | 16627 | 251.9 | 376 | 446 | 0.66(0.59–0.72) |
| Palestine | Diabetes mellitus type 1 | 143 | 328 | 130.2 | 6 | 6 | 0.07(0.04–0.09) |
| Palestine | Diabetes mellitus type 2 | 4582 | 16299 | 255.7 | 371 | 440 | 0.67(0.6–0.73) |
| Panama | Diabetes mellitus | 4600 | 10994 | 139.0 | 232 | 274 | 0.53(0.48–0.58) |
| Panama | Diabetes mellitus type 1 | 86 | 126 | 45.5 | 3 | 3 | 0.12(0.11–0.12) |
| Panama | Diabetes mellitus type 2 | 4513 | 10868 | 140.8 | 229 | 271 | 0.54(0.49–0.58) |
| Papua New Guinea | Diabetes mellitus | 16763 | 49955 | 198.0 | 513 | 614 | 0.65(0.60–0.69) |
| Papua New Guinea | Diabetes mellitus type 1 | 113 | 255 | 126.4 | 2 | 2 | 0.12(0.11–0.13) |
| Papua New Guinea | Diabetes mellitus type 2 | 16651 | 49701 | 198.5 | 511 | 612 | 0.65(0.60–0.70) |
| Paraguay | Diabetes mellitus | 8191 | 22075 | 169.5 | 271 | 348 | 1.14(0.97–1.31) |
| Paraguay | Diabetes mellitus type 1 | 216 | 340 | 57.1 | 5 | 5 | 0.08(0.06–0.10) |
| Paraguay | Diabetes mellitus type 2 | 7975 | 21736 | 172.6 | 266 | 343 | 1.16(0.98–1.33) |
| Peru | Diabetes mellitus | 26970 | 65597 | 143.2 | 162 | 203 | 0.76(0.63–0.88) |
| Peru | Diabetes mellitus type 1 | 977 | 1352 | 38.4 | 4 | 4 | 0.13(0.12–0.14) |
| Peru | Diabetes mellitus type 2 | 25993 | 64245 | 147.2 | 158 | 199 | 0.77(0.64–0.90) |
| Philippines | Diabetes mellitus | 121928 | 405557 | 232.6 | 255 | 425 | 1.88(1.73–2.03) |
| Philippines | Diabetes mellitus type 1 | 1527 | 2311 | 51.4 | 2 | 2 | 0.07(0.06–0.08) |
| Philippines | Diabetes mellitus type 2 | 120402 | 403246 | 234.9 | 253 | 423 | 1.89(1.74–2.05) |
| Poland | Diabetes mellitus | 111814 | 155850 | 39.4 | 260 | 307 | 0.56(0.47–0.64) |
| Poland | Diabetes mellitus type 1 | 1747 | 1822 | 4.3 | 5 | 6 | 0.95(0.89–1.01) |
| Poland | Diabetes mellitus type 2 | 110067 | 154028 | 39.9 | 255 | 301 | 0.55(0.47–0.64) |
| Portugal | Diabetes mellitus | 45815 | 71277 | 55.6 | 363 | 453 | 0.75(0.66–0.83) |
| Portugal | Diabetes mellitus type 1 | 871 | 1052 | 20.8 | 10 | 13 | 1.08(0.99–1.16) |
| Portugal | Diabetes mellitus type 2 | 44945 | 70225 | 56.3 | 353 | 440 | 0.74(0.65–0.82) |
| Puerto Rico | Diabetes mellitus | 13396 | 19743 | 47.4 | 366 | 429 | 0.65(0.51–0.79) |
| Puerto Rico | Diabetes mellitus type 1 | 212 | 196 | -7.5 | 6 | 6 | -0.01(-0.06–0.05) |
| Puerto Rico | Diabetes mellitus type 2 | 13184 | 19548 | 48.3 | 360 | 423 | 0.66(0.52–0.80) |
| Qatar | Diabetes mellitus | 1930 | 15208 | 688.2 | 654 | 572 | -0.55(-0.76– -0.33) |
| Qatar | Diabetes mellitus type 1 | 27 | 162 | 493.5 | 6 | 6 | 0.13(0.08–0.18) |
| Qatar | Diabetes mellitus type 2 | 1902 | 15046 | 691.0 | 648 | 566 | -0.55(-0.77– -0.34) |
| Republic of Congo | Diabetes mellitus | 6874 | 18701 | 172.1 | 410 | 479 | 0.58(0.54–0.63) |
| Republic of Congo | Diabetes mellitus type 1 | 149 | 294 | 97.8 | 5 | 5 | 0.13(0.10–0.15) |
| Republic of Congo | Diabetes mellitus type 2 | 6725 | 18407 | 173.7 | 405 | 474 | 0.59(0.55–0.63) |
| Romania | Diabetes mellitus | 50768 | 60299 | 18.8 | 194 | 236 | 0.85(0.81–0.89) |
| Romania | Diabetes mellitus type 1 | 1022 | 864 | -15.5 | 5 | 6 | 0.91(0.83–0.99) |
| Romania | Diabetes mellitus type 2 | 49746 | 59435 | 19.5 | 189 | 230 | 0.85(0.81–0.89) |
| Russian | Diabetes mellitus | 405751 | 485104 | 19.6 | 232 | 246 | 0.25(0.22–0.29) |
| Russian | Diabetes mellitus type 1 | 9560 | 10330 | 8.1 | 7 | 8 | 0.67(0.62–0.73) |
| Russian | Diabetes mellitus type 2 | 396192 | 474774 | 19.8 | 225 | 238 | 0.24(0.20–0.28) |
| Rwanda | Diabetes mellitus | 11799 | 22808 | 93.3 | 268 | 268 | -0.2(-0.27– -0.12) |
| Rwanda | Diabetes mellitus type 1 | 457 | 778 | 70.3 | 5 | 6 | -0.01(-0.04–0.01) |
| Rwanda | Diabetes mellitus type 2 | 11342 | 22029 | 94.2 | 262 | 263 | -0.20(-0.27–-0.12) |
| Saint Lucia | Diabetes mellitus | 464 | 835 | 80.0 | 425 | 408 | -0.26(-0.33– -0.19) |
| Saint Lucia | Diabetes mellitus type 1 | 6 | 7 | 10.9 | 4 | 4 | 0.24(0.21–0.27) |
| Saint Lucia | Diabetes mellitus type 2 | 458 | 828 | 80.9 | 420 | 404 | -0.26(-0.33– -0.20) |
| Saint Vincent | Diabetes mellitus | 374 | 625 | 67.0 | 426 | 494 | 0.42(0.35–0.50) |
| Saint Vincent | Diabetes mellitus type 1 | 5 | 5 | -8.9 | 4 | 4 | 0.23(0.21–0.25) |
| Saint Vincent | Diabetes mellitus type 2 | 369 | 620 | 68.1 | 422 | 490 | 0.43(0.35–0.50) |
| Samoa | Diabetes mellitus | 519 | 1037 | 99.9 | 415 | 601 | 1.46(1.27–1.65) |
| Samoa | Diabetes mellitus type 1 | 4 | 5 | 17.3 | 2 | 2 | 0.15(0.12–0.19) |
| Samoa | Diabetes mellitus type 2 | 514 | 1032 | 100.6 | 412 | 599 | 1.47(1.28–1.66) |
| Sao Tome and Principe | Diabetes mellitus | 153 | 390 | 153.9 | 176 | 253 | 1.39(1.36–1.42) |
| Sao Tome and Principe | Diabetes mellitus type 1 | 7 | 12 | 63.1 | 5 | 5 | 0.13(0.10–0.15) |
| Sao Tome and Principe | Diabetes mellitus type 2 | 146 | 377 | 158.5 | 171 | 248 | 1.43(1.40–1.46) |
| Saudi Arabia | Diabetes mellitus | 40150 | 158197 | 294.0 | 345 | 439 | 1.06(0.92–1.20) |
| Saudi Arabia | Diabetes mellitus type 1 | 1535 | 3170 | 106.6 | 9 | 9 | 0.29(0.18–0.40) |
| Saudi Arabia | Diabetes mellitus type 2 | 38616 | 155027 | 301.5 | 337 | 430 | 1.08(0.94–1.22) |
| Senegal | Diabetes mellitus | 12099 | 35567 | 194.0 | 246 | 337 | 1.22(1.16–1.28) |
| Senegal | Diabetes mellitus type 1 | 470 | 898 | 91.0 | 5 | 5 | 0.08(0.07–0.09) |
| Senegal | Diabetes mellitus type 2 | 11629 | 34669 | 198.1 | 240 | 331 | 1.24(1.18–1.31) |
| Serbia | Diabetes mellitus | 34094 | 43163 | 26.6 | 306 | 366 | 0.82(0.72–0.93) |
| Serbia | Diabetes mellitus type 1 | 516 | 512 | -0.8 | 6 | 7 | 0.50(0.48–0.52) |
| Serbia | Diabetes mellitus type 2 | 33578 | 42652 | 27.0 | 300 | 359 | 0.83(0.72–0.94) |
| Seychelles | Diabetes mellitus | 176 | 464 | 163.4 | 268 | 410 | 1.48(1.41–1.55) |
| Seychelles | Diabetes mellitus type 1 | 2 | 2 | 22.3 | 2 | 2 | 0.27(0.25–0.29) |
| Seychelles | Diabetes mellitus type 2 | 175 | 462 | 164.6 | 266 | 408 | 1.49(1.41–1.56) |
| Sierra Leone | Diabetes mellitus | 5339 | 15017 | 181.3 | 201 | 278 | 1.33(1.23–1.43) |
| Sierra Leone | Diabetes mellitus type 1 | 225 | 475 | 111.2 | 5 | 5 | 0.00(-0.02–0.01) |
| Sierra Leone | Diabetes mellitus type 2 | 5114 | 14542 | 184.4 | 196 | 273 | 1.36(1.26–1.46) |
| Singapore | Diabetes mellitus | 11313 | 17943 | 58.6 | 358 | 266 | -0.72(-0.91– -0.52) |
| Singapore | Diabetes mellitus type 1 | 294 | 385 | 30.8 | 12 | 12 | 0.00(-0.02–0.02) |
| Singapore | Diabetes mellitus type 2 | 11018 | 17558 | 59.4 | 347 | 254 | -0.74(-0.94– -0.54) |
| Slovakia | Diabetes mellitus | 14038 | 18904 | 34.7 | 243 | 260 | 0.30(0.26–0.33) |
| Slovakia | Diabetes mellitus type 1 | 255 | 275 | 7.9 | 5 | 6 | 0.83(0.79–0.86) |
| Slovakia | Diabetes mellitus type 2 | 13783 | 18629 | 35.2 | 238 | 254 | 0.28(0.25–0.32) |
| Slovenia | Diabetes mellitus | 5984 | 7818 | 30.7 | 251 | 272 | 0.20(0.01–0.39) |
| Slovenia | Diabetes mellitus type 1 | 95 | 103 | 8.5 | 5 | 6 | 0.79(0.76–0.81) |
| Slovenia | Diabetes mellitus type 2 | 5889 | 7716 | 31.0 | 246 | 265 | 0.19(0.00–0.38) |
| Solomon Islands | Diabetes mellitus | 1175 | 3502 | 198.1 | 476 | 644 | 1.09(1.03–1.15) |
| Solomon Islands | Diabetes mellitus type 1 | 9 | 17 | 84.4 | 2 | 2 | 0.16(0.15–0.17) |
| Solomon Islands | Diabetes mellitus type 2 | 1165 | 3485 | 199.0 | 473 | 642 | 1.09(1.03–1.15) |
| Somalia | Diabetes mellitus | 12323 | 34769 | 182.1 | 286 | 336 | 0.61(0.58–0.64) |
| Somalia | Diabetes mellitus type 1 | 461 | 1079 | 133.8 | 6 | 6 | -0.07(-0.17–0.02) |
| Somalia | Diabetes mellitus type 2 | 11862 | 33691 | 184.0 | 280 | 331 | 0.62(0.59–0.65) |
| South Africa | Diabetes mellitus | 92517 | 231672 | 150.4 | 324 | 445 | 1.30(1.15–1.46) |
| South Africa | Diabetes mellitus type 1 | 2165 | 2975 | 37.4 | 5 | 5 | -0.01(-0.02–0.00) |
| South Africa | Diabetes mellitus type 2 | 90352 | 228697 | 153.1 | 318 | 440 | 1.32(1.16–1.48) |
| South Korea | Diabetes mellitus | 126104 | 242971 | 92.7 | 306 | 323 | 0.46(0.34–0.58) |
| South Korea | Diabetes mellitus type 1 | 4839 | 3663 | -24.3 | 12 | 12 | 0.16(0.15–0.17) |
| South Korea | Diabetes mellitus type 2 | 121265 | 239308 | 97.3 | 294 | 310 | 0.47(0.35–0.60) |
| South Sudan | Diabetes mellitus | 10301 | 20658 | 100.6 | 279 | 336 | 0.68(0.64–0.72) |
| South Sudan | Diabetes mellitus type 1 | 359 | 623 | 73.2 | 5 | 5 | 0.05(0.03–0.07) |
| South Sudan | Diabetes mellitus type 2 | 9942 | 20036 | 101.5 | 274 | 330 | 0.69(0.65–0.73) |
| Spain | Diabetes mellitus | 125595 | 188732 | 50.3 | 250 | 286 | 0.30(0.16–0.45) |
| Spain | Diabetes mellitus type 1 | 3377 | 4800 | 42.1 | 9 | 13 | 1.25(1.22–1.28) |
| Spain | Diabetes mellitus type 2 | 122218 | 183932 | 50.5 | 241 | 273 | 0.26(0.11–0.41) |
| Sri Lanka | Diabetes mellitus | 40212 | 109684 | 172.8 | 272 | 450 | 2.10(1.95–2.26) |
| Sri Lanka | Diabetes mellitus type 1 | 371 | 422 | 13.6 | 2 | 2 | 0.18(0.16–0.19) |
| Sri Lanka | Diabetes mellitus type 2 | 39841 | 109262 | 174.3 | 270 | 448 | 2.11(1.96–2.27) |
| Sudan | Diabetes mellitus | 35500 | 95047 | 167.7 | 258 | 332 | 1.07(0.95–1.18) |
| Sudan | Diabetes mellitus type 1 | 1278 | 2660 | 108.1 | 5 | 5 | 0.16(0.12–0.20) |
| Sudan | Diabetes mellitus type 2 | 34222 | 92387 | 170.0 | 253 | 326 | 1.08(0.96–1.20) |
| Suriname | Diabetes mellitus | 1056 | 2564 | 142.9 | 314 | 419 | 1.06(1.00–1.11) |
| Suriname | Diabetes mellitus type 1 | 18 | 24 | 38.7 | 4 | 4 | 0.22(0.19–0.26) |
| Suriname | Diabetes mellitus type 2 | 1038 | 2540 | 144.7 | 310 | 414 | 1.07(1.01–1.12) |
| Swaziland | Diabetes mellitus | 2458 | 5642 | 129.5 | 506 | 657 | 1.17(1.02–1.33) |
| Swaziland | Diabetes mellitus type 1 | 50 | 66 | 31.1 | 5 | 5 | -0.05(-0.06– -0.03) |
| Swaziland | Diabetes mellitus type 2 | 2408 | 5576 | 131.6 | 501 | 652 | 1.18(1.03–1.34) |
| Sweden | Diabetes mellitus | 30699 | 49609 | 61.6 | 266 | 347 | 0.95(0.91–0.98) |
| Sweden | Diabetes mellitus type 1 | 1129 | 1229 | 8.8 | 15 | 14 | -0.40(-0.77– -0.03) |
| Sweden | Diabetes mellitus type 2 | 29570 | 48380 | 63.6 | 251 | 333 | 1.02(1.00–1.04) |
| Switzerland | Diabetes mellitus | 25794 | 39421 | 52.8 | 294 | 328 | 0.38(0.34–0.41) |
| Switzerland | Diabetes mellitus type 1 | 622 | 906 | 45.8 | 10 | 13 | 0.92(0.81–1.02) |
| Switzerland | Diabetes mellitus type 2 | 25173 | 38515 | 53.0 | 283 | 315 | 0.36(0.31–0.40) |
| Syria | Diabetes mellitus | 21843 | 54685 | 150.4 | 258 | 331 | 0.76(0.68–0.84) |
| Syria | Diabetes mellitus type 1 | 904 | 1251 | 38.4 | 6 | 6 | 0.15(0.08–0.22) |
| Syria | Diabetes mellitus type 2 | 20939 | 53434 | 155.2 | 252 | 325 | 0.77(0.69–0.85) |
| Tajikistan | Diabetes mellitus | 12144 | 32287 | 165.9 | 324 | 401 | 0.78(0.69–0.87) |
| Tajikistan | Diabetes mellitus type 1 | 338 | 596 | 76.3 | 6 | 6 | 0.28(0.25–0.31) |
| Tajikistan | Diabetes mellitus type 2 | 11806 | 31691 | 168.4 | 318 | 395 | 0.79(0.70–0.88) |
| Tanzania | Diabetes mellitus | 32907 | 91661 | 178.6 | 208 | 262 | 0.87(0.82–0.92) |
| Tanzania | Diabetes mellitus type 1 | 1593 | 3302 | 107.3 | 5 | 5 | 0.04(0.03–0.05) |
| Tanzania | Diabetes mellitus type 2 | 31314 | 88359 | 182.2 | 202 | 257 | 0.89(0.84–0.94) |
| Thailand | Diabetes mellitus | 114781 | 238285 | 107.6 | 223 | 268 | 0.17(-0.08–0.42) |
| Thailand | Diabetes mellitus type 1 | 1196 | 1178 | -1.6 | 2 | 2 | 0.17(0.15–0.19) |
| Thailand | Diabetes mellitus type 2 | 113585 | 237108 | 108.8 | 221 | 266 | 0.17(-0.08–0.42) |
| Timor-Leste | Diabetes mellitus | 1665 | 3905 | 134.5 | 293 | 378 | 0.84(0.79–0.90) |
| Timor-Leste | Diabetes mellitus type 1 | 19 | 31 | 63.8 | 2 | 2 | 0.00(-0.03–0.03) |
| Timor-Leste | Diabetes mellitus type 2 | 1646 | 3873 | 135.3 | 291 | 376 | 0.85(0.79–0.90) |
| Tobago | Diabetes mellitus | 5841 | 9087 | 55.6 | 561 | 553 | -0.09(-0.16– -0.01) |
| Tobago | Diabetes mellitus type 1 | 55 | 54 | -0.4 | 4 | 4 | 0.12(0.09–0.15) |
| Tobago | Diabetes mellitus type 2 | 5786 | 9033 | 56.1 | 556 | 549 | -0.09(-0.16– -0.01) |
| Togo | Diabetes mellitus | 4258 | 14499 | 240.5 | 193 | 267 | 1.20(1.15–1.26) |
| Togo | Diabetes mellitus type 1 | 233 | 464 | 98.9 | 5 | 6 | 0.11(0.09–0.13) |
| Togo | Diabetes mellitus type 2 | 4024 | 14035 | 248.7 | 188 | 262 | 1.23(1.17–1.29) |
| Tonga | Diabetes mellitus | 428 | 688 | 60.7 | 581 | 730 | 1.07(0.99–1.16) |
| Tonga | Diabetes mellitus type 1 | 3 | 3 | 3.2 | 2 | 2 | 0.20(0.18–0.23) |
| Tonga | Diabetes mellitus type 2 | 425 | 685 | 61.1 | 578 | 728 | 1.08(0.99–1.17) |
| Trinidad | Diabetes mellitus | 5841 | 9087 | 55.6 | 561 | 553 | -0.09(-0.16– -0.01) |
| Trinidad | Diabetes mellitus type 1 | 55 | 54 | -0.4 | 4 | 4 | 0.12(0.09–0.15) |
| Trinidad | Diabetes mellitus type 2 | 5786 | 9033 | 56.1 | 556 | 549 | -0.09(-0.16– -0.01) |
| Tunisia | Diabetes mellitus | 16300 | 42884 | 163.1 | 244 | 337 | 1.12(1.06–1.18) |
| Tunisia | Diabetes mellitus type 1 | 508 | 635 | 25.0 | 5 | 6 | 0.46(0.42–0.50) |
| Tunisia | Diabetes mellitus type 2 | 15791 | 42249 | 167.6 | 239 | 331 | 1.13(1.07–1.20) |
| Turkey | Diabetes mellitus | 130147 | 262548 | 101.7 | 279 | 296 | 0.39(0.26–0.52) |
| Turkey | Diabetes mellitus type 1 | 3648 | 4558 | 24.9 | 6 | 6 | 0.2(0.15–0.25) |
| Turkey | Diabetes mellitus type 2 | 126499 | 257990 | 104.0 | 273 | 290 | 0.39(0.26–0.52) |
| Turkmenistan | Diabetes mellitus | 8745 | 19568 | 123.8 | 322 | 385 | 0.76(0.68–0.84) |
| Turkmenistan | Diabetes mellitus type 1 | 232 | 303 | 30.5 | 6 | 6 | 0.29(0.25–0.33) |
| Turkmenistan | Diabetes mellitus type 2 | 8512 | 19265 | 126.3 | 317 | 379 | 0.76(0.68–0.84) |
| Uganda | Diabetes mellitus | 25404 | 67400 | 165.3 | 253 | 295 | 0.59(0.51–0.66) |
| Uganda | Diabetes mellitus type 1 | 1066 | 2443 | 129.3 | 5 | 5 | 0.04(0.03–0.05) |
| Uganda | Diabetes mellitus type 2 | 24339 | 64956 | 166.9 | 248 | 290 | 0.60(0.52–0.68) |
| UK | Diabetes mellitus | 143928 | 268256 | 86.4 | 192 | 300 | 1.59(1.48–1.7) |
| UK | Diabetes mellitus type 1 | 5080 | 6663 | 31.2 | 10 | 12 | 0.65(0.52–0.78) |
| UK | Diabetes mellitus type 2 | 138848 | 261593 | 88.4 | 182 | 287 | 1.64(1.52–1.76) |
| Ukraine | Diabetes mellitus | 146598 | 150261 | 2.5 | 236 | 254 | 0.21(0.14–0.27) |
| Ukraine | Diabetes mellitus type 1 | 2538 | 2175 | -14.3 | 5 | 6 | 0.44(0.37–0.51) |
| Ukraine | Diabetes mellitus type 2 | 144060 | 148086 | 2.8 | 231 | 248 | 0.20(0.13–0.27) |
| United Arab Emirates | Diabetes mellitus | 5989 | 63725 | 964.1 | 430 | 535 | 0.99(0.82–1.16) |
| United Arab Emirates | Diabetes mellitus type 1 | 118 | 570 | 382.0 | 6 | 7 | 0.28(0.16–0.40) |
| United Arab Emirates | Diabetes mellitus type 2 | 5870 | 63155 | 975.8 | 424 | 528 | 1.00(0.83–1.17) |
| Uruguay | Diabetes mellitus | 13593 | 15323 | 12.7 | 392 | 374 | -0.19(-0.21– -0.16) |
| Uruguay | Diabetes mellitus type 1 | 578 | 585 | 1.1 | 19 | 18 | -0.10(-0.12– -0.09) |
| Uruguay | Diabetes mellitus type 2 | 13015 | 14738 | 13.2 | 373 | 356 | -0.19(-0.22– -0.16) |
| USA | Diabetes mellitus | 634673 | 1388743 | 118.8 | 230 | 324 | 2.27(1.89–2.65) |
| USA | Diabetes mellitus type 1 | 34301 | 43714 | 27.4 | 15 | 16 | 0.51(0.39–0.62) |
| USA | Diabetes mellitus type 2 | 600372 | 1345030 | 124.0 | 215 | 309 | 2.38(1.97–2.78) |
| Uzbekistan | Diabetes mellitus | 48882 | 133200 | 172.5 | 315 | 417 | 1.22(1.11–1.33) |
| Uzbekistan | Diabetes mellitus type 1 | 1288 | 1942 | 50.8 | 5 | 6 | 0.26(0.22–0.31) |
| Uzbekistan | Diabetes mellitus type 2 | 47594 | 131259 | 175.8 | 310 | 412 | 1.23(1.12–1.35) |
| Vanuatu | Diabetes mellitus | 499 | 1578 | 216.3 | 444 | 648 | 1.46(1.42–1.50) |
| Vanuatu | Diabetes mellitus type 1 | 4 | 8 | 85.6 | 2 | 2 | 0.06(0.05–0.07) |
| Vanuatu | Diabetes mellitus type 2 | 495 | 1570 | 217.4 | 442 | 645 | 1.47(1.43–1.51) |
| Venezuela | Diabetes mellitus | 39527 | 95204 | 140.9 | 269 | 301 | 0.32(0.18–0.46) |
| Venezuela | Diabetes mellitus type 1 | 719 | 966 | 34.4 | 3 | 3 | 0.04(0.03–0.04) |
| Venezuela | Diabetes mellitus type 2 | 38808 | 94238 | 142.8 | 265 | 298 | 0.32(0.18–0.47) |
| Vietnam | Diabetes mellitus | 139087 | 354953 | 155.2 | 263 | 343 | 0.92(0.88–0.96) |
| Vietnam | Diabetes mellitus type 1 | 1543 | 1809 | 17.2 | 2 | 2 | 0.11(0.10–0.12) |
| Vietnam | Diabetes mellitus type 2 | 137544 | 353144 | 156.8 | 261 | 341 | 0.93(0.88–0.97) |
| Virgin Islands | Diabetes mellitus | 340 | 493 | 45.1 | 321 | 367 | 0.45(0.41–0.49) |
| Virgin Islands | Diabetes mellitus type 1 | 6 | 5 | -11.6 | 5 | 6 | 0.12(0.05–0.20) |
| Virgin Islands | Diabetes mellitus type 2 | 334 | 488 | 46.1 | 316 | 362 | 0.46(0.41–0.5) |
| Yemen | Diabetes mellitus | 19118 | 63749 | 233.5 | 233 | 301 | 0.83(0.70–0.95) |
| Yemen | Diabetes mellitus type 1 | 1007 | 2195 | 118.0 | 6 | 6 | 0.05(0.00–0.10) |
| Yemen | Diabetes mellitus type 2 | 18112 | 61555 | 239.9 | 227 | 295 | 0.84(0.72–0.97) |
| Zambia | Diabetes mellitus | 13392 | 34710 | 159.2 | 286 | 323 | 0.34(0.28–0.39) |
| Zambia | Diabetes mellitus type 1 | 490 | 1074 | 119.0 | 5 | 5 | 0.05(0.04–0.07) |
| Zambia | Diabetes mellitus type 2 | 12902 | 33636 | 160.7 | 281 | 318 | 0.34(0.28–0.40) |
| Zimbabwe | Diabetes mellitus | 20208 | 47624 | 135.7 | 306 | 450 | 1.62(1.49–1.75) |
| Zimbabwe | Diabetes mellitus type 1 | 645 | 876 | 35.9 | 5 | 5 | -0.04(-0.08– -0.01) |
| Zimbabwe | Diabetes mellitus type 2 | 19563 | 46747 | 139.0 | 301 | 445 | 1.64(1.51–1.77) |

ASR, age standardized rate; CI, confidence interval; EAPC, estimated annual percentage change; UI, uncertainty interval.

**^#^** The ASR was deemed to be in an increasing trend if the EAPC and the lower boundary of its 95% CI were both >0%; the ASR was in a decreasing trend if the EAPC estimation and the

upper boundary of its 95% CI were both <0%; otherwise, the ASR was deemed to be uncertain over time.
